# Genome-wide association study of asthma with high treatment burden and/or worse outcomes defined using electronic healthcare data in UK Biobank

**DOI:** 10.1183/23120541.00327-2026

**Published:** 2026-09-01

**Authors:** Noemi N. Piga, Michael A. Portelli, Nick Shrine, Jing Chen, Richard Packer, Kayesha Coley, Alexander T. Williams, Chiara Batini, Catherine John, Sharon M. Lutz, Kirsten R. Voorhies, Albert M. Levin, Nazanin Zounemat-Kermani, James E. Gern, Diane R. Gold, Tina V. Hartert, Daniel J. Jackson, Christine C. Johnson, Gurjit K. Khurana Hershey, Rachel L. Miller, Christine M. Seroogy, Edward M. Zoratti, Don D. Sin, Maarten van den Berge, Yohan Bossé, Robert J. Hall, Dominick Shaw, Zara E. K. Pogson, Andrew Fogarty, Liam G. Heaney, Adel H. Mansur, Rekha Chaudhuri, Neil C. Thomson, John W. Holloway, Kian Fan Chung, John D. Blakey, Louise V. Wain, Carole Ober, Ann C. Wu, Ian M. Adcock, Ian P. Hall, Christopher E. Brightling, Glenda Lassi, Ian Sayers, Katherine A. Fawcett

**Affiliations:** 1 Division of Public Health and Epidemiology, School of Medical Sciences, University of Leicester, Leicester, UK; 2 Centre for Respiratory Research, NIHR Biomedical Research Centre, School of Medicine, Biodiscovery Institute, University of Nottingham, Nottingham, UK; 3 Division of Pharmacy and Optometry, School of Healthcare, College of Life Sciences, University of Leicester, Leicester, UK; 4 University Hospitals of Leicester NHS Trust, Leicester, UK; 5 Department of Population Medicine, Harvard Pilgrim Health Care Institute, Boston, MA, USA; 6 Department of Biostatistics, Harvard T.H. Chan School of Public Health, Boston, MA, USA; 7 Department of Public Health Sciences, Henry Ford Health, Detroit, MI, USA; 8 Center for Bioinformatics, Henry Ford Health, Detroit, MI, USA; 9 Data Science Institute, Imperial College, London, UK; 10 Department of Pediatrics, University of Wisconsin School of Medicine and Public Health, Madison, WI, USA; 11 Channing Division of Network Medicine, Brigham and Women's Hospital and Harvard Medical School, Boston, MA, USA; 12 Department of Environmental Health, Harvard T.H. Chan School of Public Health, Boston, MA, USA; 13 Departments of Medicine and Pediatrics, Vanderbilt University Medical Center, Nashville, TN, USA; 14 University of Wisconsin School of Medicine and Public Heath, Madison, WI, USA; 15 Cincinnati Children's Hospital, Division of Asthma Research, Cincinnati, OH, USA; 16 University of Cincinnati College of Medicine, Department of Pediatrics, University of Cincinnati, Cincinnati, OH, USA; 17 Icahn School of Medicine at Mount Sinai, New York, NY, USA; 18 Department of Internal Medicine, Division of Allergy and Clinical Immunology, Henry Ford Health, Detroit, MI, USA; 19 Centre for Heart Lung Innovation, St Paul's Hospital, University of British Columbia, Vancouver, BC, Canada; 20 Department of Pulmonary Diseases, University Medical Center Groningen, University of Groningen, Groningen, The Netherlands; 21 Institut Universitaire de Cardiologie et de Pneumologie de Québec – Université Laval, Quebec City, QC, Canada; 22 Department of Respiratory Sciences, University of Leicester, Leicester, UK; 23 Institute for Lung Health, Leicester NIHR BRC, University of Leicester, Leicester, UK; 24 Department of Respiratory Medicine, Lincoln County Hospital, Lincolnshire Community and Hospitals NHS Group, Lincoln, UK; 25 Division of Lifespan and Population Sciences, University of Nottingham. Nottingham NIHR Biomedical Research Centre, Nottingham, UK; 26 Wellcome-Wolfson Institute for Experimental Medicine, Queens University Belfast, Belfast, UK; 27 Respiratory Medicine, Birmingham Heartlands Hospital and University of Birmingham, Birmingham, UK; 28 School of Infection and Immunity, University of Glasgow, Glasgow, UK; 29 Human Development and Health, Faculty of Medicine, University of Southampton, Southampton, UK; 30 NIHR Southampton Biomedical Research Centre, University Hospitals Southampton, Southampton, UK; 31 The National Heart and Lung Institute, Imperial College, London, UK; 32 Respiratory Medicine, Sir Charles Gairdner Hospital, Perth, WA, Australia; 33 Medical School, Curtin University, Perth, WA, Australia; 34 Department of Human Genetics, The University of Chicago, Chicago, IL, USA; 35 Translational Science & Experimental Medicine, Research and Early Development, Respiratory and Immunology, BioPharmaceuticals R&D, AstraZeneca, Cambridge, UK

## Abstract

**Background:**

In ∼10% of asthma patients, symptoms remain uncontrolled despite maximal treatment, representing an unmet clinical need. The causal variants, genes and pathways underlying genetic risk factors have not been fully elucidated, and it is unclear whether there are unique genetic risk factors for this asthma subtype.

**Methods:**

We used electronic healthcare records linked to UK Biobank to identify asthma patients with high treatment burden and/or worse outcomes. We performed a genome-wide association study (GWAS) with this case population and healthy controls. We sought replication for associated (p≤5×10^−6^) signals in four independent studies (12 152 cases and 32 316 controls). Replicated signals were fine-mapped and linked to genes and pathways.

**Results:**

In total, 7681 participants met our case definition and showed enrichment for adult-onset asthma, female gender and higher body mass index compared to asthma individuals not meeting case criteria. GWAS with 7681 cases and 38 405 controls revealed 21 reproducible association signals that had previously been associated with asthma, but had a larger effect size in our study. Variant-to-gene mapping highlighted 85 candidate genes, five of which were considered high confidence (*BACH2*, *D2HGDH*, *IL1RL1*, *RPS26*, *SMAD3*).

**Conclusion:**

We present the first use of electronic healthcare records in UK Biobank to identify a subtype of asthma enriched for patients with high treatment burden and/or worse outcomes. Our findings support the role of known asthma genes, highlighting genetic risk variants with stronger effect in these groups of patients. The prioritised genes provide potential therapeutic opportunities for this difficult-to-treat patient population.

## Shareable abstract

**A GWAS of asthma with high treatment burden and/or worse outcomes in UK Biobank revealed no loci specific to this asthma subtype. 21 reproducible loci highlighted potential causal variants and genes providing a therapeutic opportunity.** https://bit.ly/4rWDWq7

## Introduction

The majority of asthma patients can control their symptoms with existing treatments; however, 3–10% have a more severe form of asthma and struggle to manage their condition despite optimal use of therapy [1]. These patients experience more frequent exacerbations, hospitalisation, and have a higher burden of asthma-related comorbidities and side-effects due to long-term steroid use [2]. Recently developed biological therapies have improved management; however, not all patients can access these medications, are eligible or respond well, leaving an unmet clinical need [3].

Genome-wide association studies (GWAS) have discovered almost 200 genetic risk loci for asthma [4]. However, these studies have predominantly focused on all-comer asthma defined by self-reported doctor-diagnosis. A Danish twin study estimated a heritability of 24% for asthma symptom severity, suggesting that more severe endotypes of asthma might have distinct genetic risk factors [5]. Genetic studies of more severe forms of asthma, either defined by treatment burden or exacerbation severity and/or frequency, have highlighted shared genomic regions with all-comer asthma [6–9], including *IL1RL1/IL18R1*, *TSLP*, *HLA-DQA1*, *BACH2*, *C11orf30*, *RAD51B* and the *ORMDL3/GSDMB* locus, suggesting a key role for the immune system and DNA damage response. The largest study to date of moderate-to-severe asthma defined using self-reported medications in UK Biobank, along with the Genetics of Asthma Severity and Phenotypes (GASP) and Unbiased Biomarkers for the Prediction of Respiratory Disease Outcomes (U-BIOPRED) studies, identified 25 association signals [8]. Three of these signals were novel, mapping to *GATA3*, *MUC5AC* and *KIAA1109*, but have since been identified in a large multi-ancestry meta-analysis of all-comer asthma [8, 10].

In this study, we hypothesised that linked electronic health records (EHRs) in UK Biobank would enable us to identify a more severe form of asthma characterised by high treatment burden and/or worse outcomes, which may have specific genetic risk factors. We performed a discovery GWAS and tested reproducibility of the associated signals in four independent cohorts. We then performed fine-mapping and variant-to-gene mapping of replicated loci to identify candidate causal genes that could be prioritised for subsequent studies.

## Material and methods

### Asthma definition in UK Biobank

**FIGURE 1:**
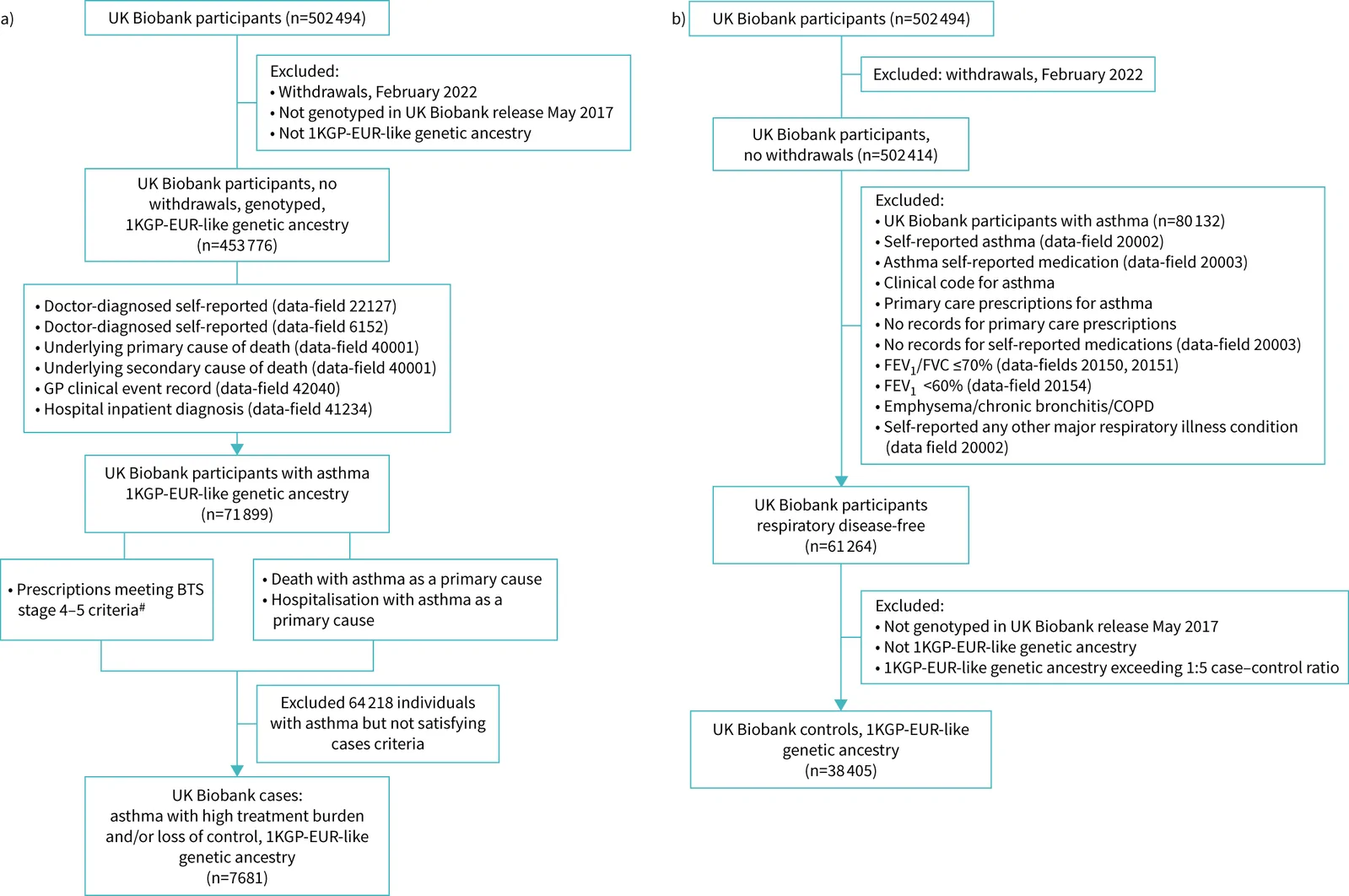
Phenotype design to identify asthma with high treatment burden and/or worse outcomes in UK Biobank. a) Asthma and cases definition; b) controls definition. 1KGP: 1000 Genomes Project; EUR: European; GP: general practice; BTS: British Thoracic Society; FEV_1_: forced expiratory volume in 1 s; FVC: forced vital capacity. ^#^: if individuals were selected as cases based purely on prescription data, we excluded those with comorbidities COPD, emphysema and chronic bronchitis, for which they may be prescribed similar medications.

UK Biobank is a resource integrating information on genetics, lifestyle and health records for individuals aged 40–69 years [11]. Participants with asthma were identified as having self-reported doctor-diagnosed asthma or diagnostic codes for asthma in EHRs (figure 1a). Only individuals whose genotyping data clustered with the European (EUR) reference populations from the 1000 Genomes Project (1KGP) samples were included (hereafter referred to as 1KGP-EUR-like [12]); more details have been reported elsewhere previously [13]. Within the asthma group, severe asthma cases with “high treatment burden and/or worse outcomes” were selected based on either 1) prescriptions characteristic of stages 4 and 5 of the British Thoracic Society (BTS) 2019 guidelines [1] (supplementary figure S1); or 2) hospitalisation and/or death due to asthma (figure 1a). Controls were defined as respiratory disease-free individuals, with forced expiratory volume in 1 s (FEV_1_)/forced vital capacity (FVC) ratio ≥70% and FEV_1_ >60% predicted; we selected five sex-matched controls for every case (figure 1b). Further details of case/control definitions are provided in the supplementary note, supplementary figure S2 and supplementary tables S1–S4.

### Genotype and imputation data

UK Biobank individuals were genotyped using the UK Biobank Axiom Array and the Axiom UK BiLEVE array [11]. Imputed data for 93M autosomal variants were available based on a combined reference panel from the Haplotype Reference Consortium panel [14], the UK10K [15] and 1000GP Phase3 [11, 16]. We filtered for variants with a minor allele frequency (MAF) ≥1%.

### Discovery GWAS

We performed the discovery GWAS using logistic regression implemented in REGENIE v2.2.4 [17] and including age at recruitment, age at recruitment squared, genetic sex and the first 10 ancestry-based principal components as covariates (supplementary material). Q–Q plots and Manhattan plots were generated using the R package “qqman” [18]. In order to identify any residual genomic inflation, we calculated the linkage disequilibrium score (LDSC) regression intercept using ldscv1.0.1 [19] and the linkage disequilibrium patterns of the European super-population of 1000GP [16]. For sentinel variant selection, we used a genome-wide suggestive threshold of p-value <5×10^−6^; we iteratively selected the sentinel variants for each genomic locus as the one with the lowest p-value in its surrounding region of 1 Mb (±500 Kb). To identify any additional independently associated variants, we performed conditional analyses in regions ±1 Mb from the sentinel variants using GCTA software (v1.90.2beta) [20] (chromosome six major histocompatibility (MHC) region was excluded). To investigate whether the associated signals were driven by differences in body mass index (BMI), smoking status or allergic factors between cases and controls, we conducted a sensitivity analysis (supplementary material).

### Replication

Replication of associated variants was performed by a meta-analysis of four cohorts of severe/moderate-to-severe asthma: 1) Resource for Genetic Epidemiology Research on Aging (GERA) [21]; 2) seq-GWAS with clinical trials participants with moderate-to-severe asthma [6]; 3) GASP study [8] and U-BIOPRED [22]; 4) Children's Respiratory and Environmental Workgroup (CREW) [23, 24]. More information on phenotype definition, genetic data and demographics in replication cohorts is available in the supplementary material and supplementary tables S5–S7. If a variant was not present in the replication cohorts, we selected a proxy based on highest linkage disequilibrium with the original sentinel (R^2^>0.8). The meta-analysis was run using METASOFT software with the random-effect model 2 [25] to account for differences in the underlying populations and case definitions and a Bonferroni-corrected p-value for the number of variants analysed.

### Comparison of effect sizes with all asthma case–control

To compare the effect estimates of the replicated signals between our refined case–control study population and the largest analysis to date of all asthma (GBMI) [10], we performed a correlation analysis across the replicated sentinel variants. We then extended this analysis to index variants of all signals identified by the GBMI study (supplementary note).

### “High treatment burden and/or worse outcomes” *versus* other asthma

As a secondary analysis, we performed a GWAS of our “high treatment burden and/or worse outcomes” cases *versus* other asthma. Other asthma was defined as no evidence of stages 4–5 medication and no evidence of exacerbation based on the definition used by Edris *et al*. [26] (supplementary figure S3). GWAS was conducted as in the discovery GWAS, but we additionally included age of asthma onset as a covariate.

### Fine-mapping

We used the Sum of Single Effects regression with summary statistics (SuSiE-RSS) [27] method to perform statistical fine-mapping on the replicated loci, as implemented in the R package susieR (v0.12.35). We used summary statistics from our discovery analysis with an in-sample linkage disequilibrium matrix as input. The MHC region was removed due to its complex linkage disequilibrium patterns. Genomic loci were defined as regions of 1 Mb centred on the replicated sentinel variant; if loci overlapped, they were merged and analysed as a single region. We only reported credible sets with a minimum of 90% confidence of including at least one causal variant (coverage parameter ≥0.90). Variants were annotated using Gencode [28, 29] as implemented in the FAVOR webtool [30].

### Variant to gene mapping

We used variants included in the fine-mapped credible sets to identify and prioritise associated genes. Genes were mapped to the credible set variants based on eight sources of orthogonal evidence: 1) nearest gene to the variant with the highest posterior inclusion probability; 2) functional annotation of credible set variants; 3) colocalisation with expression quantitative trait loci (eQTL); 4) look-up of protein quantitative trait loci (pQTL); 5) genes with highest Polygenic Priority Score; 6) nearby Mendelian disease genes; 7) nearby orthologue genes in mouse knockout phenotypes; 8) look-up in exome-wide association analyses (ExWAS) of “high treatment burden and/or worse outcomes” asthma for rare variants (supplementary material). We prioritised genes with three or more lines of evidence. We investigated the mapped genes using STRING v12.0 (April 2025) [31] for protein interaction and pathway enrichment analysis using data from seven different databases (biological process: Gene Ontology; local network cluster: STRING, Kyoto Encyclopedia of Genes and Genomes (KEGG) pathway, Reactome Pathway; tissue expression: TISSUES; disease-gene association: DISEASES; human phenotype: Monarch). Significant enrichment was defined using a false discovery rate <0.001.

## Results

### Case/control selection

We used EHRs in UK Biobank to identify individuals with high asthma treatment burden and/or worse outcomes (death or hospitalisation due to asthma) among participants with asthma. A total of 71 899 individuals met a broad definition for asthma, among which 7681 were identified as more severe asthma cases (“high treatment burden and/or worse outcomes”), while the remaining 64 218 individuals did not satisfy case criteria (figure 1a). We compared descriptive and clinical measures between the case group and the remaining asthma population and found that the cases were significantly enriched (p≤0.001) for women (64% *versus* 57%), higher BMI (29 *versus* 28 kg·m^−2^), and adult-onset asthma (59% *versus* 50%) (table 1). Prednisolone use was five times higher in cases than the rest of the asthma population (46.17% *versus* 8.66%). Cases were sex-matched with 38 405 respiratory disease-free controls of European ancestry (figure 1b).

**TABLE 1:** Descriptive analysis of asthma cases (“high treatment burden and/or worse outcomes”), individuals with asthma who did not meet case criteria, and controls

	Asthma, cases	Asthma, not cases	Controls^#^
**Cases**	7681	64 218	38 405
**Age years**	56.92±8.07	56.40±8.18	57.0±7.87
**Sex**			
Female	4941 (64.33)	36 367 (56.63)***	24 705 (64.33)
Male	2740 (35.67)	27 851 (43.37)	13 700 (35.67)
**BMI kg·m^−2^**	28.95±5.78	27.63±4.45***	27.01±4.05***
**Lung function measure**			
FEV_1_ % predicted	83.14±16.98	86.37±15.88***	95.84±12.16***
FEV_1_/FVC	0.73±0.08	0.74±0.07***	0.78±0.04***
**Blood cell count ×10^9^ cells·L^−1^**			
Eosinophils	0.22±0.13	0.20±0.12***	0.14±0.08***
Neutrophils	4.58±1.39	4.31±1.27***	4.09±1.15***
**Smoking status**			
Smokers	3314 (43.14)	29 997 (46.71)***	15 731 (40.96)***
Nonsmokers	4196 (54.63)	33 225 (51.74)	22 067 (57.46)
Unknown	171 (2.23)	996 (1.55)	607 (1.58)
**Category onset**			
Adult (age ≥18 years)	4514 (58.77)	31 830 (49.57)***	NA
Childhood (age <18 years)	1539 (20.04)	13 362 (20.81)	NA
Unknown	1628 (21.19)	19 026 (29.63)	NA
**Primary care records available**			
Yes	5783 (75.29)	29.480 (45.91)	38 405 (100)
**Prednisolone use**			
Yes	3546 (46.17)	5564 (8.66)***	0
No	2237 (29.12)	23 916 (37.24)	38 405 (100)
Unknown	1898 (24.71)	34 738 (54.09)	0
**Hay fever/allergic rhinitis or eczema/atopic dermatitis**			
Yes	4879 (63.52)	35 744 (55.66)***	14 386 (37.46)***
No	2802 (36.48)	28 474 (44.34)	24 019 (62.54)

Data are presented as n, mean±sd or n (%). Details of descriptive factors are reported in the supplementary material. BMI: body mass index; FEV_1_: forced expiratory volume in 1 s; FVC: forced vital capacity; NA: not applicable. ^#^: matched to cases for sex. ***: p≤0.001 (Wilcoxon–Mann–Whitney test for continuous variables, Chi-squared test for categorical variables).

### Discovery GWAS

**FIGURE 2:**
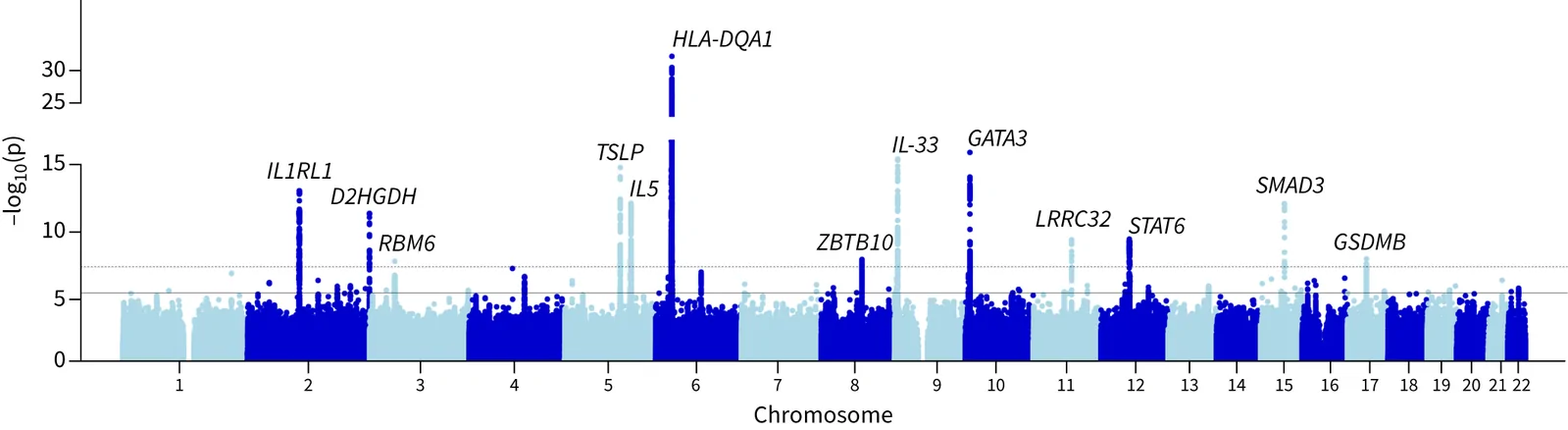
Manhattan plot of discovery genome-wide association study. Solid line is suggestive threshold (5×10^−6^); dashed line is genome-wide threshold (5×10^−8^). Nearest coding gene is shown for genome-wide significant loci.

We then investigated whether our refined severe asthma phenotype harboured new or known genetic risk loci by conducting a GWAS of 9 804 778 variants in 7681 cases and 38 405 respiratory disease-free controls; 11 917 variants showed suggestive association (p<5×10^−6^) and 7198 variants met genome-wide significance (p≤5×10^−8^) (figure 2). The QQ-plot showed an expected distribution of p-values and the LDSC regression intercept was 1.02, suggesting little systematic bias due to population stratification (supplementary figure S4). Sentinel selection and conditional analyses identified 68 independently associated single nucleotide polymorphisms (SNPs) at suggestive significance, 17 (25%) of which had not been associated with asthma before. We ran a sensitivity analysis adjusting for BMI and ever-smoking and with allergy-free cases and controls, which all showed the same direction of effect and comparable genetic effect sizes to the discovery analysis. This indicates that the associations were not driven by differences in BMI, smoking status or allergic status between cases and controls (supplementary material, supplementary figures S5–S7). The majority of the sentinel variants had been previously reported for association with asthma (supplementary table S8).

### Replication

To replicate our findings, we tested the 68 associated signals for replication in 12 152 cases and 32 316 controls from four independent studies of moderate-to-severe asthma (GERA [21]; seq-GWAS moderate-to-severe asthma [6]; GASP and U-BIOPRED [22]; CREW [23, 24]). We were able to test 62 out of the 68 signals, as six variants were not present in the data and did not have any proxies (supplementary table S9). Three of these variants were low-frequency single nucleotide variants (MAF<0.05) and may therefore be absent from reference panels or not well imputed in other cohorts. Two are indels adjacent to homopolymer runs and are therefore not consistently labelled in other datasets. One is a known single nucleotide variant with MAF=0.10 and may be absent from reference panels. 21 signals were replicated at a Bonferroni-corrected p-value threshold (8.06×10^−4^) (table 2 and supplementary figure S8–S9), all of which had previous evidence for association with asthma. To assess whether our replicated signals show similar or different effect sizes in all-comer asthma, we compared our effect estimates with those of the Global Biobank Meta-analysis Initiative (GBMI) European meta-analysis [10]. The signals showed the same direction of effect in GBMI, but our discovery study showed larger effect sizes for all signals (supplementary figure S10). We extended our effect size comparison to all the GBMI asthma signals and found an increase in genetic effect size (average 34%) in our discovery GWAS compared to the GBMI all-comer asthma GWAS (supplementary figure S11).

**TABLE 2:** Replicated associations of variants with asthma characterised by high-intensity treatment and/or worse outcomes

SNP	Position (b38)	Effect allele	Non-effect allele	Effect allele frequency	UK Biobank OR (95% CI)	UK Biobank p-value	Replication OR (95% CI)	Replication p-value	Nearest coding gene
**rs12470864**	chr2:102309902	A	G	0.383	1.15 (1.11–1.19)	7.55×10^−14^	1.13 (1.07–1.2)	9.94×10^−13^	*IL1RL1*
**rs6761047**	chr2:241753443	T	C	0.246	0.86 (0.83–0.9)	4.18×10^−12^	0.86 (0.75–0.98)	2.04×10^−08^	*D2HGDH*
**rs35570272**	chr3:33006170	T	G	0.397	1.09 (1.05–1.13)	3.16×10^−06^	1.07 (1.01–1.14)	1.23×10^−04^	*GLB1*
**rs1837253**	chr5:111066174	C	T	0.734	1.18 (1.13–1.23)	1.25×10^−15^	1.24 (1.11–1.38)	1.08×10^−20^	*TSLP*
**rs3806932**	chr5:111069977	G	A	0.441	0.91 (0.87–0.94)	1.03×10^−07^	0.90 (0.84–0.97)	3.99×10^−12^	*TSLP*
**rs2188962**	chr5:132435113	T	C	0.412	0.88 (0.85–0.91)	6.88×10^−13^	0.91 (0.83–0.99)	2.54×10^−07^	*SLC22A5*
**rs848**	chr5:132660808	C	A	0.806	0.85 (0.82–0.89)	3.96×10^−12^	0.85 (0.77–0.93)	6.36×10^−17^	*IL13*
**rs9271365**	chr6:32619017	G	T	0.426	1.24 (1.20–1.290)	9.20×10^−33^	1.11 (0.96–1.28)	4.60×10^−06^	*HLA-DQA1*
**rs148639908**	chr6:90253895	AT	A	0.344	0.90 (0.87–0.94)	1.30×10^−07^	0.94 (0.83–1.05)	3.19×10^−04^	*BACH2*
**rs7824394**	chr8:80380364	C	A	0.635	0.90 (0.87–0.93)	1.39×10^−08^	0.94 (0.9–0.97)	1.08×10^−04^	*ZBTB10*
**rs992969**	chr9:6209697	G	A	0.747	0.84 (0.81–0.88)	2.83×10^−16^	0.85 (0.82–0.88)	1.39×10^−17^	*IL33*
**rs201499805**	chr10:9000781	C	CT	0.314	0.84 (0.8–0.87)	8.79×10^−17^	0.78 (0.62–0.98)	2.95×10^−09^	*GATA3*
**rs1444789**	chr10:9022398	C	T	0.191	1.19 (1.14–1.24)	1.17×10^−14^	1.13 (1.08–1.18)	5.69×10^−09^	*GATA3*
**rs10160518**	chr11:76585627	G	A	0.507	1.12 (1.08–1.16)	4.29×10^−10^	1.15 (1.09–1.21)	5.98×10^−15^	*LRRC32*
**rs73107993**	chr12:47802090	T	C	0.240	0.90 (0.86–0.94)	1.10×10^−06^	0.90 (0.85–0.96)	2.57×10^−06^	*HDAC7*
**rs705705**	chr12:56041720	C	G	0.332	1.13 (1.09–1.17)	3.85×10^−10^	1.07 (1.03–1.12)	1.19×10^−04^	*RPS26*
**rs3024971**	chr12:57099944	G	T	0.104	0.83 (0.78–0.88)	5.92×10^−10^	0.87 (0.82–0.91)	3.21×10^−07^	*STAT6*
**rs11071559**	chr15:60777789	T	C	0.140	0.88 (0.83–0.93)	3.82×10^−06^	0.92 (0.87–0.96)	6.21×10^−04^	*RORA*
**rs17293632**	chr15:67150258	T	C	0.230	1.16 (1.12–1.21)	7.38×10^−13^	1.13 (1.09–1.17)	1.09×10^−10^	*SMAD3*
**rs3024619**	chr16:27353485	A	G	0.351	1.10 (1.06–1.14)	1.30×10^−06^	1.09 (1.03–1.15)	7.78×10^−06^	*IL4R*
**17:38073838_CCG_C**	chr17:39917585	C	CCG	0.423	1.12 (1.08–1.16)	1.25×10^−08^	1.20 (1.08–1.33)	4.97×10^−12^	*GSDMB*

SNP: single nucleotide polymorphism.

### Fine-mapping

15 credible sets passed the 90% confidence level, including 344 putative causal variants (table 3, supplementary table S10, supplementary figure S12). The majority of the variants were annotated as intronic (44.76%), followed by noncoding RNA intronic (35.91%) and intergenic (13.95%); only two variants were exonic (0.58%): a synonymous change in *IL1RL1* (rs1420101, L227L), and a nonsynonymous change in *D2HGDH* (rs1106639, V338I).

**TABLE 3:** Fine-mapping of replicated signals that identified a credible set (CS)

Replicated sentinel SNP	Locus	Number of SNPs in CS	Nearest coding gene^#^	Highest PIP variant
rs12470864	Chr 2 q12.1	9	*IL1RL1*	rs12470864
rs6761047	Chr 2 q37.3	6	*D2HGDH*	rs6761047
rs1837253; rs3806932	Chr 5 q22.1	1 (CS 1)	*TSLP*	rs1837253
		41 (CS 2)		rs10455025
rs2188962; rs848	Chr5 q31.3	67 (CS 1)	*IL5/C5orf56*	rs1986009
		6 (CS 2)		rs2070729
rs148639908	Chr 6 q15	56	*BACH2*	rs148639908
rs7824394	Chr 8 q21.13	88	*ZBTB10*	rs7824394
rs992969	Chr 9 q24.1	6	*IL33*	rs992969
rs201499805; rs1444789	Chr 10 p14	3 (CS 1)	*GATA3*	rs12413578
		15 (CS 2)		rs1444789
rs10160518	Chr 11 q13.5	9	*EMSY*	rs10160518
rs705705	Chr 12 q13.2	30	*RPS26*	rs705705
rs3024971	Chr 12 q13.3	2	*STAT6*	rs3024971
rs17293632	Chr 15 q23	5	*SMAD3*	rs17293632
Total		344		

15 credible sets (coverage ≥0.9) were identified, representing 12 genomic regions (three regions included two credible sets). SNP: single nucleotide polymorphism; PIP: posterior inclusion probability. ^#^: nearest coding gene is relative to the highest PIP variant.

### Variant-to-gene mapping

**FIGURE 3:**
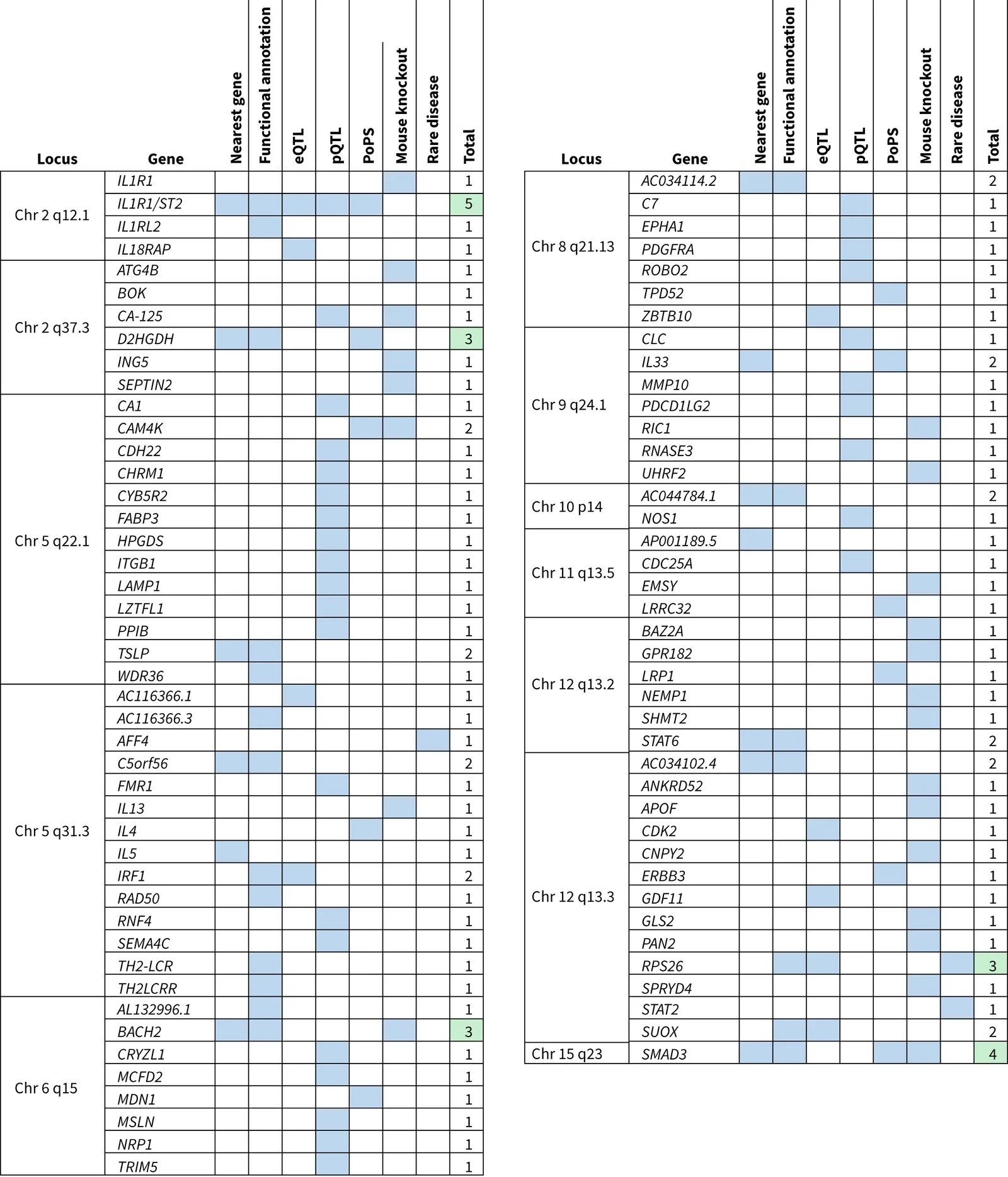
Locus–gene evidence matrix. Light blue cells represent evidence for the mapping between the locus and the gene. Genes with three or more lines of evidence are highlighted in green. Rare variant analysis did not highlight any mapped genes, and therefore it is not displayed. eQTL: expression quantitative trait loci; pQTL: protein quantitative trait loci; PoPS: Polygenic Priority Score.

We used eight lines of evidence to map genes to all of the variants included in the 15 credible sets defined earlier. We identified a total of 85 genes, among which five were supported by at least three independent criteria: *BACH2*, *D2HGDH*, *IL1RL1*, *RPS26*, *SMAD3* (figure 3, supplementary tables S11–S20). The pQTL analyses retrieved the highest number of candidate causal genes (30), while no genes were found from the look-up in ExWAS for rare variants. The rare variant analyses highlighted two genes, *TTK* and *INTS1*, outside the investigated genomic regions (supplementary tables S18–S19).

To identify enriched biological pathways and processes among mapped genes, we interrogated STRING. Both immune-related pathways and diseases were enriched among our genes, which included several key molecules of type 2 immunity, such as interleukin (IL)4–5-13-33, thymic stromal lymphopoietin (TSLP) and signal transducer and activator of transcription (STAT)6 (supplementary table S21, supplementary figure S13). In particular, the KEGG database highlighted four pathways: “cytokine–cytokine receptor interaction”, “inflammatory bowel disease”, “JAK-STAT signalling pathway” and “asthma”. Gene Ontology Biological Process highlighted the “immune response pathway” and Reactome showed two pathways, “Signalling by Interleukins” and “Cytokine Signaling in Immune system”. The TISSUE database showed enrichment for proteins expressed in asthma-related cells including eosinophils, basophils, granulocyte, mast cells, and also in nasal lavage fluid.

### “High treatment burden and/or worse outcomes” *versus* other asthma

In a secondary analysis of our cases *versus* other asthma with no evidence of stage 4–5 medication or exacerbations, we identified two signals (rs5004091: OR=1.13 (1.08–1.18), p=3.54×10^−8^; rs71387195: OR=1.27 (1.17–1.38), p=3.16×10^−8^) (supplementary figures S14–15). The rs5004091 variant on chromosome 1 was suggestively significant in our primary analysis, but did not replicate. The rs71387195 signal on chromosome 16 signal is novel and predicted to alter splicing of the *KIFC3* gene according to Genotype-Tissue Expression. This result should be treated with caution until it has been replicated by an independent study.

## Discussion

The identification of cases of “severe asthma” for genetic studies in large population-based cohorts can be challenging due to limited clinical data, and different studies have employed different case selection criteria. We identified an opportunity to use UK Biobank EHR to better define a more severe asthma phenotype at scale and use this case population to test the hypothesis that there may be specific genetic variants associated with this subtype. Selecting individuals with high treatment burden and/or worse outcomes, we identified a case population that significantly differs from other individuals with asthma in UK Biobank: cases were enriched for women, adult-onset asthma and those who had higher BMI. This is the first study to use a manually curated list of primary care prescriptions, death records, and hospitalisation data to define severe asthma in a biobank for genomic analyses.

We robustly replicated 21 previously described asthma susceptibility signals; however, we did not identify any signals unique to this newly defined asthma population. Nevertheless, we did observe that our genetic effect sizes were stronger in magnitude with respect to the GBMI study, suggesting an enrichment for genetically-driven asthma in our cases. Alternatively, environmental exposures among more severe cases could impact effect size due to genotype–environment interactions [32]. It is also possible that the effect sizes were larger in our study due to our robust definition of asthma, while cases in the GBMI study are more likely to be misclassified as having asthma. Finally, we defined credible sets of putative causal variants using Bayesian fine-mapping and applied a bespoke variant-to-gene mapping procedure resulting in 85 candidate causal genes, five of which were implicated by at least three lines of evidence (*BACH2*, *D2HGDH*, *IL1RL1*, *RPS26*, *SMAD3*). These findings advance our understanding of the genetic basis of more severe types of asthma and suggest that there is not a distinct set of genetic variants driving this phenotype. Other possible drivers of severity include burden of genetic risk factors, epigenetic changes and environmental risk factors.

The 85 mapped genes showed enrichment in essential pathways and cell types known to play a role in asthma, such as cytokine signalling and eosinophils. Five fine-mapped loci highlighted known drug targets for asthma or targets that are in clinical development. For example, tezepelumab and mepolizumab/benralizumab/reslizumab target *TSLP* and *IL5*, respectively [33–35] and drugs targeting *IL1RL1(ST2)* and *IL33* are under development for asthma and other respiratory and immunological conditions [36]. Additionally, *SMAD3 is* involved in airway remodelling [37] and it was previously identified as a druggable target gene for asthma [38]. This raises the exciting prospect that other identified loci may harbour potentially promising therapeutic targets, in particular, *BACH2*, *D2HGDH* and *RPS26*, which were supported by at least three lines of evidence but have not yet been targeted for asthma therapies.

BTB Domain And CNC Homolog 2 (*BACH2*) encodes a transcriptional regulatory protein that can act as repressor or activator and it has a role in immune response, being a key regulator of T-cell function and B-cell maturation [39]. In a previous differential expression analysis in blood from severe asthma patients, *BACH2* mRNA expression was lower in severe asthma patients when compared to control and to mild-to-moderate asthma [40]. Our signal, rs148639908, is an intronic variant mapped to *BACH2.* It is only nominally associated with *BACH2* expression in oesophagus muscularis and skin, and the risk allele was associated with increased, not decreased expression (p=0.005; supplementary table S22). Colocalisation in whole blood suggested that the GWAS signal and the eQTL signal are statistically independent. Further analyses of *BACH2* expression in tissues of interest might help to disentangle the regulation of this gene in the context of asthma risk.

D-2-hydroxyglutarate dehydrogenase (*D2HGDH*) encodes a mitochondrial enzyme involved in the conversion of D-2-hydroxyglutarate to 2-ketoglutarate [41]. Rare mutations in *D2HGDH* causes D-2-hydroxyglutaric aciduria type 1 which is a rare recessive disease characterised by developmental delays, epilepsy, hypotonia and dysmorphic features [42]. The asthma signal rs1106639 was significantly associated with decreased expression of *D2HGDH* in 10 tissues in GTExv8 (supplementary table S22); the colocalisation did not highlight a shared signal between eQTL and GWAS, in line with the results of Zhu *et al.* [43]. Nevertheless, the credible set variants were shown to be included in candidate regulatory regions upstream of *D2HGDH*, suggesting that these variants could be good candidates for future functional experiments [44].

*RPS26* encodes the ribosomal protein S26, which plays a role in the 40S ribosomal subunit and the synthesis of other proteins [45]. The asthma-risk allele of rs705705 was associated with increased levels of *RPS26* through its proxy rs705704 (R^2^=1) (supplementary table S22), and the GWAS and eQTL signals showed colocalisation in seven tissues including lung (supplementary table S13). Mutations in *RPS26* and other ribosomal-protein genes have been linked to Diamond–Blackfan anaemia, a rare disease that is treated with long-term courses of corticosteroid [46]. *RPS26* is targeted by existing medication ataluren, which has been studied in clinical trials for respiratory conditions (Open Targets Platform [47], accessed on 25 June 2024) and its potential for alleviating asthma will need further investigation. The sentinel variant rs705705 is located only 198 base pairs away from the transcription start site of *RPS26*, which makes this variant a good candidate for functional experiments [48].

While reproducible loci from this and previous GWAS of moderate-to-severe have been found associated with all-comer asthma, GWAS of asthma exacerbations have highlighted some novel loci [26, 49–52]. We looked these loci up in our discovery GWAS and found no statistical associations, except with rs56151658 (p=5.50×10^−19^), a SNP upstream of *HLA-DQB1* and weakly correlated with our sentinel rs 9271365, which had previously been associated with asthma hospitalisations [52], and rs1800925 (p=4.95×10^−7^), a SNP upstream of *IL13* and weakly correlated with our sentinel rs848, which had been associated with asthma exacerbations in children [50].

A key strength of this study was the use of primary prescription data and the implementation of a manually curated list of asthma drugs and drug combinations to identify individuals with high treatment burden as a proxy for asthma with clinical unmet need. However, there is no systematic data on symptom control in UK Biobank and prescription records do not guarantee treatment adherence, meaning precise alignment to the clinical definition of severe asthma was not possible. Furthermore, clinical definitions are dynamic and they are influenced by the periodic updates of national and international guidelines, such as BTS [1] or Global Initiative for Asthma [53]. Phenotype definition in biobanks may benefit from a systematic coding for primary care prescriptions; for example, including dosages to distinguish between high/medium/low-dose inhaled corticosteroid prescriptions using the number of puffs per day. We also made the decision to collapse all the prescriptions for an individual into one unique virtual time point; a possible extension of our study would be to characterise the long-term course of the treatment to validate the presence of a severe condition and avoid misclassification due to episodic worsening of asthma in mild/moderate patients. Finally, we acknowledge that our study included only individuals of 1KGP-EUR-like genetic ancestry, and we encourage future collaborations with diverse cohorts.

To conclude, our analyses highlighted 21 reproducible loci for asthma with high treatment burden and/or worse outcomes, which were fine-mapped and linked to multiple genes. Among the prioritised genes, we recommend further investigation of *RPS26*, *BACH2* and *D2HGDH* as therapeutic targets for asthma.

## Data Availability

Summary statistics of the discovery genome-wide association study (GWAS) have been uploaded to GWAS catalogue and will be available after publication. UK Biobank individual-level data are available to approved researchers by registering at https://www.ukbiobank.ac.uk/enable-your-research/register. Genotype tissue expression data are available at https://www.gtexportal.org/home/aboutAdultGtex. eQTLgen Blood is available at https://www.eqtlgen.org/phase1.html. The Lung-eQTL consortium is available at https://doi.org/10.1371/journal.pgen.1003029. deCODE plasma protein quantitative trait loci (pQTL) are available at https://pubmed.ncbi.nlm.nih.gov/34857953/. SCALLOP pQTL is available at https://pubmed.ncbi.nlm.nih.gov/33067605/. UK Biobank pQTL is available at: https://www.nature.com/articles/s41586-023-06592-6. All analyses were performed using Bash, R or Python within the High Performance Computing SPECTRE2 and ALICE service at the University of Leicester. Code is available at https://github.com/legenepi/UKBiobank_asthma_medication, https://github.com/legenepi/UKBiobank_asthmaMeds_stratification, https://github.com/legenepi/REGENIE_assoc_severe_asthma.git, https://github.com/legenepi/Post_GWAS, https://github.com/legenepi/Fine_mapping_severe_asthma, https://github.com/legenepi/VarToGene, https://github.com/legenepi/tmp_SA_manuscript and https://github.com/legenepi/severe_asthma_manuscript_revision.

## Supplementary material

**Please note:** supplementary material is not edited by the Editorial Office, and is uploaded as it has been supplied by the author. Supplementary material 00327-2026.SUPPLEMENT Supplementary tables 00327-2026.SUPPLEMENT
