# Supplementary material for: Genome-wide association study of asthma with high treatment burden and/or worse outcomes defined using electronic healthcare data in UK Biobank
Source: ERJ Open Res. 2026 Sep 1;12(5):00327-2026. doi: 10.1183/23120541.00327-2026 (PMC13531276; doi:10.1183/23120541.00327-2026)
Supplement: Supplementary file 1 [file 00327-2026.SUPPLEMENT.pdf]

Supplementary Information Notes and Figures

Contents

Asthma definition in UK Biobank 2
Using prescription records to identify asthma cases with high treatment burden 3
COPD/emphysema/chronic bronchitis definition 3
Descriptive analysis data 3
REGENIE GWAS 3
BMI sensitivity analysis 4
Smoking status sensitivity analysis 4
Allergy free sensitivity analysis 4
Comorbidity free sensitivity analysis 5
Replication cohorts 5
Genetic effect size comparison for known asthma loci 6
Variant-to-gene mapping 6
Figure S1 8
Figure S2 9
Figure S3 10
Figure S4 11
Figure S5 12
Figure S6 13
Figure S7 14
Figure S8 15
Figure S9 26
Figure S10 28
Figure S11 29
Figure S12 31
Figure S13 39
Figure S14 40
Figure S15 41
Figure S16 42
References 43

Asthma definition in UK Biobank

UK Biobank is one of the biggest resources integrating information on genetics, lifestyle, and health records [1]. Participants with asthma were identified as having any of the following: self-reported doctor-diagnosed asthma (Data-Fields 6152 or 22127; value '8' and '1' respectively); readv2 or readCTV3 codes for asthma in general practice (GP) clinical records (Data-Fields 42040); an ICD-9 or ICD-10 code for asthma in hospital inpatient diagnoses (Data-Field 41234; ICD-9 and ICD-10); an ICD-10 code for asthma in death records (Data-Fields 40001 or 40002) (Figure 1A). Diagnostic and clinical codes (ICD-9/ICD-10, readv2/readCTV3) were derived from the UK Biobank Resources 594 and 592. Only individuals whose genotyping data clustered with the European 1000 Genomes samples were included.

Case selection

Individuals with asthma and who met any of the following three criteria were selected as cases:

1) Prescriptions for medication within primary care prescription data that are characteristic of stages 4 and 5 of the British Thoracic Society (BTS) 2019 guidelines[2] including combinations of either: i) high-dose inhaled corticosteroids (ICS) with any other medications; ii) low or high dose ICS with long-acting beta agonist or long-acting muscarinic antagonist (Supplementary Information, Figure S2, Table S1-S2). Participants with a diagnosis for chronic obstructive pulmonary disease (COPD), emphysema, and/or chronic bronchitis were excluded to ensure that the prescription was made for asthma and no other comorbidities (Figure S1 and Supplementary Information).
2) Hospital in-patient record with an ICD-9 or ICD-10 code for asthma as the primary cause, regardless of comorbidity status
3) Death record with an ICD-10 code for asthma as the primary cause, regardless of comorbidity status

Control selection

Controls were defined as individuals with self-reported medication and/or prescription data and without asthma, emphysema, chronic bronchitis or COPD diagnostic codes (Table S3-S4). In addition, controls were free from any other major respiratory condition (Data-field 20002, value '1111' for asthma and '1072/1466/1130/1131/1132/1133/1134/1135/1660/1139/1140/1141/1142/1143/1144/1467/1164/1168/1534/1559/1560/1561/1562/1563/1465' for other respiratory conditions). We also excluded individuals with forced expiratory volume and forced vital capacity ratio (FEV1/FVC) <= 70% (Data-Field 20150, 20151) and predicted percentage FEV1 < 60% (Data-Field 20154). We selected five sex-matched controls of European ancestry for every case (Figure 1B) as power calculations indicated that power begins to plateau at 4 controls per case and that there is very little extra gain in power with greater than 5 controls per case.

Using prescription records to identify asthma cases with high treatment burden

All prescriptions for individuals with asthma were extracted from the primary care records; if a drug name was absent, we mapped the readv2 codes to drug names using the 'read_v2_drugs_lkp' table from the UK Biobank Resource 592. This list of prescriptions was filtered for asthma-specific prescriptions and medications, excluding 'nasal spray/nasal/spray/cream' terms and 'MOMETASONE FUROATE' (Figure S1). We then divided all the asthma related medications into drug classes: ICS = inhaled corticosteroids; SABA = short-acting beta agonist; LABA = long-acting beta agonist; LAMA = long-acting muscarinic antagonist; LTRA = leukotriene receptor antagonist (Table S1-S2). We ascertained the combinations of the classes of drugs for each participant. Cases with high treatment burden were selected as those having drug combinations corresponding to stages 4 and 5 as per the British Thoracic Society (BTS) 2019 guidelines [2] (Table S2):

- high dose ICS* + any other medication (except SABA or sodium cromoglicate);
- (any dose of ICS + LABA) + LTRA/LAMA/theophylline/prednisolone;
- ICS + LAMA +/- any other medication

As UK Biobank prescription records do not specify treatment dosages, we used prescription of a high-dose inhaler as a surrogate (listed in the Respiratory Dashboard Appendix 2 ('High Dose ICS Items' table) of the NHS Business Services Authority (BSA) [3].

COPD/emphysema/chronic bronchitis definition

An individual was identified as having emphysema/chronic bronchitis if they had any of self-reported doctor-diagnosed emphysema or chronic bronchitis (Data-Fields 6152, 22128, or 22129; value '6', '1' and '1' respectively); readv2 or readCTV3 codes for emphysema/chronic bronchitis in general practice (GP) clinical records (Data-Fields 42040); an ICD-9 or ICD-10 code for emphysema/chronic bronchitis in hospital inpatient records (Data-Field 41234; ICD-9 and ICD-10); an ICD-10 code for emphysema/chronic bronchitis in death records (Data-Fields 40001 or 40002). ICD-10 codes J40/J41/ J42/ J43 were used to retrieve clinical codes readv2 and readCTV3 within UK Biobank Data-Coding 1834 and Data-Coding 1835 respectively (Table S3). To find participants with COPD, we used a list of diagnosis and clinical codes as obtained by an automated analysis made with the in-house software DeepPheWAS [4] (Table S4) for Data-Field 40002/40003/42040/41234, with information from self-reported diagnosis (Data-Field 20002). The overall workflow is illustrated in Figure S2.

Descriptive analysis data

We compared asthma cases against asthma individuals not qualifying for case definition, and asthma cases against respiratory disease-free controls. We used BMI as reported in UK Biobank Data-Field 21001; lung function measures as previously defined in Shrine et al. 2019 [5]; smoking status as previously defined in Piga et al. 2022 [6] . We defined category onset based on Data-Field 22147 and 3786; we defined adult-onset asthma as age at onset equal or greater than 18. Baseline eosinophil count was reported in Data-Field 30150; baseline neutrophil count was reported in Data-Field 30140. Quantitative variables were compared with Wilcox Mann-Whitney tests; categorical variables were compared with chi-square tests. Analyses were performed in R 4.3.1.

REGENIE GWAS

REGENIE is a machine-learning approach based on a two-step analysis and has shown better performance in terms of time and memory with respect to other similar methods [7]. In step 1, a set

of genotyped and good quality SNPs is used to create a whole genome regression model for the trait and obtains leave-one-chromosome-out (LOCO) predictors. In step 2, LOCO predictors are included as fixed covariates into the linear (or logistic) regression model. In this way, REGENIE can handle related individuals. For binary traits, REGENIE implements a score test, but it can also use a time efficient approximate Firth logistic regression test which reduces biases derived for the maximum-likelihood estimates, and it gives good quality Type 1 errors, SNP estimates and standard errors. Similar to the score test, the approximate Firth test follows a χ^2 distribution with one degree of freedom. We used the default p-value threshold (0.05) below which the Firth test is applied.

For REGENIE step 1, we used directly genotyped variants passing quality control [7]. Individuals with >10% missing genotypes were excluded. For REGENIE step 2, we included imputed variants with minor allele frequency (MAF) >= 1%, minor allele count (MAC) >= 10 and INFO imputation score >= 0.3. Power calculations were performed with the online tool GAS Power Calculator [8] confirming statistical power > 80% to detect a genotype relative risk of at least 1.56 for variants with MAF > 0.01 and a genotype relative risk of at least > 1.12 for variants with MAF > 0.2.

BMI sensitivity analysis

As our “high treatment burden and/or worse outcomes” cases and our non-asthma controls differed by BMI, we repeated association testing of suggestively significant loci with BMI as a continuous covariate. As shown in Figure S5, the effect sizes did not differ between the discovery GWAS and the sensitivity analysis, suggesting that these associations are not driven by differences in BMI.

Smoking status sensitivity analysis

As there were more smokers amongst our “high treatment burden and/or worse outcomes” cases compared to controls, we repeated association testing of suggestively significant loci with a binary covariate indicating whether an individual had ever-smoked. As shown in Figure S6, the effect sizes did not differ between the discovery GWAS and the sensitivity analysis, suggesting that these associations are not driven by differences in smoking status.

Allergy free sensitivity analysis

In order to investigate if the associated signals were driven by allergy co-morbidities present in the asthma cases, we conducted a sensitivity analysis excluding individuals with allergic conditions. To define allergic phenotypes, we used self-reported doctor-diagnosed hay fever, allergic rhinitis or eczema (UK Biobank Data-Field 6152 with value ‘9’); non-cancer illness codes from nurse’s interview (Data-Field 20002); general practice clinical event records (Data-Field 42040; readv2, readCTV3); hospital inpatient diagnoses (Data-Field 41234; ICD-9 and ICD-10); and underlying cause of death (Data-Field 40001, 40002; ICD-10). For values in Data-Field 20002 and diagnosis codes ICD-9 and ICD-10, we used the list provided by Cameron-Christie et al. for their allergic phenotype definition in UK Biobank as reported in their Supplementary Material Section S1-C [9]. Clinical codes readv2 and readCTV3 for allergic diseases were retrieved from UK Biobank Data-Coding 1834 and Data-Coding 1835 using the ICD-10 codes. Excluding individuals with allergic conditions, we obtained 2,802 cases and 24,019 controls for the sensitivity analysis; the 67 sentinel variants showed consistent direction of effect and similar effect size to the discovery analysis indicating that the associations were not driven by the relationship between asthma and allergies (Figure S7).

Comorbidity free sensitivity analysis

1,488 (19%) of our cases with an asthma diagnosis as the primary cause of death and/or hospitalisation also have self-reported or diagnostic codes for comorbidities elsewhere in their records. We performed a sensitivity analysis with 6,193 cases (without comorbidity) and 38,405 respiratory disease-free controls (Supplementary Figure 16). This showed that our signals had a similar magnitude and direction of effect on asthma compared to the original GWAS.

Replication cohorts

We here report a brief description of each replication cohort. For further details on the phenotype definition, genetic data and case/control demographics see Supplementary Table S5-S7.

<u>Chang et al.</u>

The seq-GWAS study by Chang et al. included cases with moderate-to-severe asthma taking part in three clinical trials for omalizumab, five clinical trials for lebrikizumab, the TENOR II observation study and the BOBCAT biomarker study. Links to the inclusion/exclusion criteria for each of these studies can be found in the original paper [10]. Summary statistics were provided by the authors Single variant analyses were carried out using PLINK v1.9 and assuming an additive genetic effect model with genetic sex, and the top five PCs as covariates [10].

<u>GERA</u>

Genetic Epidemiology Research on Aging (GERA) cases were defined as individuals with self-reported and/or doctor-diagnosed asthma and either prescriptions indicative of BTS stage 4-5 medication or a history of exacerbations or both (Table SX'GWAS meta-analysis cohort description'). Association analyses were performed in 6,598 cases and 16,372 controls using PLINK v2 with covariates: first 5 PCs, sex, age (mean centered, divided by standard deviation(age)), age (mean centered, divided by standard deviation(age)) squared.

<u>GASP/U-BIOPRED/UK Biobank</u>

GASP and U-BIOPRED are two clinical cohorts. The participants were previously identified with moderate-to-severe asthma using clinical records for prescriptions within stage 3 to 5 of the BTS 2014 guidelines as previously described in the moderate-to-severe asthma GWAS {Shrine, 2019 #58}. For this study, only severe asthma cases were selected from GASP and UBIOPRED, and controls were selected from U-BIOPRED and UK Biobank. For controls from UK Biobank, we included 1KGP-EUR-like genetic ancestry controls that were not used in the discovery analysis. Controls were sex-matched with the cases with a ratio of 1:5 case-controls. The dataset included 2,083 cases defined as moderate severe asthma using BTS 3-5 criteria (Shrine et al 2019) and 10,415 controls. We re-imputed the genetic data for this cohort using the TopMed panel {Taliun, 2021 #120} and imputation server {Das, 2016 #121}. For the GWAS, we used REGENIE v3.2.5 with the same QC filters and models for step 1 and step 2 as described for the discovery analysis (Supplementary Material 'REGENIE GWAS'). A total of 8,901,272 variants were tested for 2,083 cases and 10,415 controls.

<u>CREW</u>

CREW stands for 'Children's Respiratory and Environmental Workgroup'. CREW includes children with asthma to investigate asthma phenotypes and endotypes[11]. A total of 290 severe asthma cases, with childhood onset asthma, were selected, with health care provider-diagnosed asthma and ≥2 reports of systemic corticosteroids use [12] during the study period - see Gern et al. 2019 for details[11]. 1,939 controls without physician-diagnosed asthma were selected. A logistic regression

was used to test the association of individual variants with severe asthma including age, age squared, sex, and the first 10 ancestry-based PCs as covariates.

Genetic effect size comparison for known asthma loci

To assess whether the effects of asthma risk loci were different in our subtype compared to all asthma, we compared the effect sizes of known-asthma loci using 128 index variants associated with asthma as identified by the Global Biobank Meta-analysis Initiative (GBMI) including 1KGP-EUR-like genetic ancestry individuals [13]. Welch's t-test was calculated for each variant to assess any significant dependency on the type of phenotype; Bonferroni correction for multiple testing was applied (p-value < 3.91×10^{-4}).

Variant-to-gene mapping

We used the credible sets from fine-mapping to identify and prioritise associated genes. Genes were mapped to the credible set variants based on eight different sources of orthogonal evidence: i) nearest gene; ii) functional annotation; iii) colocalization with expression quantitative trait loci (eQTL); iv) colocalization with protein quantitative trait loci (pQTL); v) Polygenic Priority Score (PoPS); vi) Mendelian disease evidence; vii) mouse knock-out phenotype; viii) associated rare variants from exome-wide association analyses (ExWAS). We prioritised genes when three or more approaches confirm the presence of association with a credible set variant. Here we describe each method in more details below.

Nearest gene mapping was retrieved by either using FAVOR annotation tool [14]; the nearest genes was identified with respect to the credible set variant (SNP with the highest posterior inclusion probability, PIP) for each genomic locus. We used FAVOR annotation tool [14] to annotate variant function; we included information for all credible set variants based on ten variant functional integrative scores with value > 15, a FANTOM5-predicted enhancer and/or promoter annotation, or a ClinVar annotation. The ten functional integrative scores were: CADD.phred, aPC.Protein.Function, aPC.Conservation, aPC.Epigenetics.Active, aPC.Epigenetics.Repressed, aPC.Epigenetics.Transcription, aPC.Local.Nucleotide.Diversity, aPC.Mutation.Density, aPC.Transcription.Factor, aPC.Mappability.

Colocalization of eQTL and GWAS signals was performed with coloc [15] and coloc.susie [16] software using cis-eQTL data (variants +/-1Mb from the gene's start site). Only eQTL with p-values <= 5×10^{-6} were included in this analysis and we used a colocalisation threshold of H_4 >= 0.90. eQTL data was retrieved from three databases: GTExV8 [17], eQTLGen [18], and Lung-eQTL consortium [19]. GTExV8 is a collection of eQTL data for variant-gene association in 49 tissues with at least 70 samples. We ran co-localisation for the following tissues: “Artery Aorta”, “Artery Coronary”, “Artery Tibial”, “Colon Sigmoid”, “Colon Transverse”, “Esophagus Gastroesophageal Junction”, “Esophagus Muscularis”, “Lung”, “Skin Not Sun Exposed Suprapubic”, “Skin Sun Exposed Lower leg”, ”Small Intestine Terminal Ileum”, “Stomach”. eQTLGen includes expression data for 16,989 genes from 37 blood tissue datasets in a total of 31,684 individuals. Lung-eQTL consortium is a collection of lung specimens from 1,111 individuals of European ancestry including 17,178 cis- and 593 trans- lung eQTLs. Raw data were adjusted to find significant p-values using eigenMT for LD structure and Benjamini Hochberg correction for multiple testing as described in Shrine et al. 2023[20].

To identify pQTLs amongst our credible set variants, we performed a look-up search in three sources: deCODE [21], SCALLOP [22], and UK Biobank [23]. deCODE has a free collection of plasma protein pQTL data for 4,907 proteins and 27.2 million tested variants. The level of significance was $1.8x10^{-9}$, including variants with MAF > 0.01% and imputation score > 0.9. Associated variants within 1Mb from the transcription start site of the gene were labelled as *cis*, otherwise as *trans*. SCALLOP (Systematic and Combined Analysis of Olink Proteins) combines measures from 90 circulating cardiovascular proteins in about 30,000 individuals from 13 cohorts. UK Biobank includes Olink data for 2,923 plasma proteins in 54,219 UK Biobank participants. We used association analysis of imputed variants (MAC>=5) with untransformed log2 fold protein levels, adjusting for olink batch, age, sex, genotyping array and the first ten genotyping-based PCs (as described in Shrine et al. 2023[20]). We used a Bonferroni p-value < $1.71x10^{-5}$ as significant association (0.05/2,923).

Polygenic Priority Score (PoPS) is an algorithm for gene prioritisation analysis. PoPS uses genome-wide signals from GWAS summary statistics to identify prioritised genes investigating public bulk and single-cell expression datasets, curated biological pathways, and predicted protein-protein interactions [24]. Detailed information of PoPS methodology is available in Weeks et al., 2023 [24] For each locus, we analysed all the genes within 500Kb from the variant with the highest PIP, and reported the top prioritised genes as ascertained by PoPS; if no prioritised genes, we enlarged the genomic window to 1MB.

For nearby gene in Mendelian diseases, we used the ORPHANET resource (https://www.orpha.net/). We checked whether any genes within 500Kb from the top causal variant for each fine-mapped locus was associated with rare diseases including the asthma Human Phenotype Ontology (HPO) term (HP:0002099) or any disease including asthma-related key terms ("asthma"/ "eosin"/ "immunodef"/ "cili"/ "autoimm" / "leukopenia" / "neutropenia" / "macroph").

For mouse knock-out analysis we used the International Mouse Phenotyping database (https://www.mousephenotype.org/) to identify human ortholog genes studied in mouse models in the context of respiratory, immune or muscle phenotypes (top Mouse Phenotype (MP) terms "respiratory system phenotype"/"immune system phenotype"/"muscle phenotype"). We selected relevant genes within 500Kb from the top causal variant for each fine mapped locus.

We performed single variant and gene-based exome-wide association analysis using REGENIE v3.1.1 on 7,413 asthma requiring high treatment burden and/or worse outcomes cases and 36,955 respiratory-free controls. Age at recruitment, age at recruitment squared, genetic sex, and the first 10 principal components were included as covariates in the models. For the gene-based analyses, we used three different masks as previously described [25]: Loss of function (LoF) only; LoF and missense variants (5/5) according to five annotation methods (SIFT[26], PolyPhen2 (HDIV) [27], PolyPhen2 (HVAR) [28], MutationTaster[29], and LRT[30]); LoF, missense(5/5), and missense in at least one of the five methods (>=1/5). Significantly associated variants were identified using a Bonferroni corrected p-value threshold for the number of markers analysed (0.05/N-markers: 0.05/1772264, p-value < $2.82x10^{-8}$). Genes mapped to rare variants (MAF < 0.01) within 500Kb of our credible set variants were included as likely causal gene for our -to-gene mapping pipeline.

Figure S1

Handling of prescription records to identify UK Biobank participants with asthma and high treatment burden. GP=general practitioner; COPD=chronic obstructive pulmonary disease; BTS=British Thoracic Society.

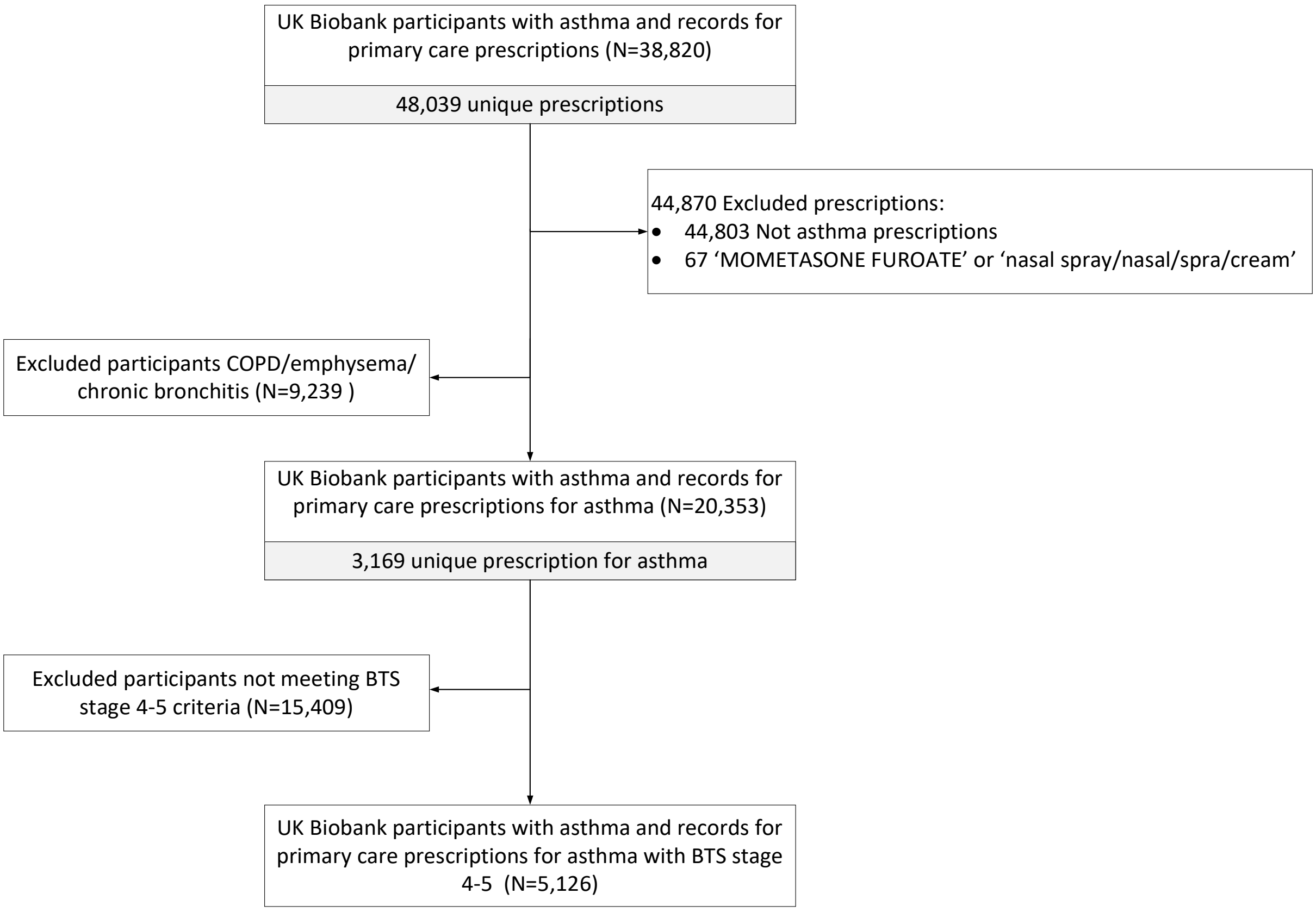

Figure S2

Phenotype definition for emphysema, chronic bronchitis and COPD. UK Biobank participants with a diagnosis for emphysema, chronic bronchitis and/or COPD; 38,873 individuals in total. GP=general practitioner; COPD=chronic obstructive pulmonary disease.

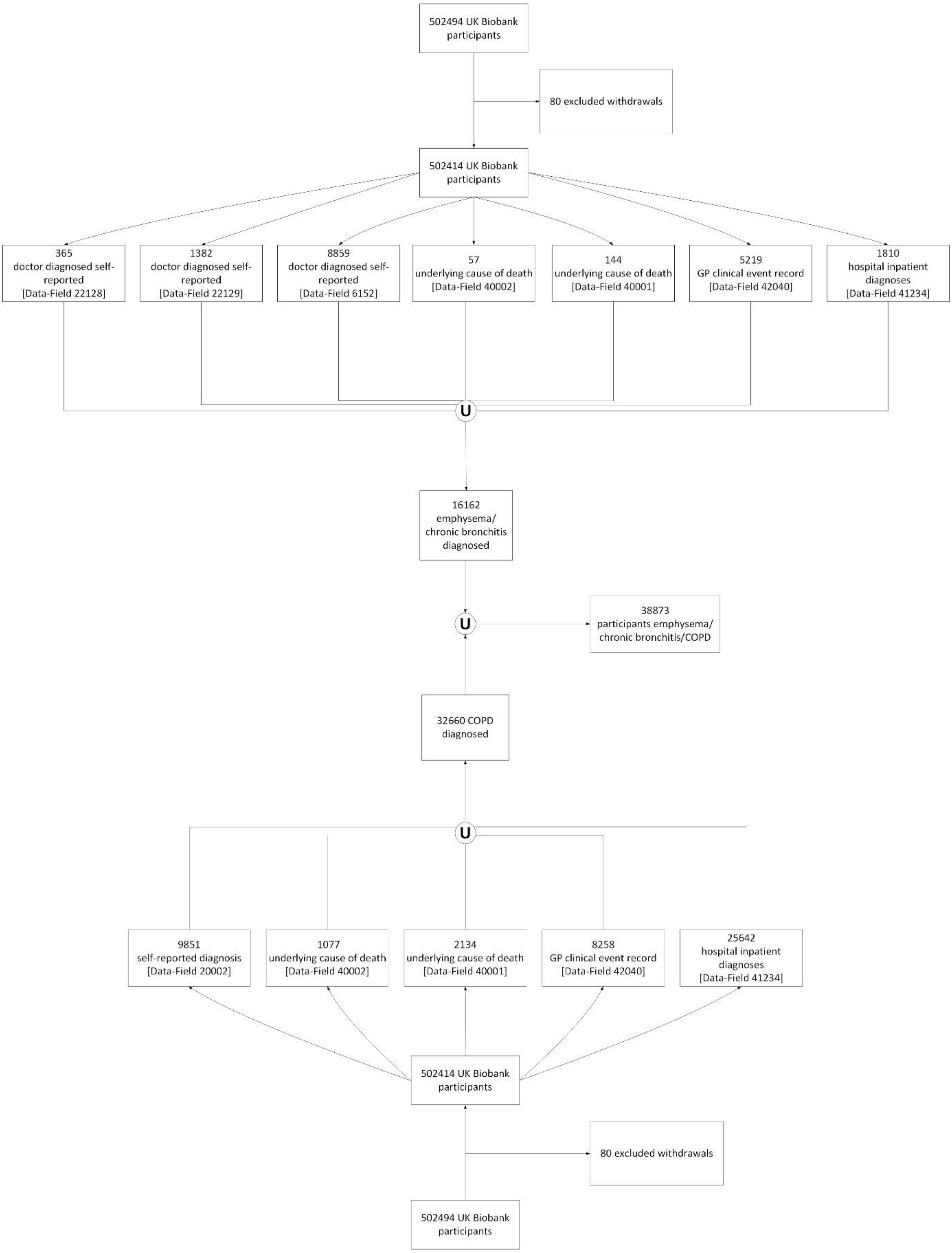

Figure S3

Flow-chart for defining other asthma in the GWAS of high treatment burden and/or worse outcome vs other asthma.

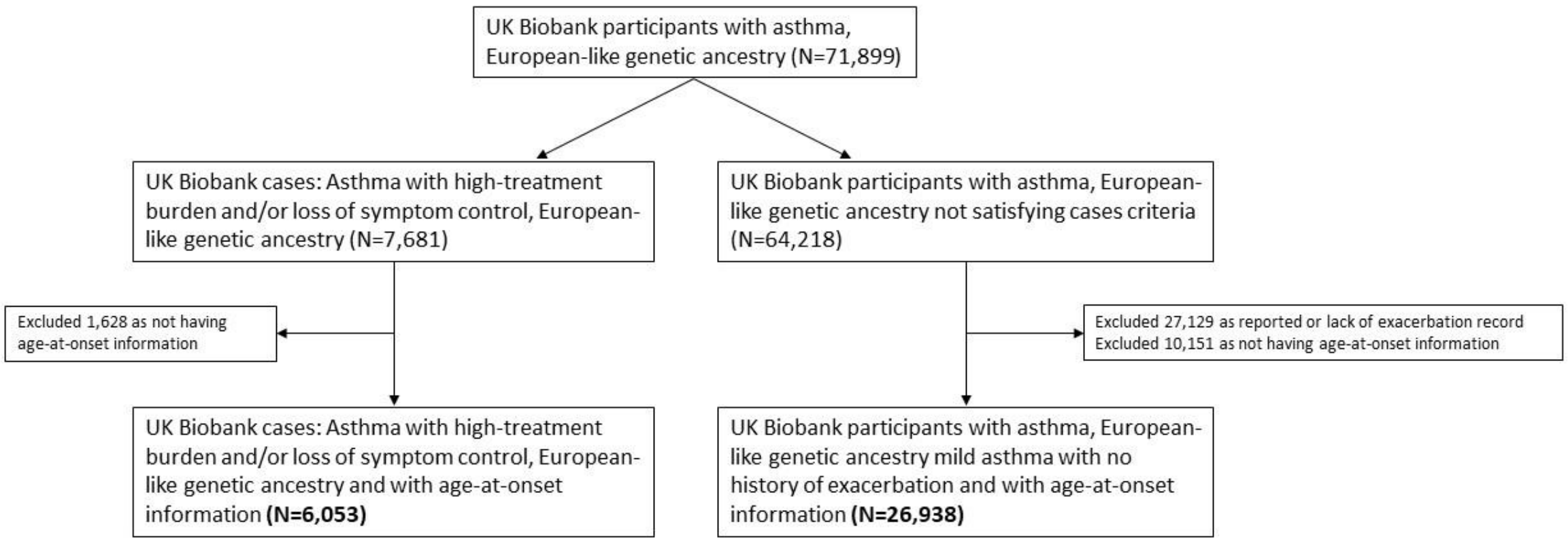

Figure S4

QQ-plot of discovery GWAS. p = p-value.

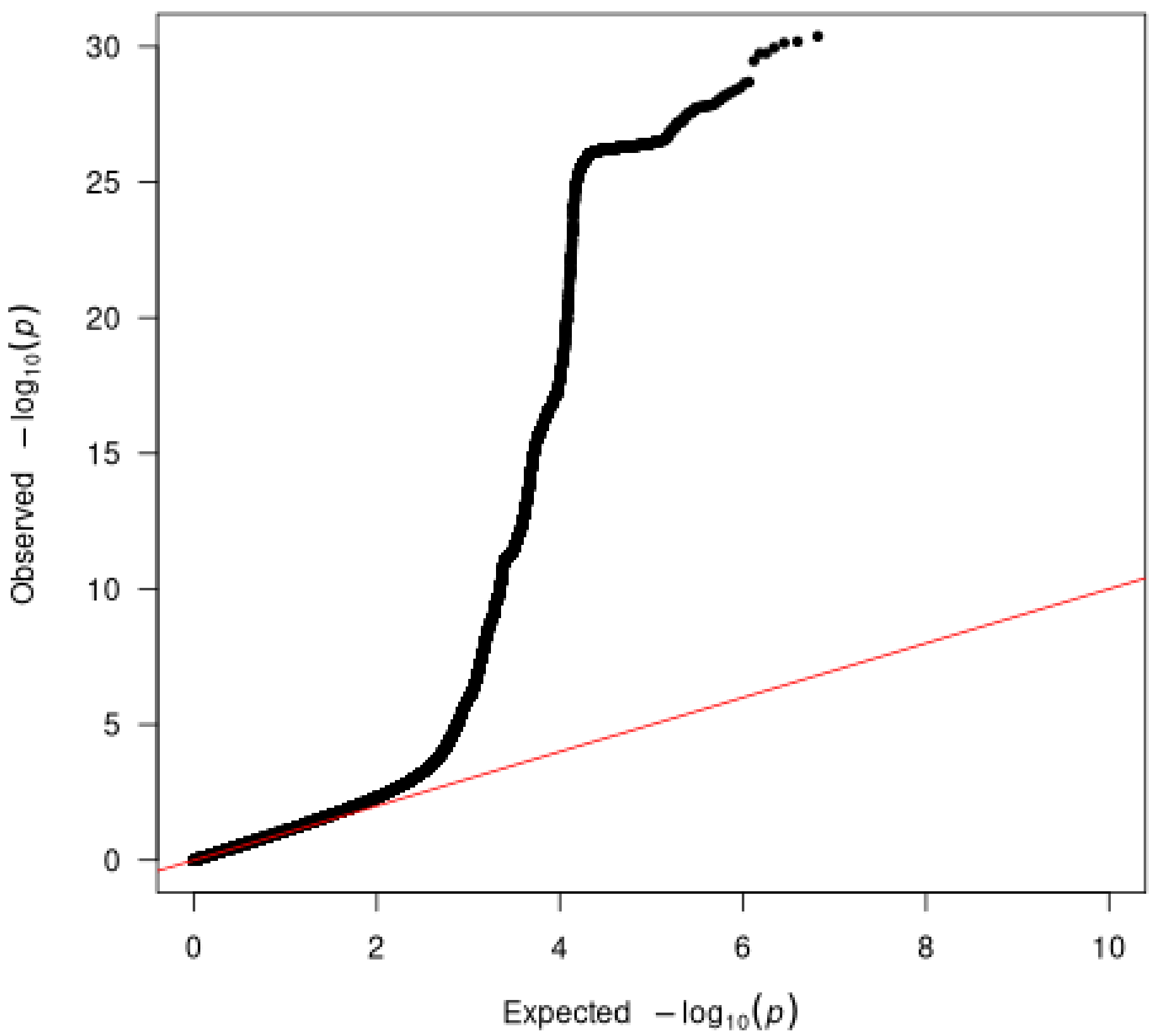

Figure S5

Scatter plot to compare genetic effect size and direction of effect for the sentinel variants between discovery GWAS and sensitivity analysis using BMI as covariate. After removing allergic condition, the genetic effect size showed a similar value and same direction of effect, although the association p-value is attenuated, probably due to the reduction in sample size. Variants with an effect size difference equal or higher than 0.1 were labelled. The Y axis represents the log OR.

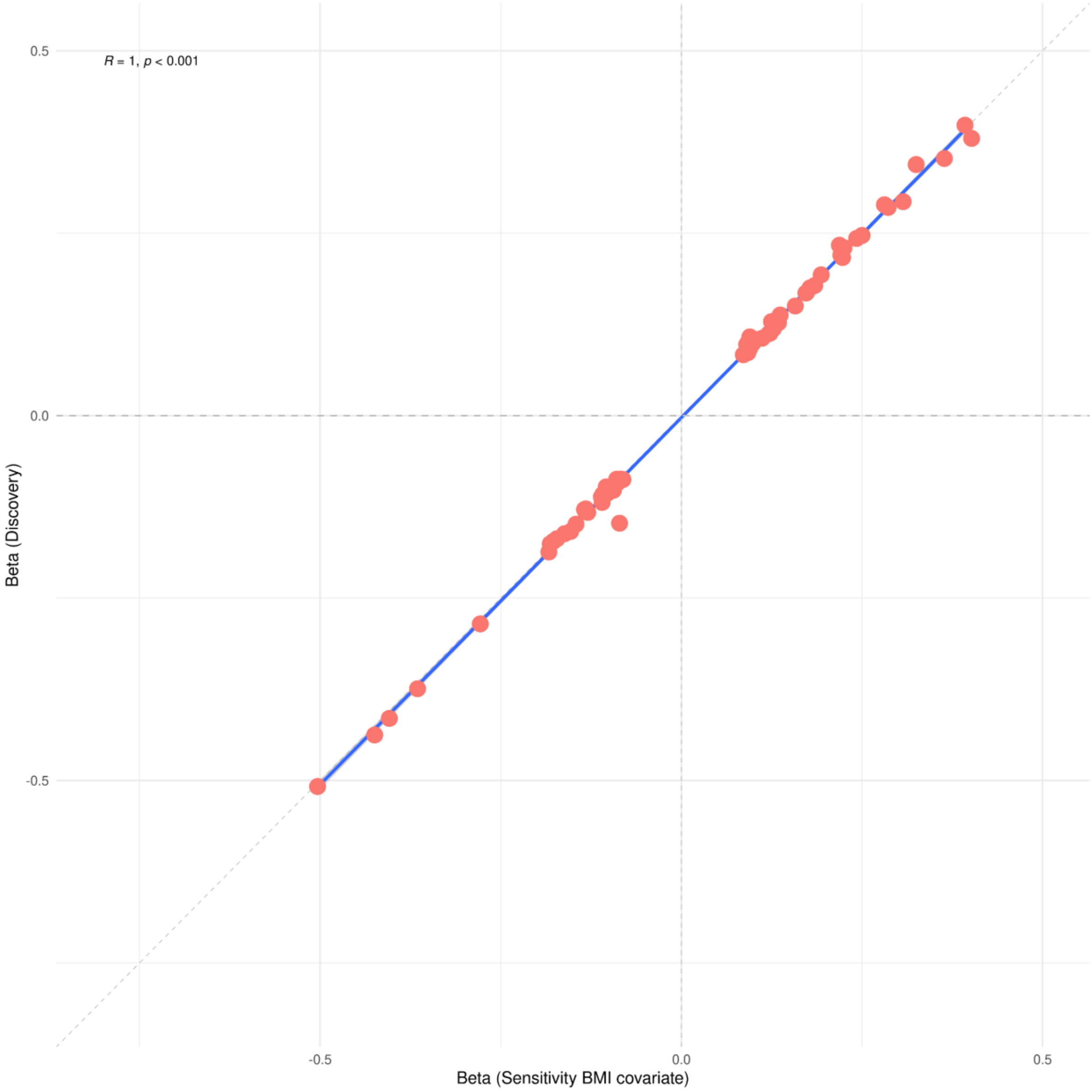

Figure S6

Scatter plot to compare genetic effect size and direction of effect for the sentinel variants between discovery GWAS and sensitivity analysis using smoking status as covariate. After removing allergic condition, the genetic effect size showed a similar value and same direction of effect, although the association p-value is attenuated, probably due to the reduction in sample size. Variants with an effect size difference equal or higher than 0.1 were labelled. The Y axis represents the log OR.

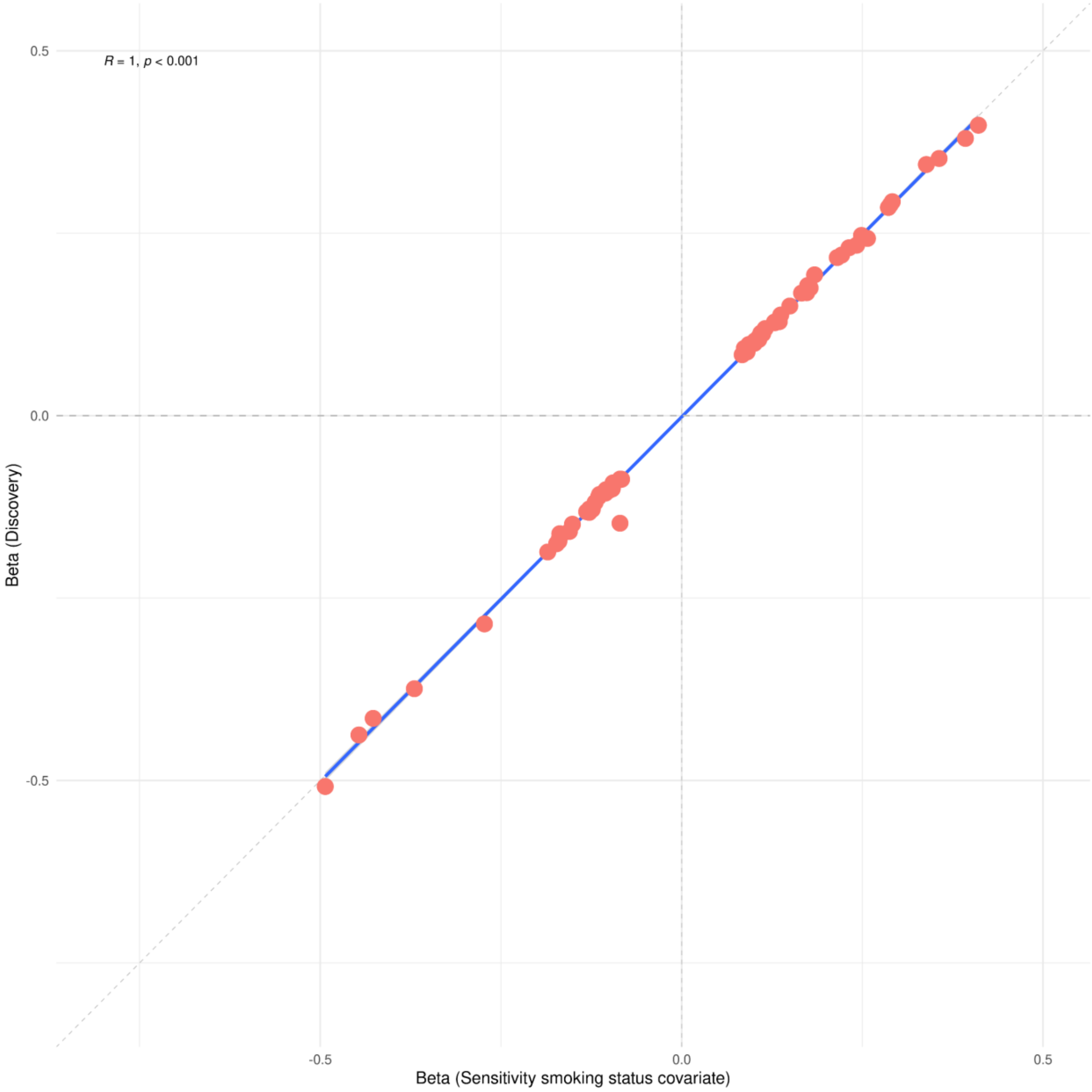

Figure S7

Scatter plot to compare genetic effect size and direction of effect for the sentinel variants between discovery GWAS and sensitivity analysis for allergy-free cohort. After removing allergic condition, the genetic effect size showed a similar value and same direction of effect, although the association p-value is attenuated, probably due to the reduction in sample size. Variants with an effect size difference equal or higher than 0.1 were labelled. The Y axis represents the log OR.

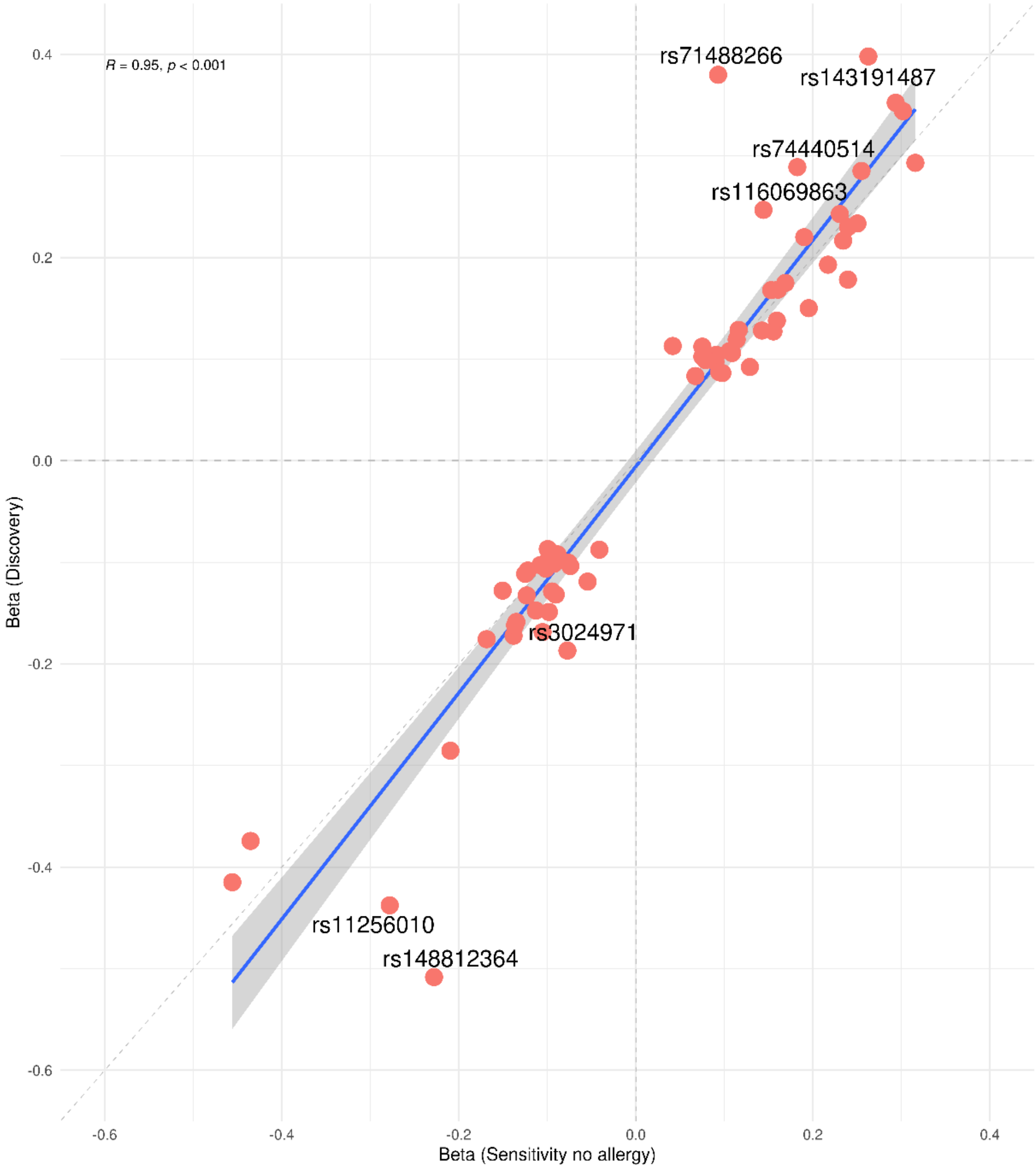

Figure S8

Genomic region plots of 21 replicated variants. The genomic region includes +/- 1Mb from the replicated sentinel SNPs (purple); the in-sample LD is with respect to the replicated sentinel SNPs.

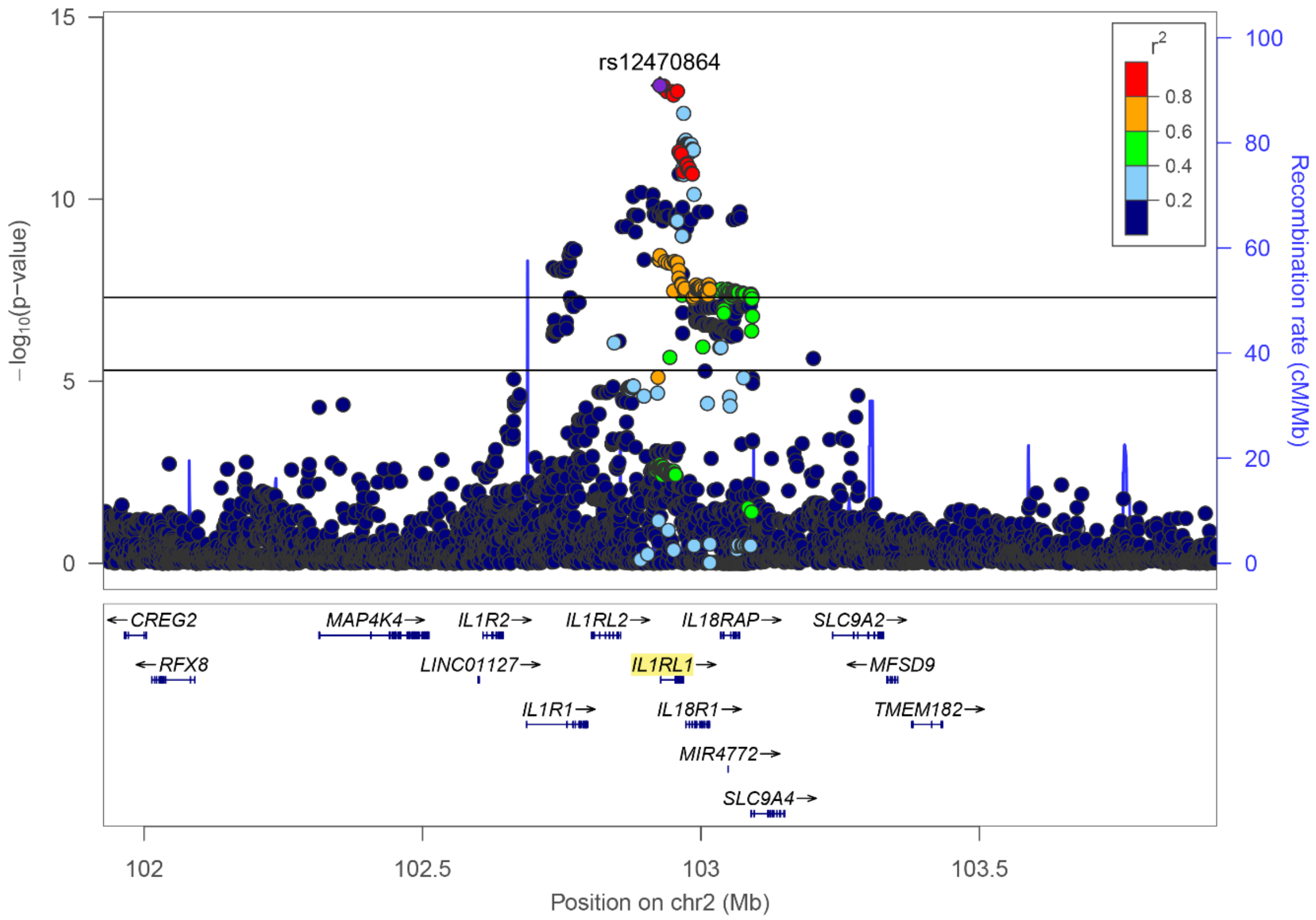

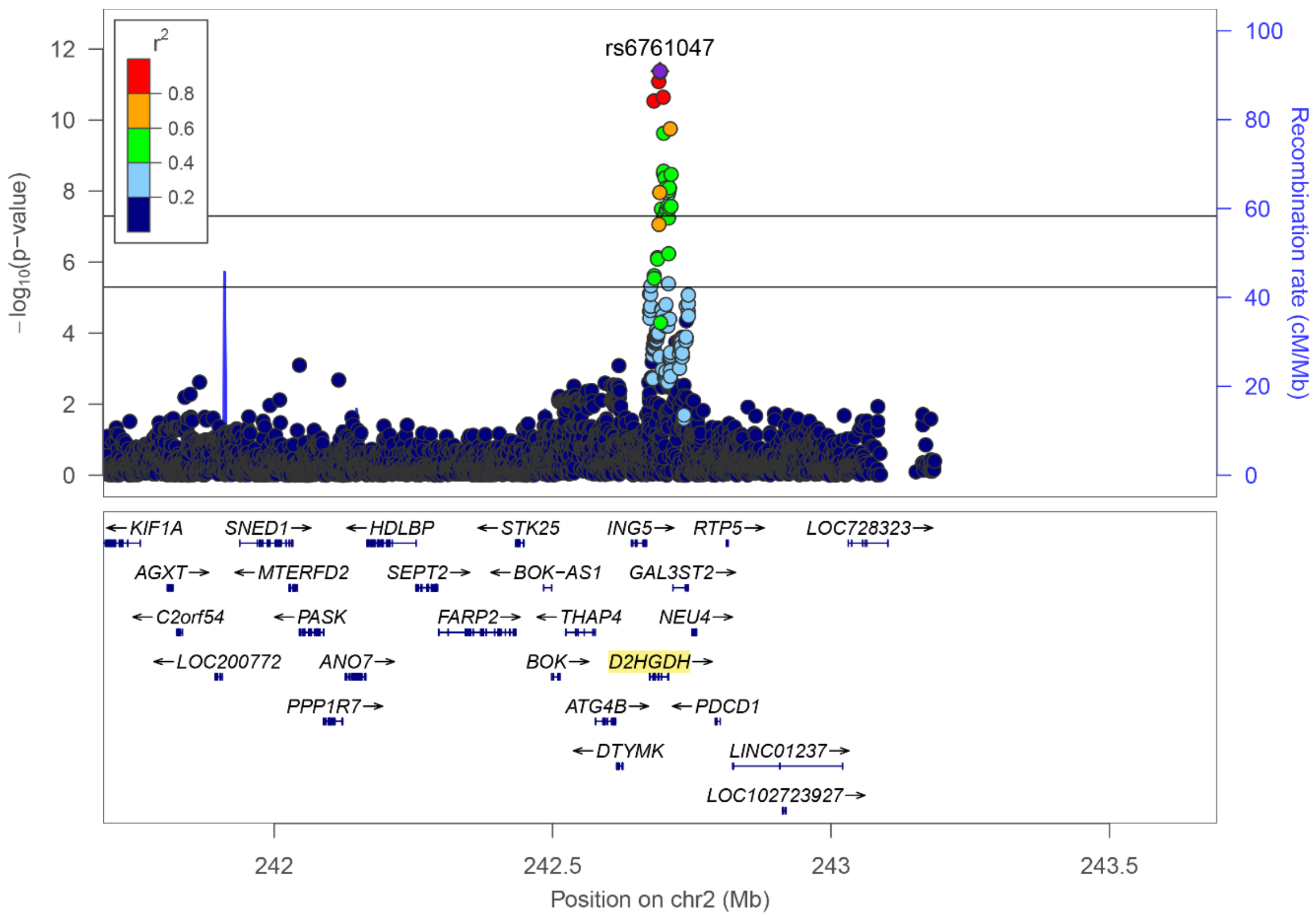

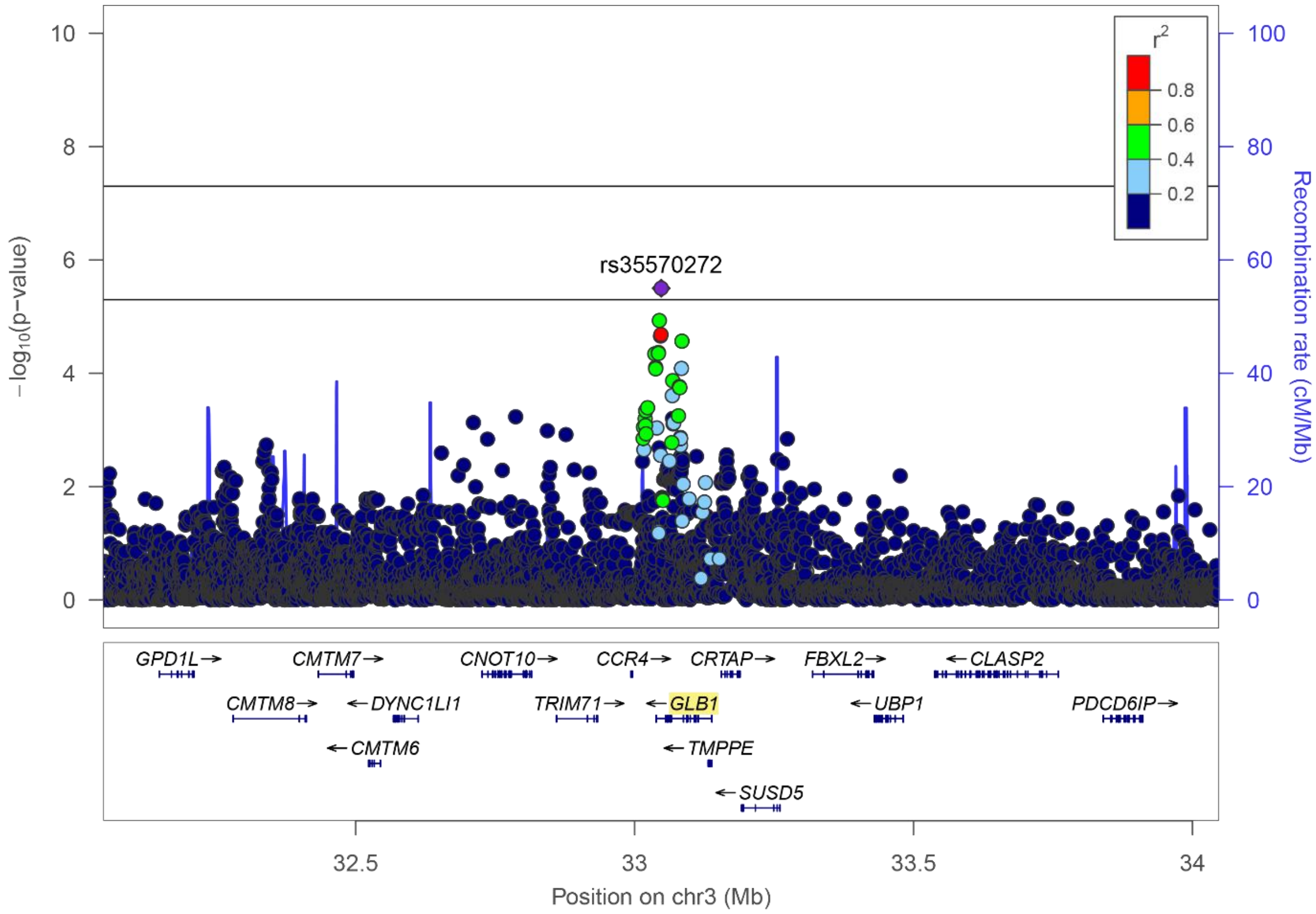
rs35570272
r2
0.8
0.6
0.4
0.2
−log10(p−value)
Recombination rate (cM/Mb)
GPD1L
CMTM7
CNOT10
CCR4
CRTAP
FBXL2
CLASP2
CMTM8
DYNC1LI1
TRIM71
GLB1
UBP1
PDCD6IP
CMTM6
TMPPE
SUSD5
Position on chr3 (Mb)

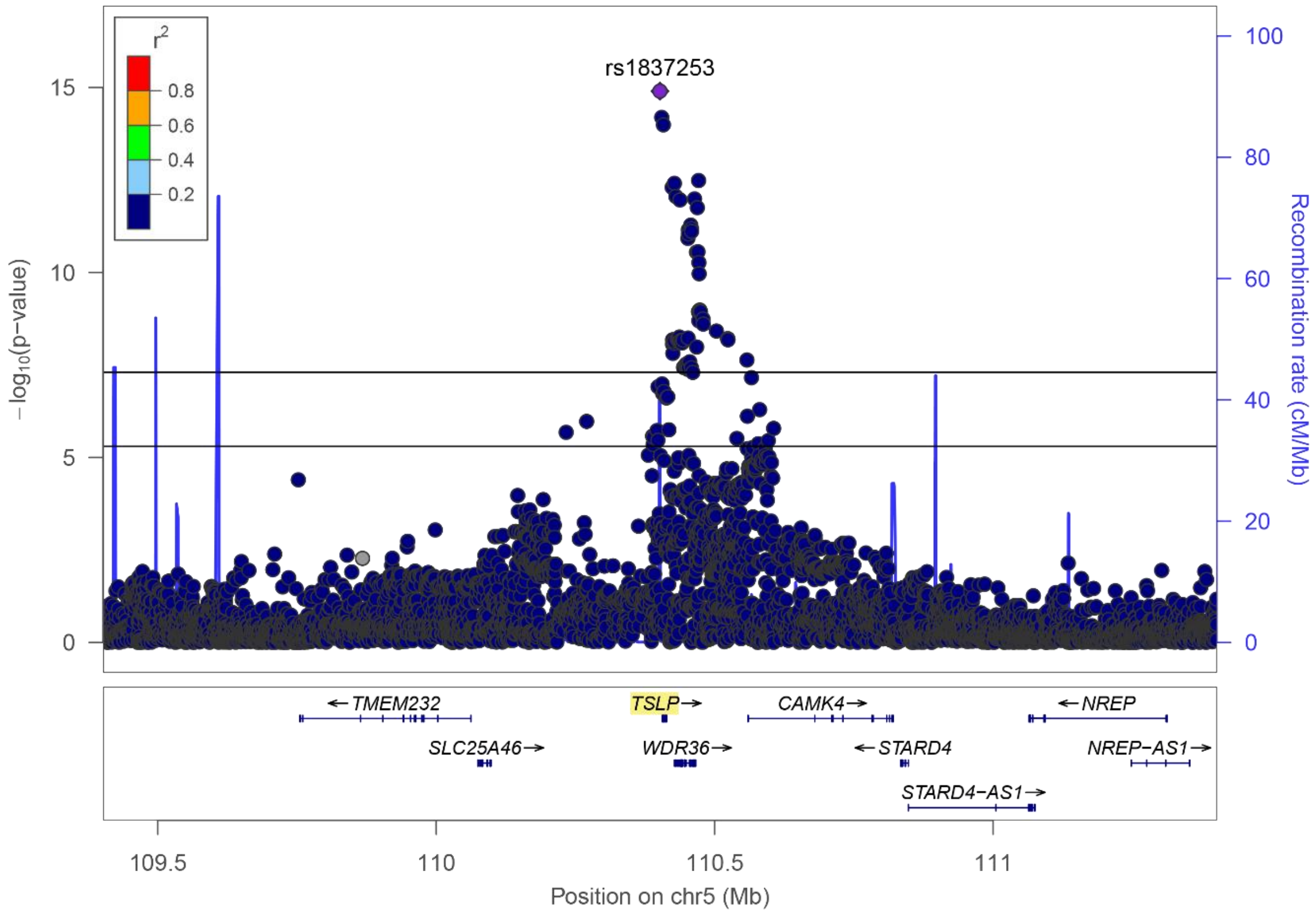
rs1837253
r2
0.8
0.6
0.4
0.2
−log10(p−value)
Recombination rate (cM/Mb)
TMEM232
TSLP
CAMK4
NREP
SLC25A46
WDR36
STARD4
NREP−AS1
STARD4−AS1
Position on chr5 (Mb)

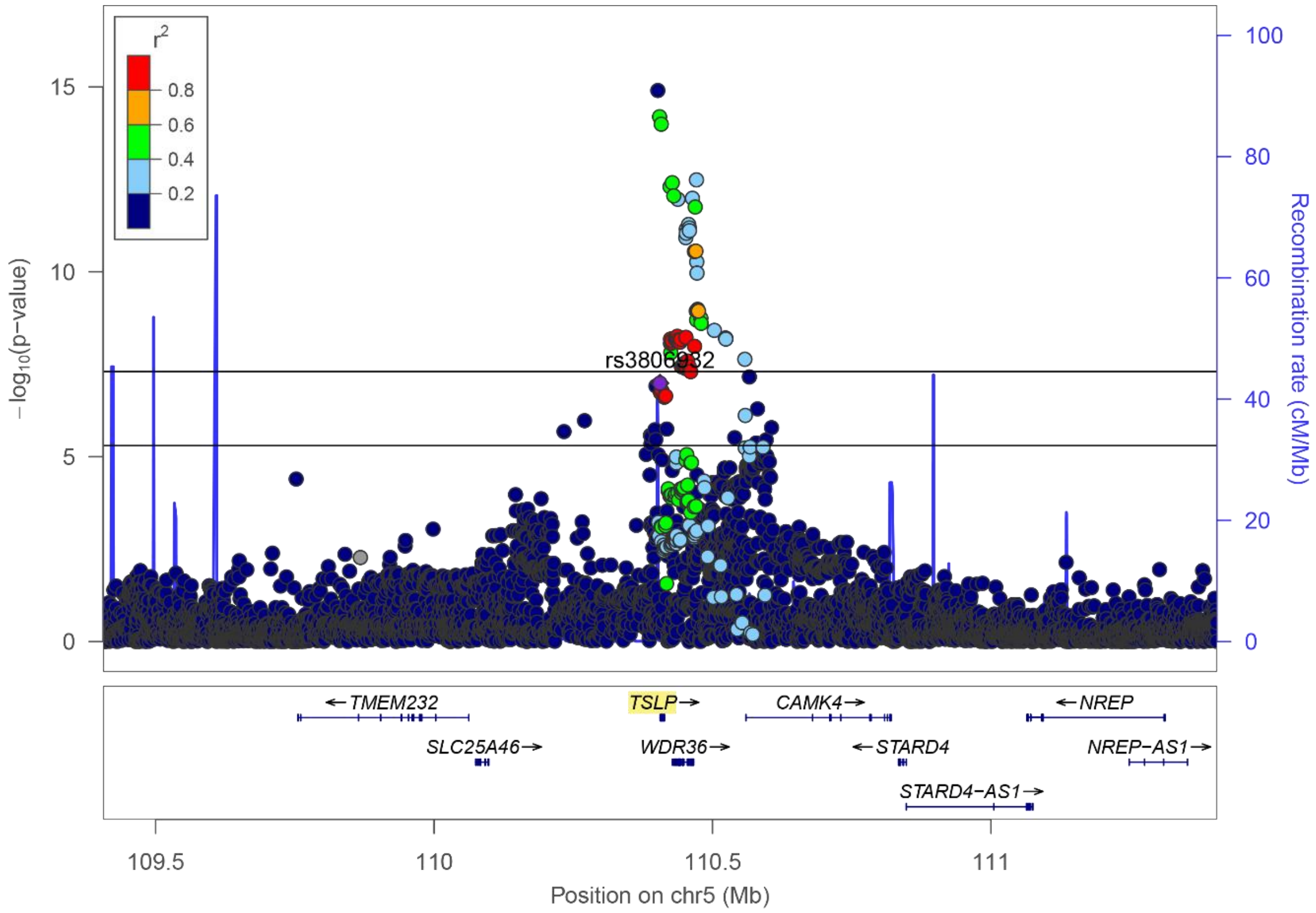
r2
0.8
0.6
0.4
0.2
15
10
5
0
−log10(p-value)
100
80
60
40
20
0
Recombination rate (cM/Mb)
rs3806932
TMEM232
TSLP
CAMK4
NREP
SLC25A46
WDR36
STARD4
NREP−AS1
STARD4−AS1
109.5
110
110.5
111
Position on chr5 (Mb)

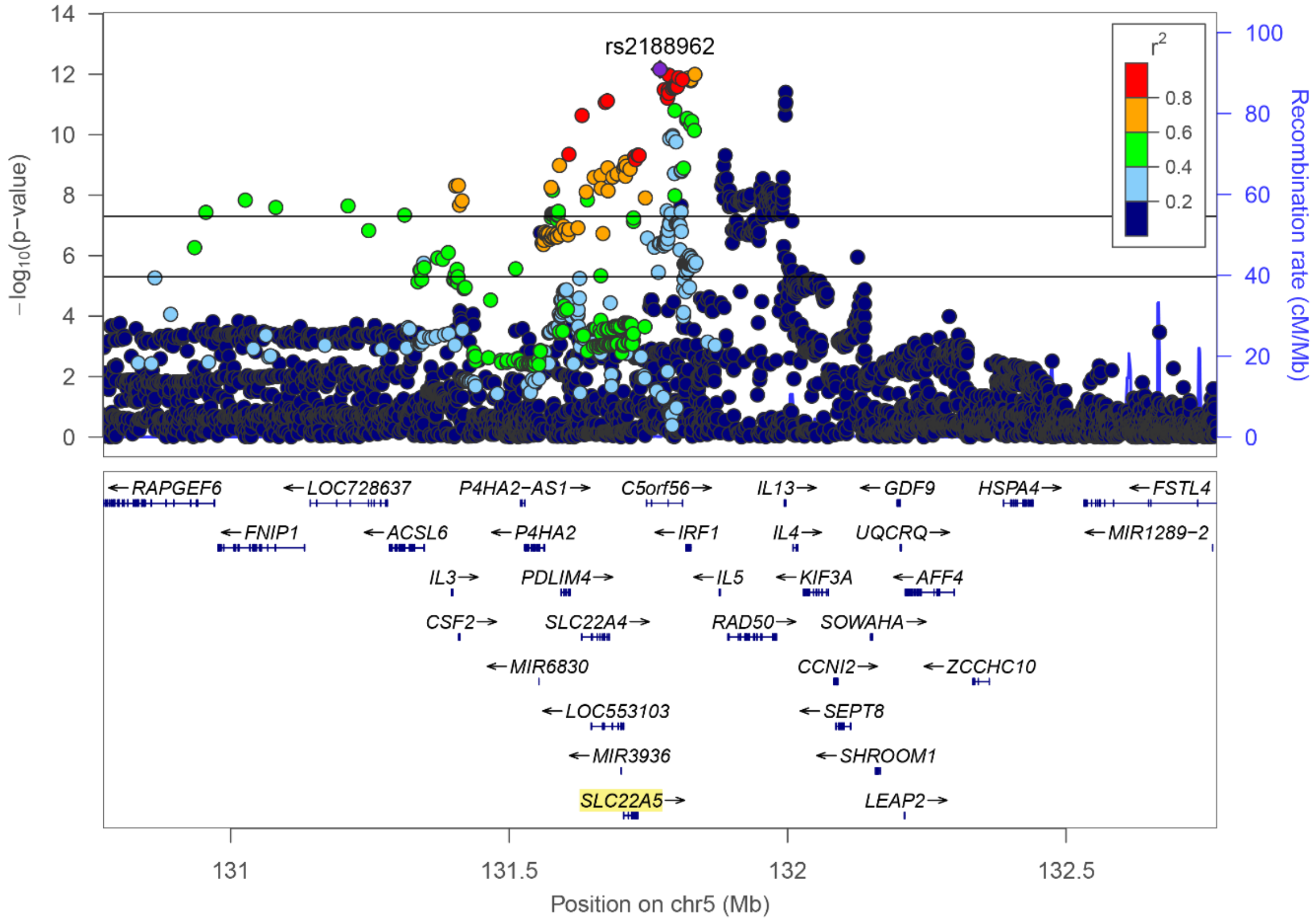
14
12
10
8
6
4
2
0
−log10(p-value)
rs2188962
r2
0.8
0.6
0.4
0.2
100
80
60
40
20
0
Recombination rate (cM/Mb)
RAPGEF6
LOC728637
P4HA2−AS1
C5orf56
IL13
GDF9
HSPA4
FSTL4
FNIP1
ACSL6
P4HA2
IRF1
IL4
UQCRQ
MIR1289−2
IL3
PDLIM4
IL5
KIF3A
AFF4
CSF2
SLC22A4
RAD50
SOWAHA
MIR6830
CCNI2
ZCCHC10
LOC553103
SEPT8
MIR3936
SHROOM1
SLC22A5
LEAP2
131
131.5
132
132.5
Position on chr5 (Mb)

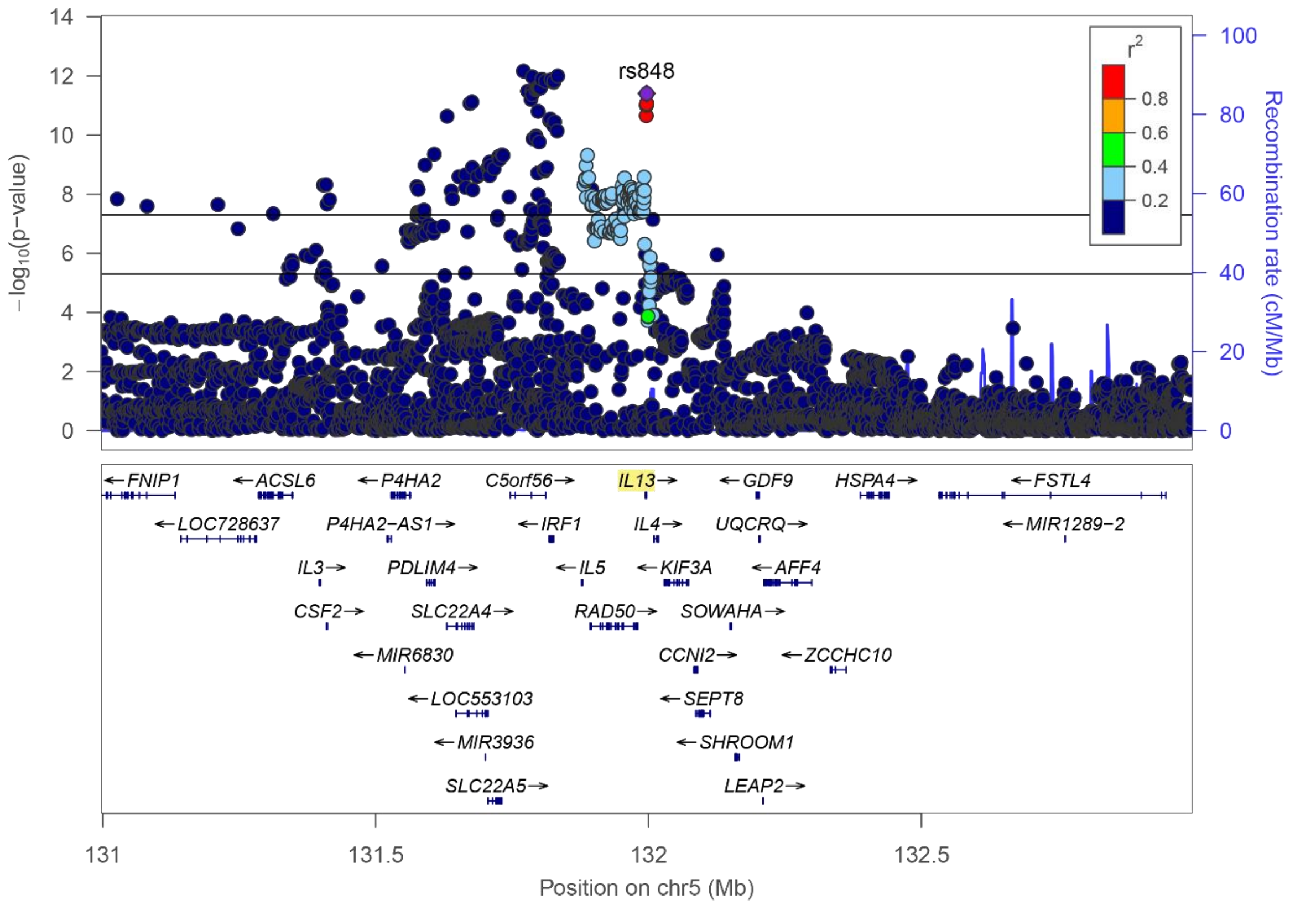
rs848
r2
0.8
0.6
0.4
0.2
−log10(p-value)
Recombination rate (cM/Mb)
←FNIP1
←ACSL6
←P4HA2
C5orf56→
IL13→
←GDF9
HSPA4→
←FSTL4
←LOC728637
P4HA2−AS1→
←IRF1
IL4→
UQCRQ→
←MIR1289−2
IL3→
PDLIM4→
←IL5
←KIF3A
←AFF4
CSF2→
SLC22A4→
RAD50→
SOWAHA→
←MIR6830
CCNI2→
←ZCCHC10
←LOC553103
←SEPT8
←MIR3936
←SHROOM1
SLC22A5→
LEAP2→
Position on chr5 (Mb)

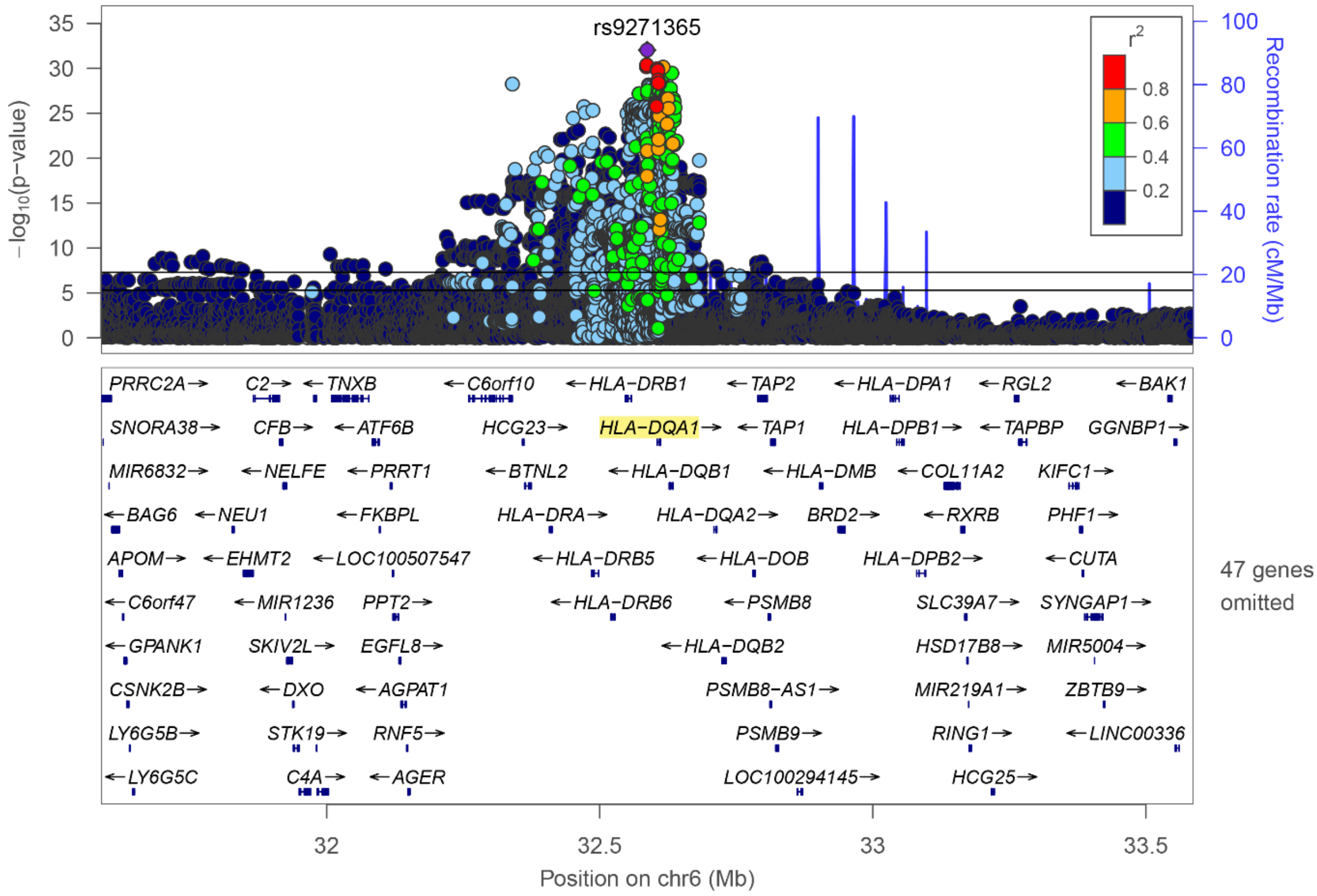
rs9271365
r2
0.8
0.6
0.4
0.2
−log10(p-value)
Recombination rate (cM/Mb)
PRRC2A→
C2→
←TNXB
←C6orf10
←HLA−DRB1
←TAP2
←HLA−DPA1
←RGL2
←BAK1
SNORA38→
CFB→
←ATF6B
HCG23→
HLA−DQA1→
←TAP1
HLA−DPB1→
←TAPBP
GGNBP1→
MIR6832→
←NELFE
←PRRT1
←BTNL2
←HLA−DQB1
←HLA−DMB
←COL11A2
KIFC1→
←BAG6
←NEU1
←FKBPL
HLA−DRA→
HLA−DQA2→
BRD2→
←RXRB
PHF1→
APOM→
←EHMT2
←LOC100507547
←HLA−DRB5
←HLA−DOB
HLA−DPB2→
←CUTA
←C6orf47
←MIR1236
PPT2→
←HLA−DRB6
←PSMB8
SLC39A7→
SYNGAP1→
←GPANK1
SKIV2L→
EGFL8→
←HLA−DQB2
HSD17B8→
MIR5004→
CSNK2B→
←DXO
←AGPAT1
PSMB8−AS1→
MIR219A1→
ZBTB9→
LY6G5B→
STK19→
RNF5→
PSMB9→
RING1→
←LINC00336
←LY6G5C
C4A→
←AGER
LOC100294145→
HCG25→
47 genes omitted
Position on chr6 (Mb)

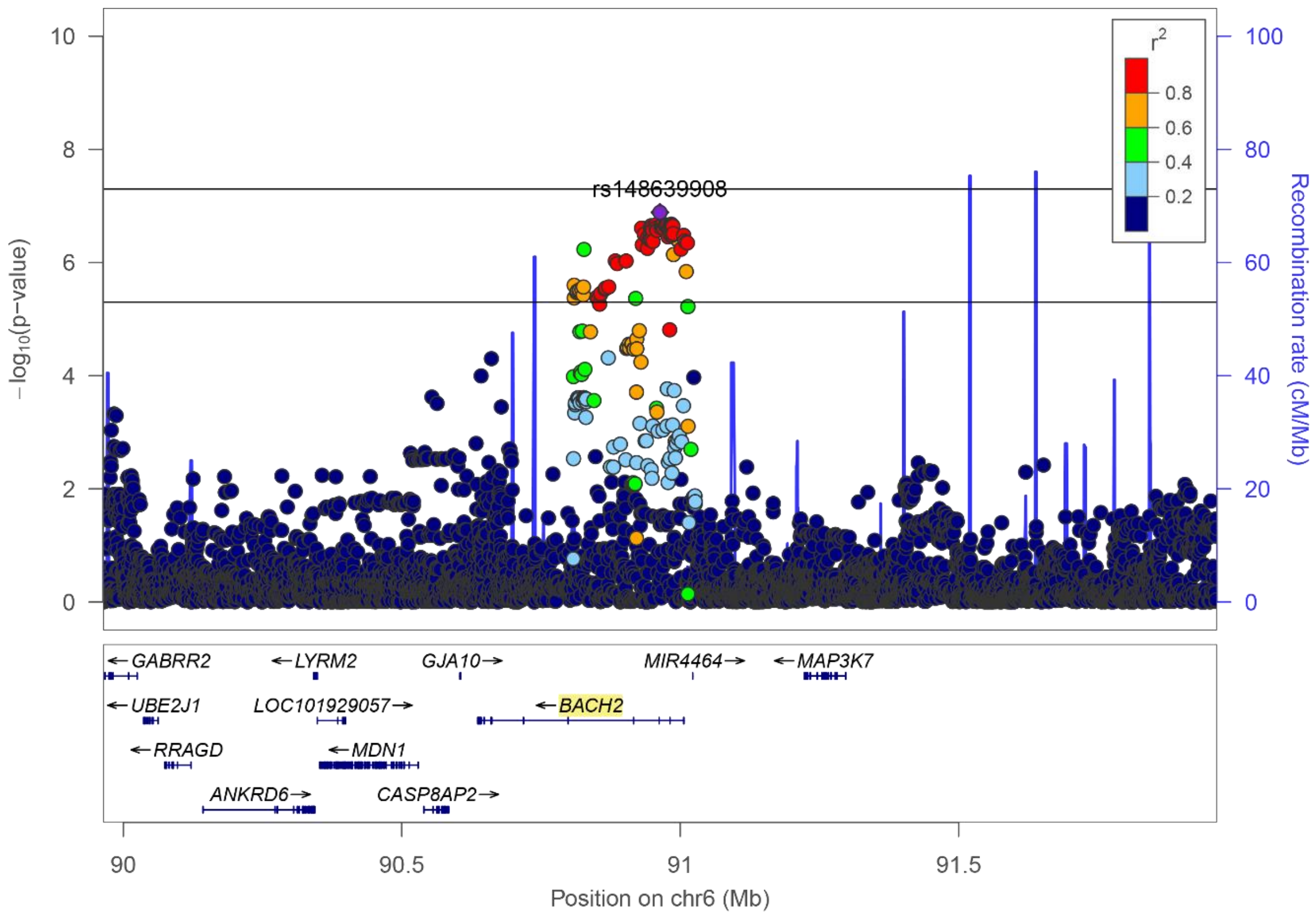

rs148639908
r2
0.8
0.6
0.4
0.2
−log10(p−value)
Recombination rate (cM/Mb)
GABRR2
LYRM2
GJA10
MIR4464
MAP3K7
UBE2J1
LOC101929057
BACH2
RRAGD
MDN1
ANKRD6
CASP8AP2
Position on chr6 (Mb)

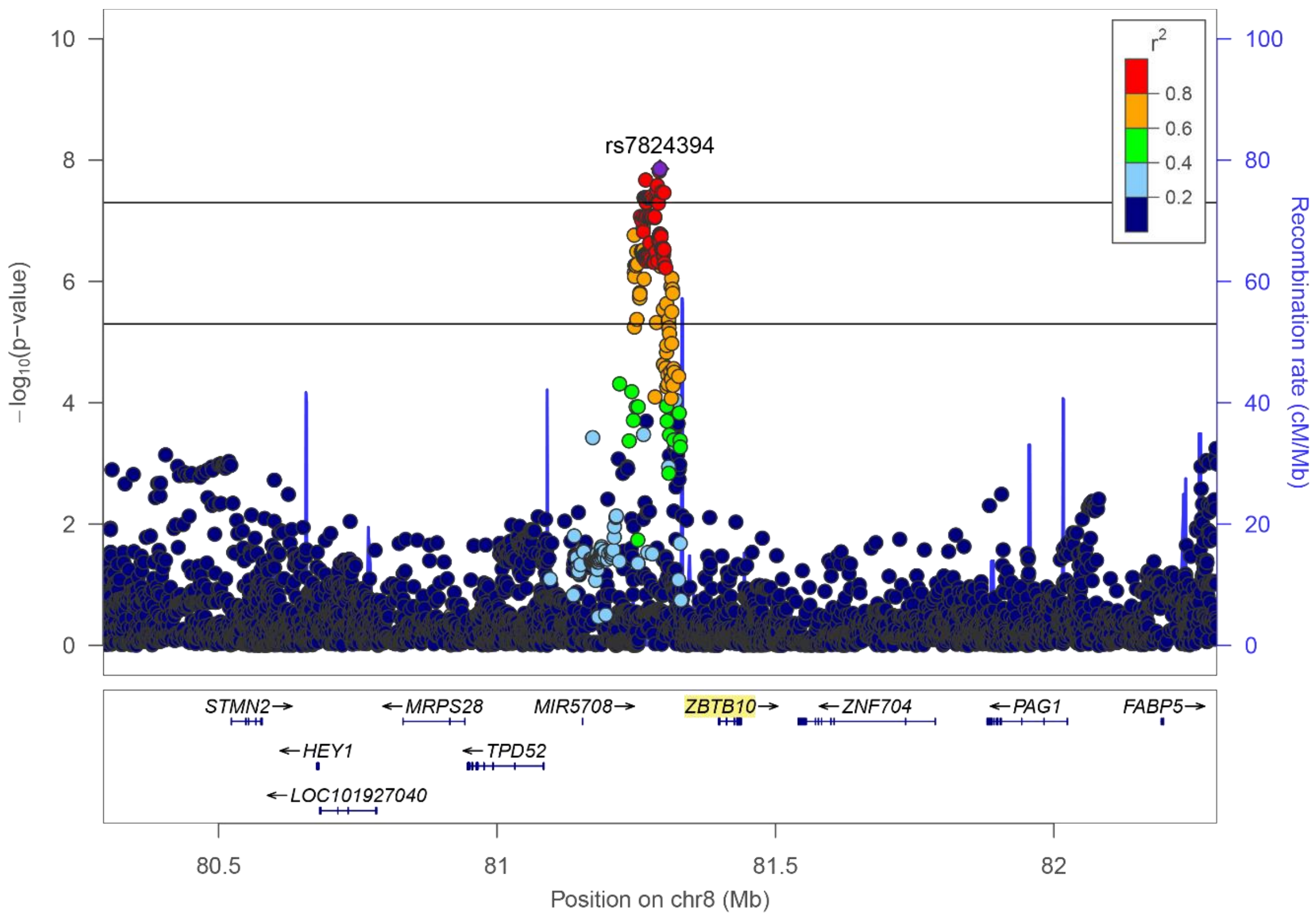

rs7824394
r2
0.8
0.6
0.4
0.2
−log10(p−value)
Recombination rate (cM/Mb)
STMN2
MRPS28
MIR5708
ZBTB10
ZNF704
PAG1
FABP5
HEY1
TPD52
LOC101927040
Position on chr8 (Mb)

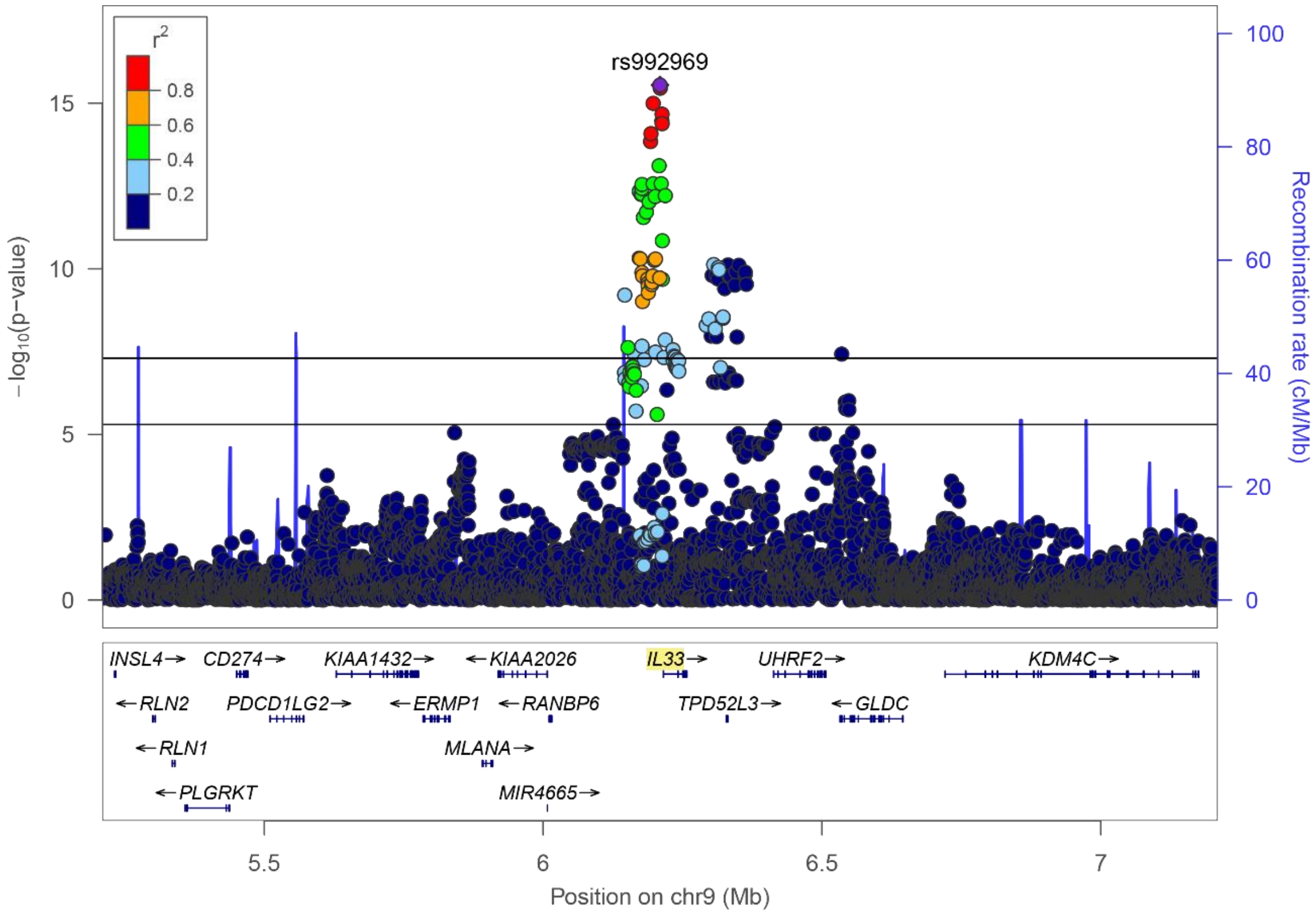
rs992969
r2
0.8
0.6
0.4
0.2
−log10(p−value)
Recombination rate (cM/Mb)
INSL4→
CD274→
KIAA1432→
←KIAA2026
IL33→
UHRF2→
KDM4C→
←RLN2
PDCD1LG2→
←ERMP1
←RANBP6
TPD52L3→
←GLDC
←RLN1
MLANA→
←PLGRKT
MIR4665→
Position on chr9 (Mb)

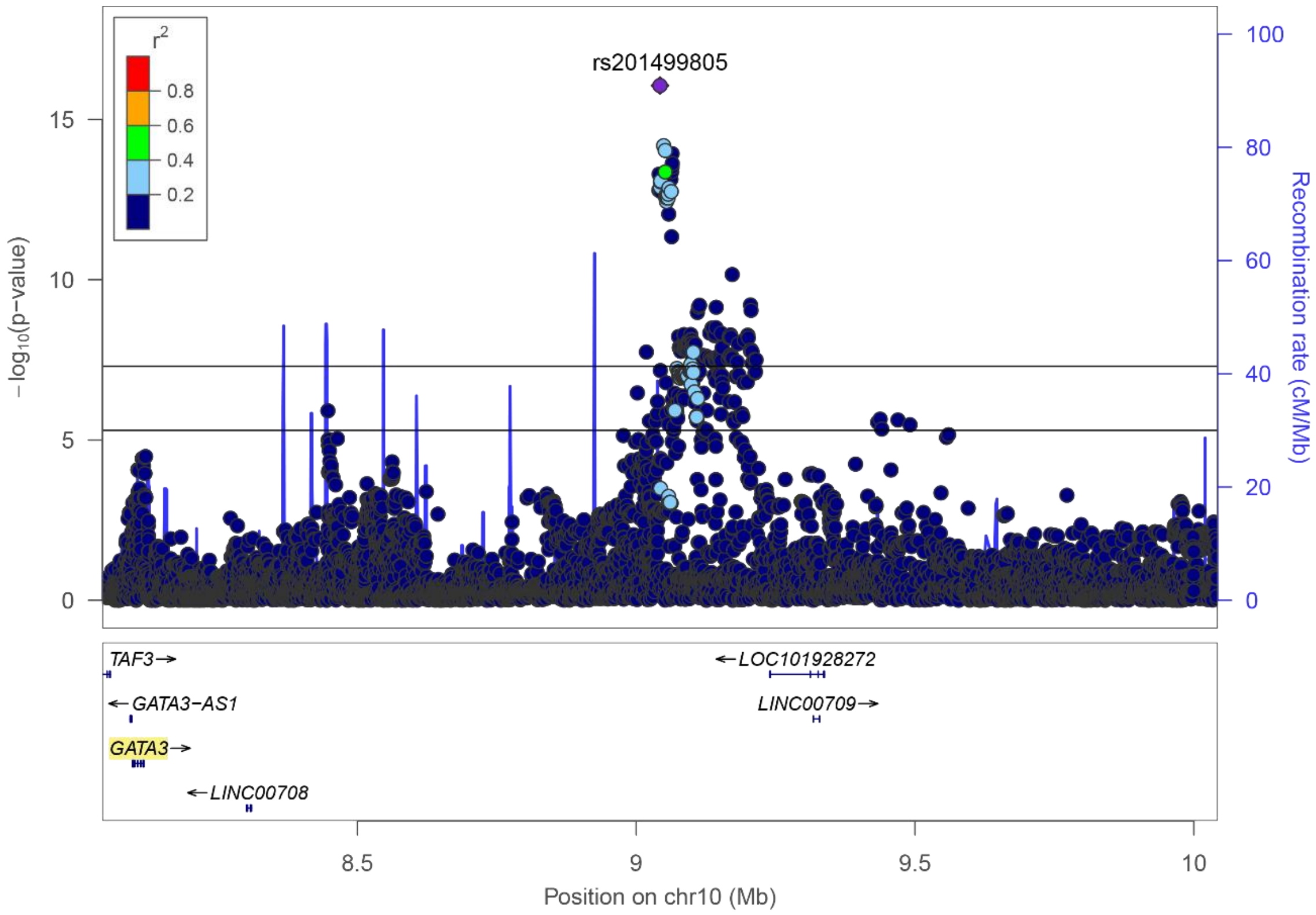
rs201499805
r2
0.8
0.6
0.4
0.2
−log10(p−value)
Recombination rate (cM/Mb)
TAF3→
←LOC101928272
←GATA3−AS1
LINC00709→
GATA3→
←LINC00708
Position on chr10 (Mb)

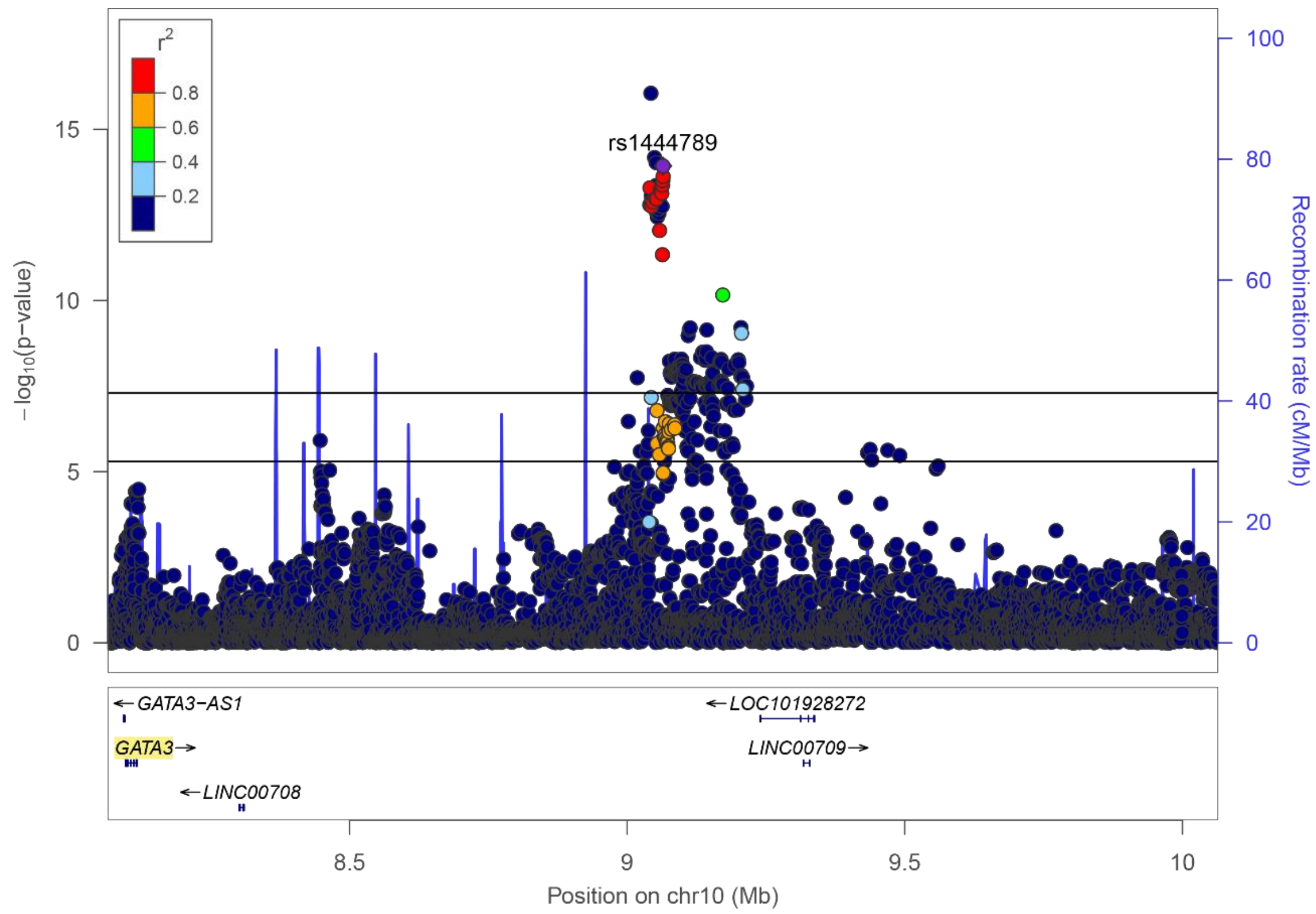

r2
0.8
0.6
0.4
0.2
rs1444789
−log10(p−value)
Recombination rate (cM/Mb)
GATA3−AS1
GATA3
LINC00708
LOC101928272
LINC00709
Position on chr10 (Mb)

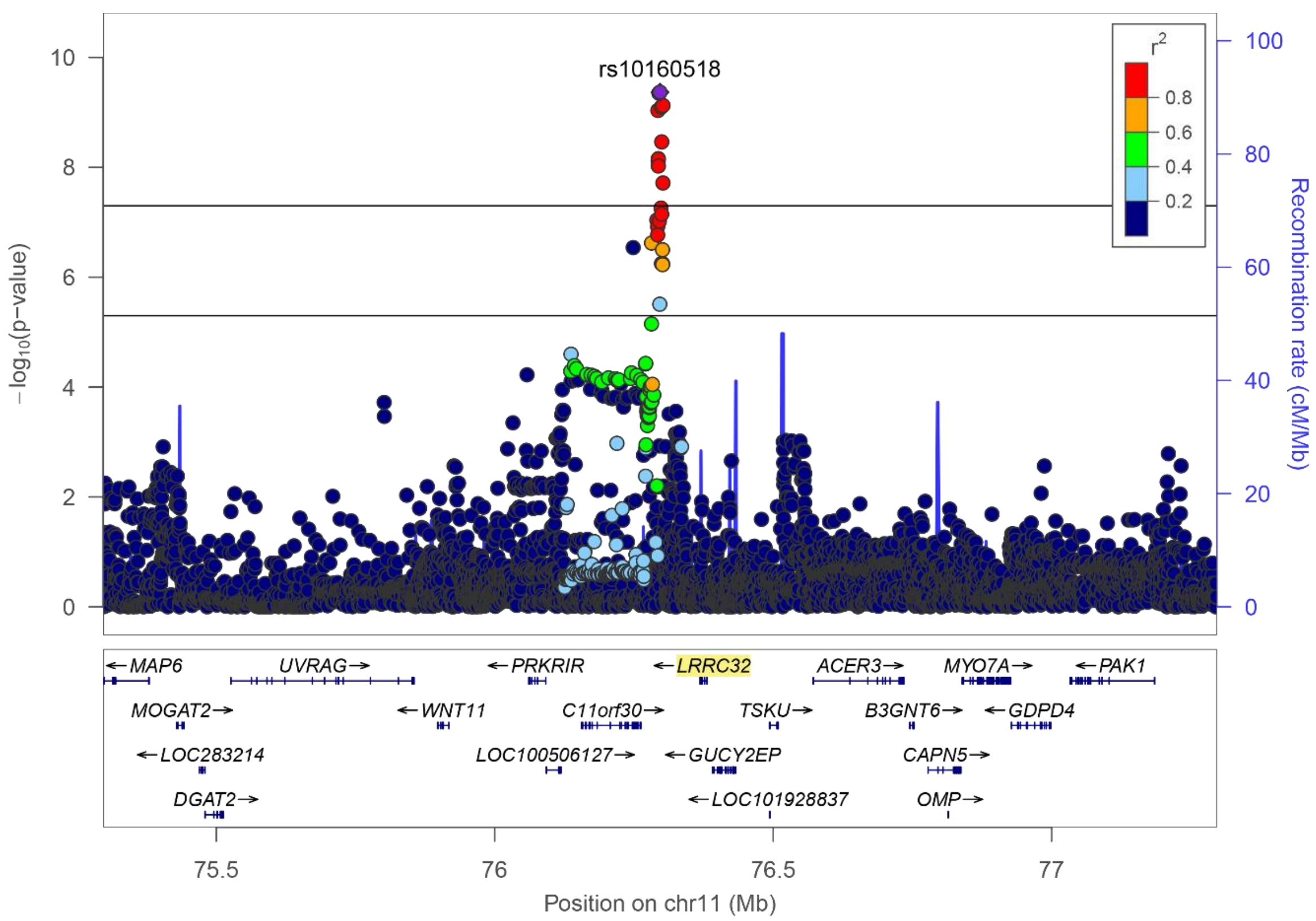

rs10160518
r2
0.8
0.6
0.4
0.2
−log10(p−value)
Recombination rate (cM/Mb)
MAP6
UVRAG
PRKRIR
LRRC32
ACER3
MYO7A
PAK1
MOGAT2
WNT11
C11orf30
TSKU
B3GNT6
GDPD4
LOC283214
LOC100506127
GUCY2EP
CAPN5
DGAT2
LOC101928837
OMP
Position on chr11 (Mb)

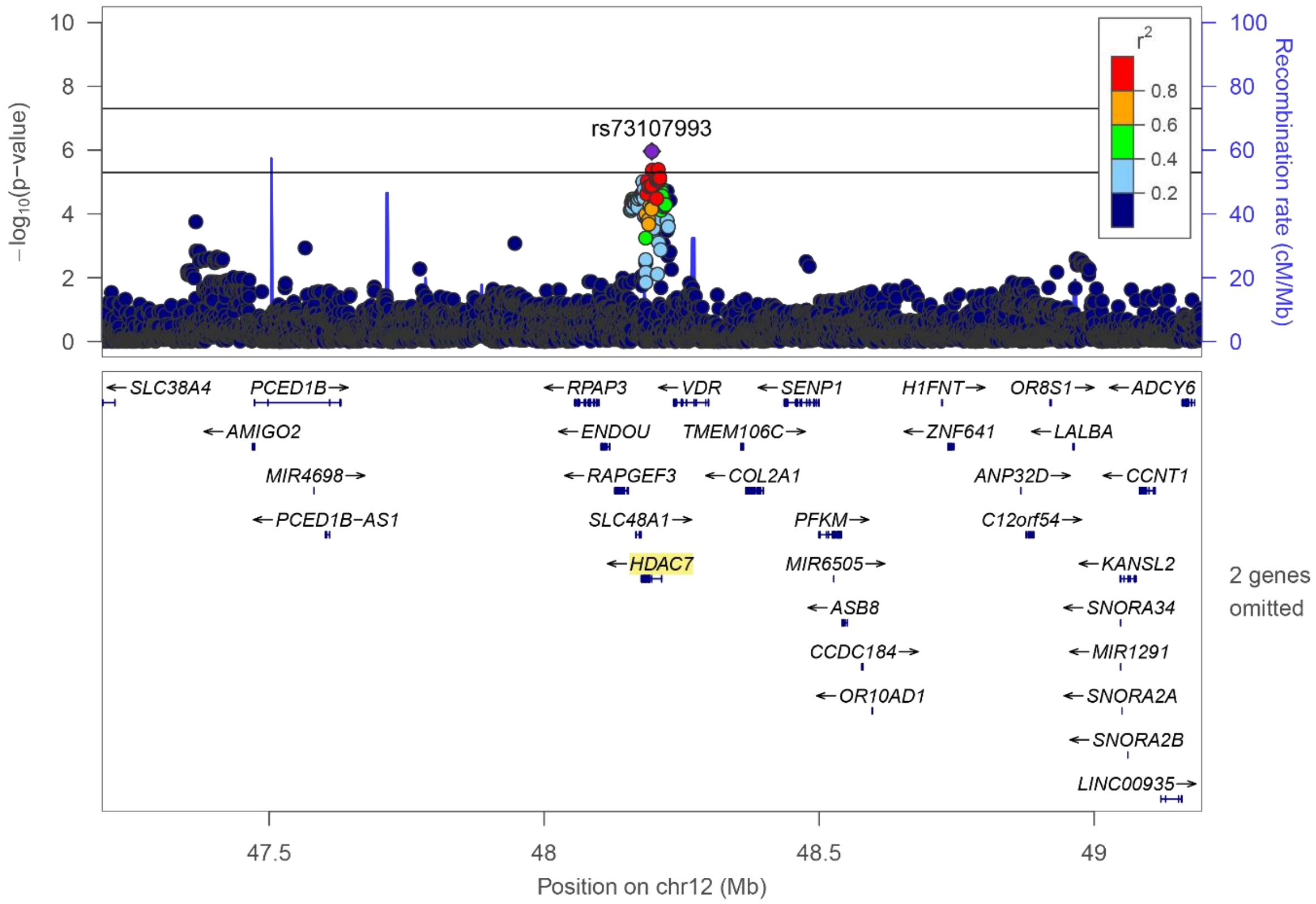

rs73107993
−log10(p−value)
Recombination rate (cM/Mb)
r2
0.8
0.6
0.4
0.2
SLC38A4
PCED1B
RPAP3
VDR
SENP1
H1FNT
OR8S1
ADCY6
AMIGO2
ENDOU
TMEM106C
ZNF641
LALBA
MIR4698
RAPGEF3
COL2A1
ANP32D
CCNT1
PCED1B−AS1
SLC48A1
PFKM
C12orf54
HDAC7
MIR6505
KANSL2
ASB8
SNORA34
CCDC184
MIR1291
OR10AD1
SNORA2A
SNORA2B
LINC00935
2 genes omitted
47.5
48
48.5
49
Position on chr12 (Mb)

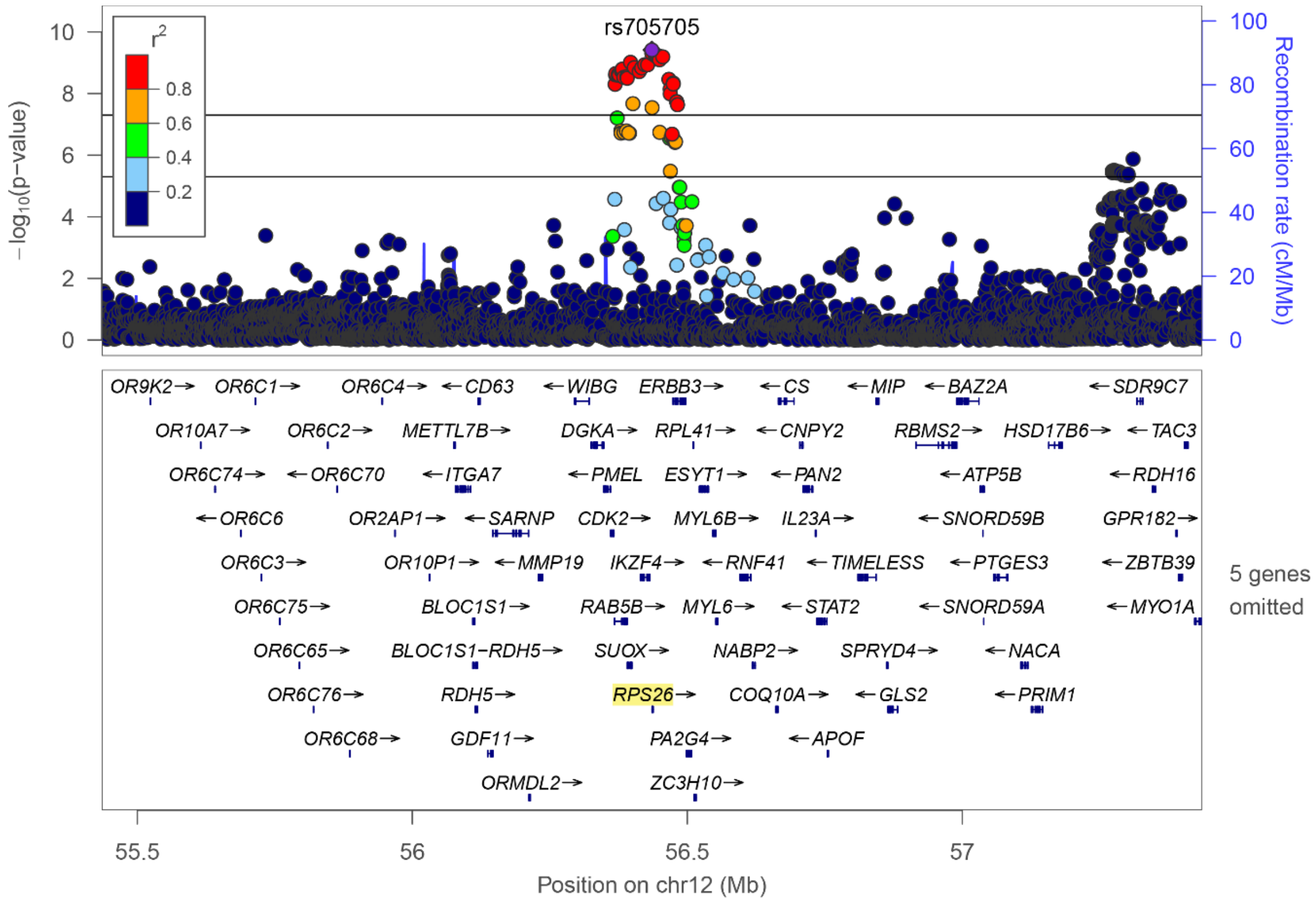

rs705705
−log10(p−value)
Recombination rate (cM/Mb)
r2
0.8
0.6
0.4
0.2
OR9K2
OR6C1
OR6C4
CD63
WIBG
ERBB3
CS
MIP
BAZ2A
SDR9C7
OR10A7
OR6C2
METTL7B
DGKA
RPL41
CNPY2
RBMS2
HSD17B6
TAC3
OR6C74
OR6C70
ITGA7
PMEL
ESYT1
PAN2
ATP5B
RDH16
OR6C6
OR2AP1
SARNP
CDK2
MYL6B
IL23A
SNORD59B
GPR182
OR6C3
OR10P1
MMP19
IKZF4
RNF41
TIMELESS
PTGES3
ZBTB39
OR6C75
BLOC1S1
RAB5B
MYL6
STAT2
SNORD59A
MYO1A
OR6C65
BLOC1S1−RDH5
SUOX
NABP2
SPRYD4
NACA
OR6C76
RDH5
RPS26
COQ10A
GLS2
PRIM1
OR6C68
GDF11
PA2G4
APOF
ORMDL2
ZC3H10
5 genes omitted
55.5
56
56.5
57
Position on chr12 (Mb)

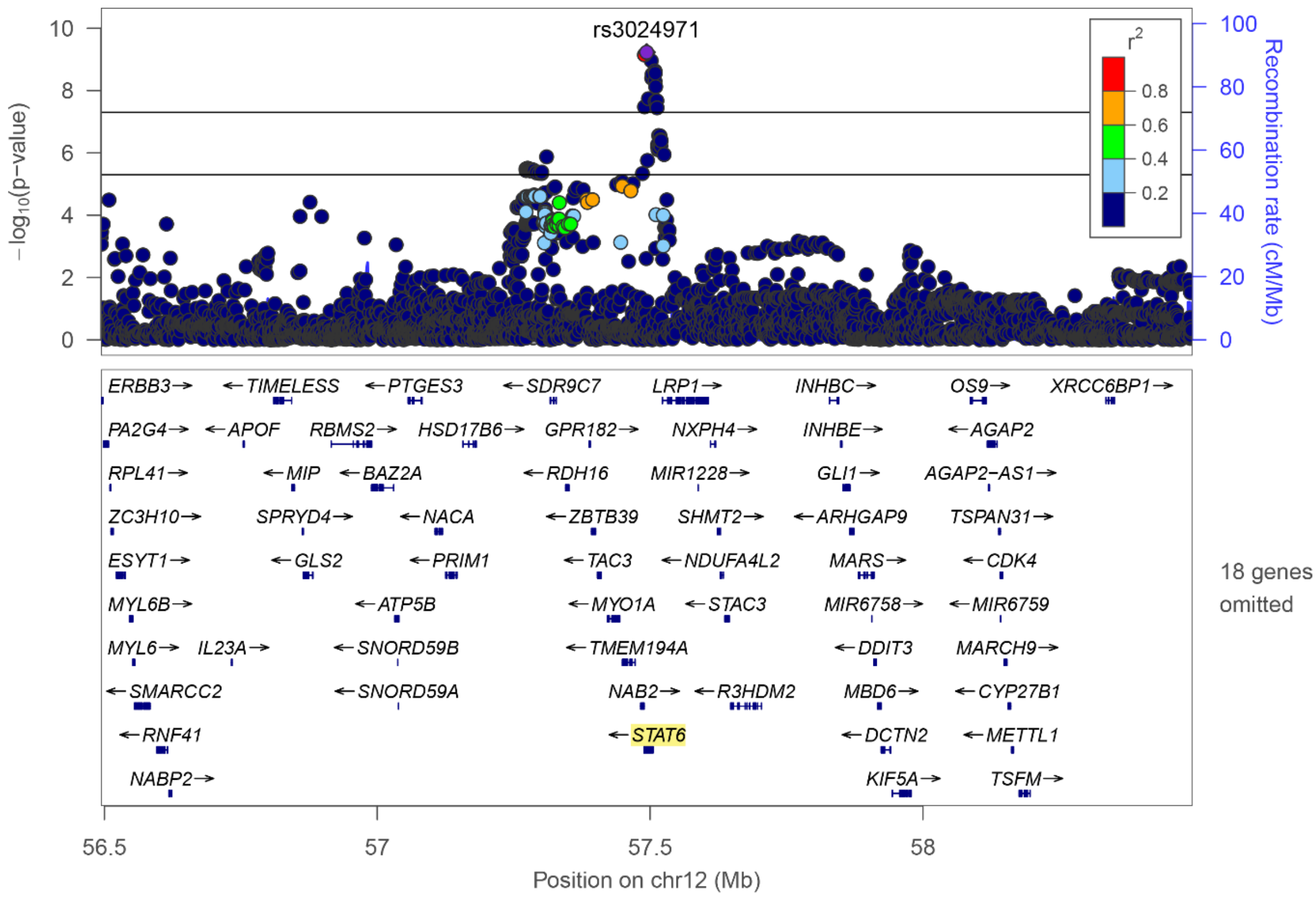

rs3024971
r2
0.8
0.6
0.4
0.2
−log10(p−value)
Recombination rate (cM/Mb)
ERBB3→
←TIMELESS
←PTGES3
←SDR9C7
LRP1→
INHBC→
OS9→
XRCC6BP1→
PA2G4→
←APOF
RBMS2→
HSD17B6→
GPR182→
NXPH4→
INHBE→
←AGAP2
RPL41→
←MIP
←BAZ2A
←RDH16
MIR1228→
GLI1→
AGAP2−AS1→
ZC3H10→
SPRYD4→
←NACA
←ZBTB39
SHMT2→
←ARHGAP9
TSPAN31→
ESYT1→
←GLS2
←PRIM1
←TAC3
←NDUFA4L2
MARS→
←CDK4
MYL6B→
←ATP5B
←MYO1A
←STAC3
MIR6758→
←MIR6759
MYL6→
IL23A→
←SNORD59B
←TMEM194A
←DDIT3
MARCH9→
←SMARCC2
←SNORD59A
NAB2→
←R3HDM2
MBD6→
←CYP27B1
←RNF41
←STAT6
←DCTN2
←METTL1
NABP2→
KIF5A→
TSFM→
18 genes omitted
56.5
57
57.5
58
Position on chr12 (Mb)

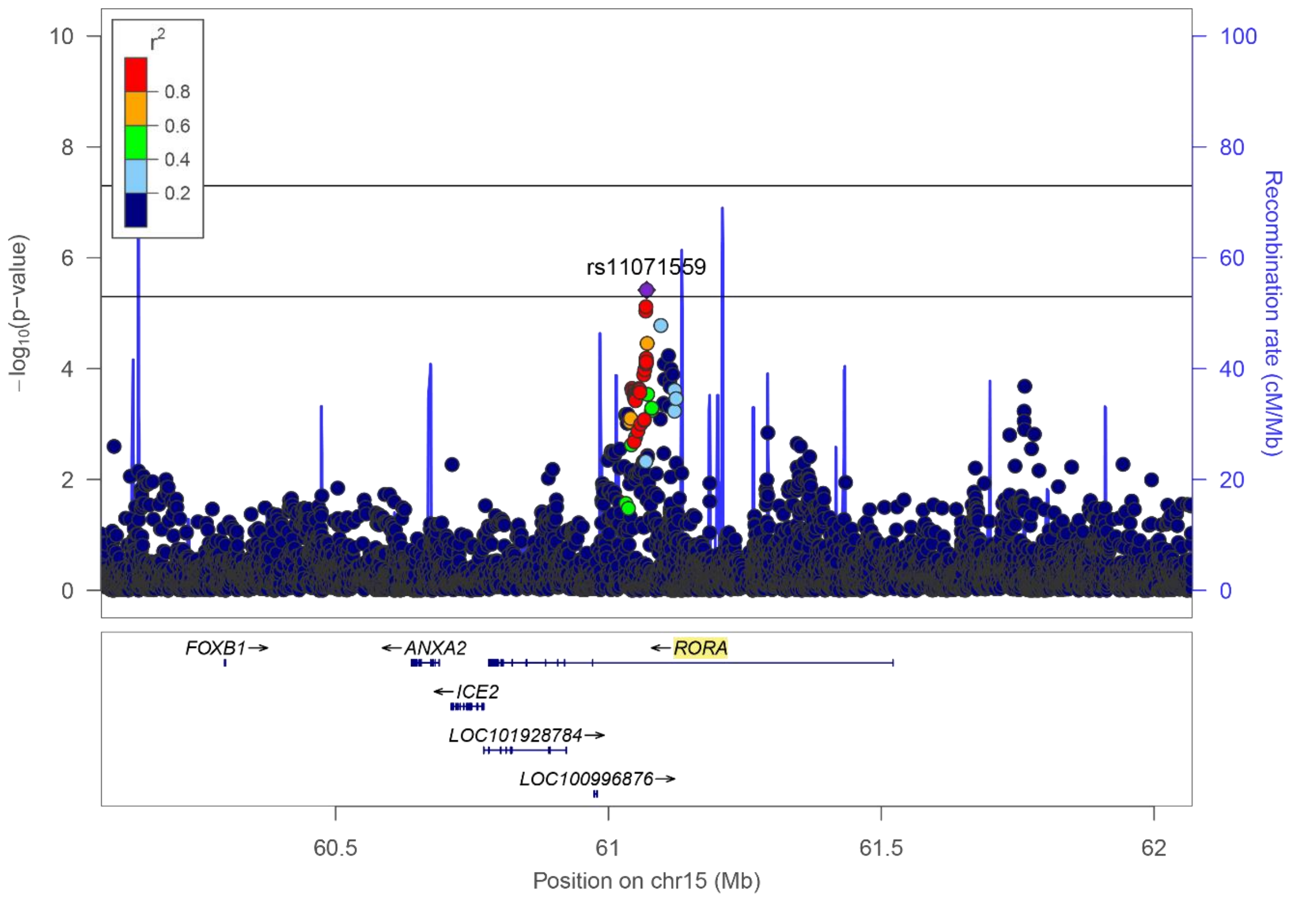

r2
0.8
0.6
0.4
0.2
rs11071559
−log10(p−value)
Recombination rate (cM/Mb)
FOXB1→
←ANXA2
←RORA
←ICE2
LOC101928784→
LOC100996876→
60.5
61
61.5
62
Position on chr15 (Mb)

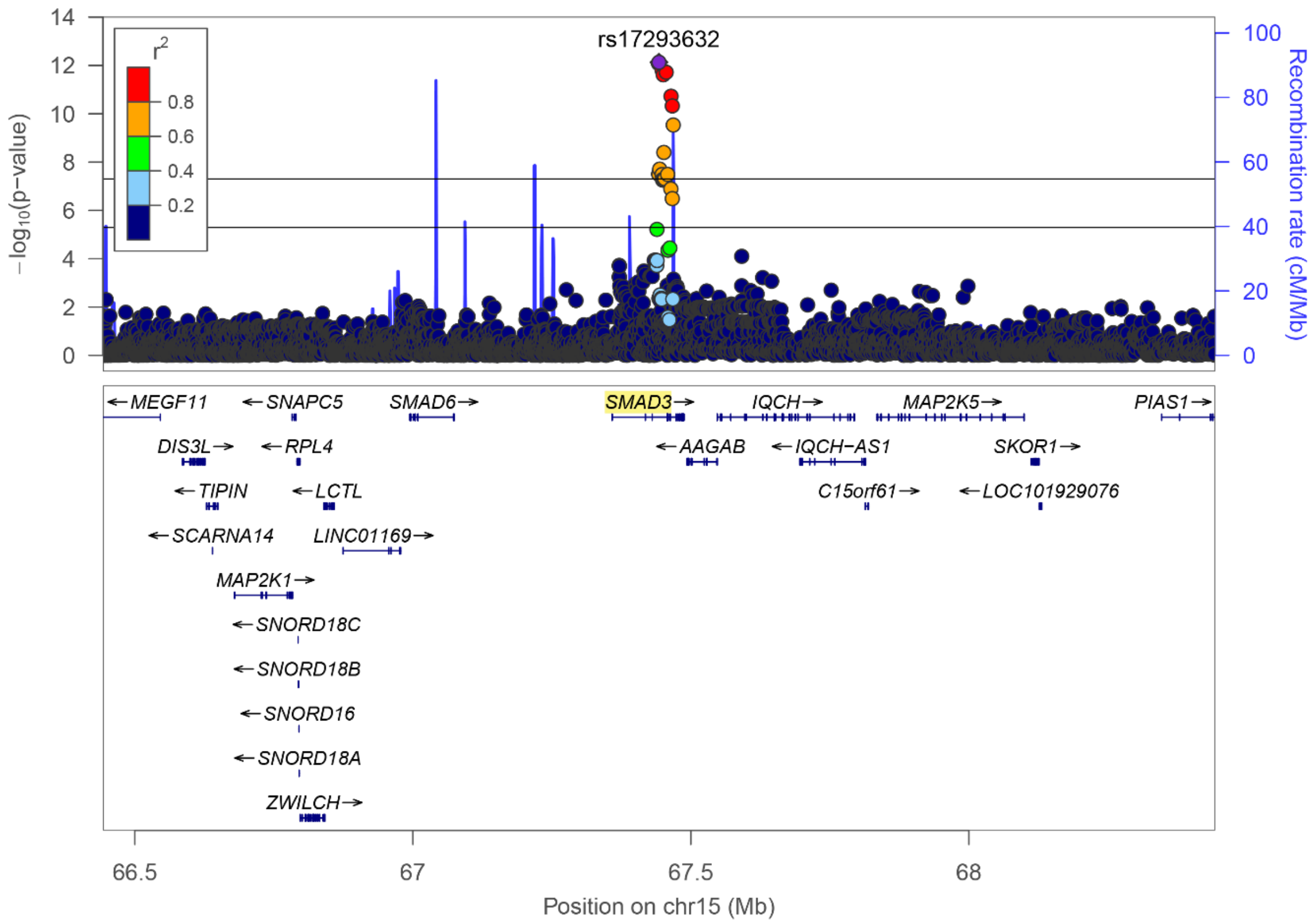
rs17293632
r2
0.8
0.6
0.4
0.2
−log10(p−value)
Recombination rate (cM/Mb)
MEGF11
SNAPC5
SMAD6
SMAD3
IQCH
MAP2K5
PIAS1
DIS3L
RPL4
AAGAB
IQCH−AS1
SKOR1
TIPIN
LCTL
C15orf61
LOC101929076
SCARNA14
LINC01169
MAP2K1
SNORD18C
SNORD18B
SNORD16
SNORD18A
ZWILCH
Position on chr15 (Mb)

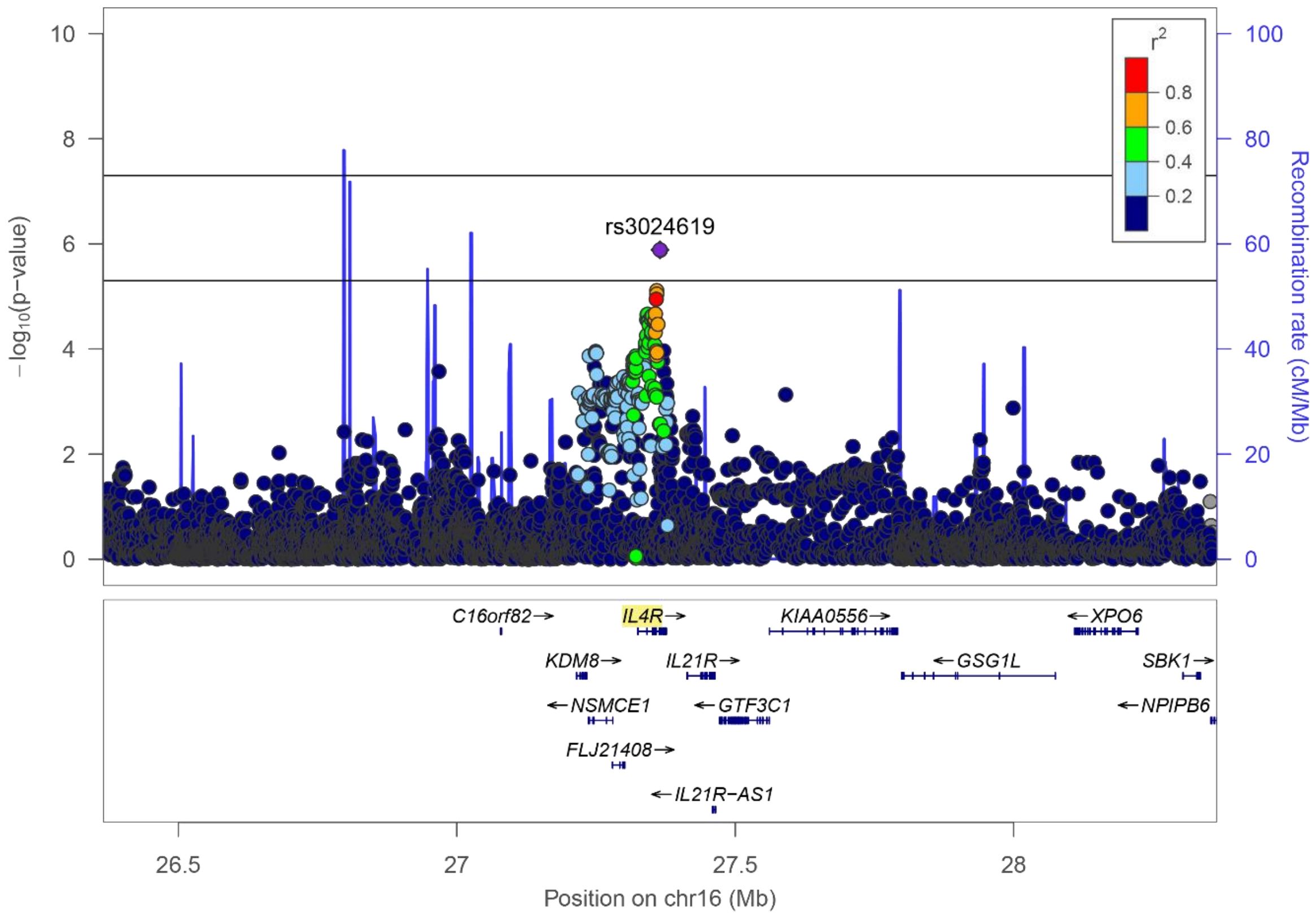
rs3024619
r2
0.8
0.6
0.4
0.2
−log10(p−value)
Recombination rate (cM/Mb)
C16orf82
IL4R
KIAA0556
XPO6
KDM8
IL21R
GSG1L
SBK1
NSMCE1
GTF3C1
NPIPB6
FLJ21408
IL21R−AS1
Position on chr16 (Mb)

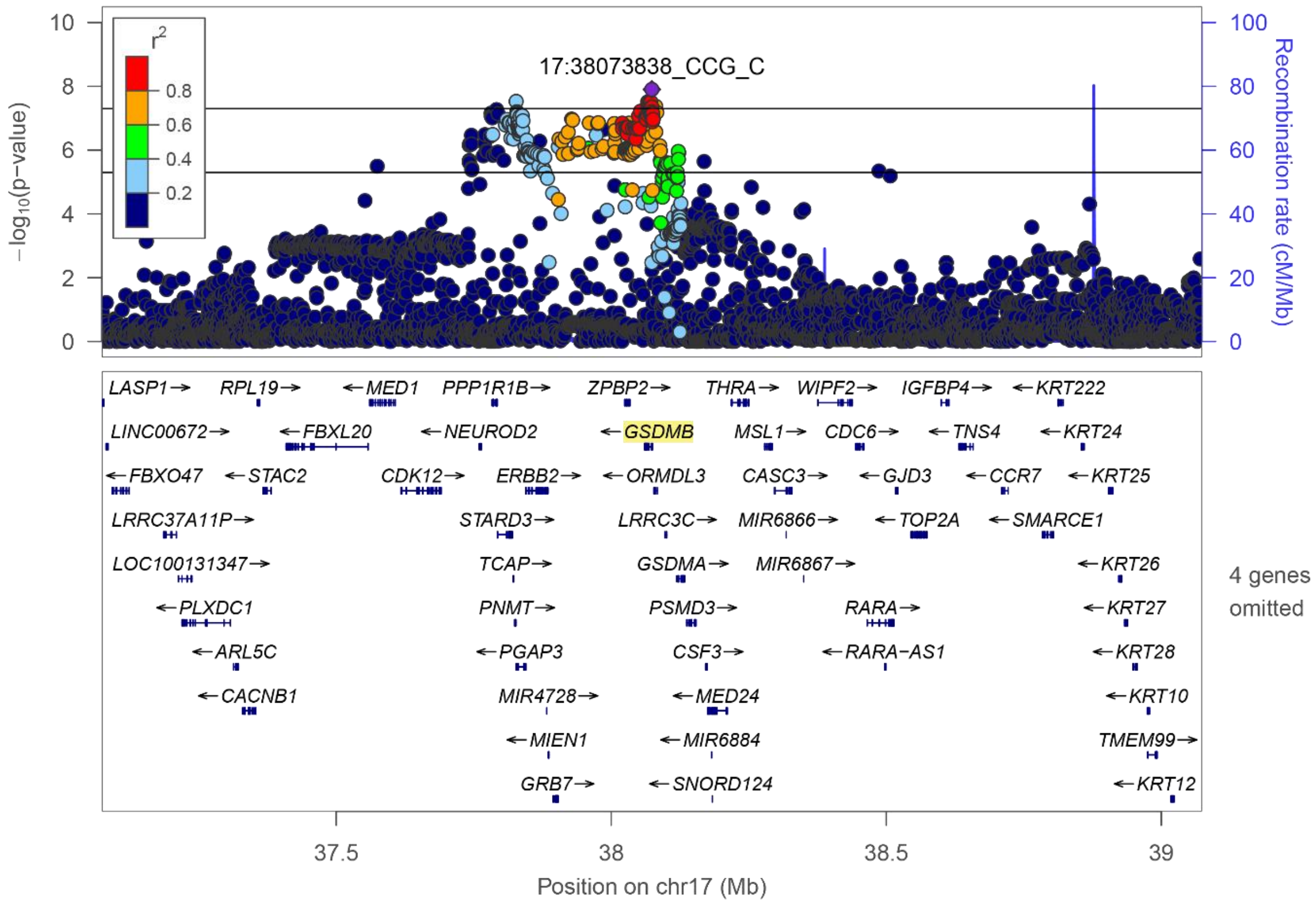

17:38073838_CCG_C
r2
0.8
0.6
0.4
0.2
−log10(p−value)
Recombination rate (cM/Mb)
LASP1
RPL19
MED1
PPP1R1B
ZPBP2
THRA
WIPF2
IGFBP4
KRT222
LINC00672
FBXL20
NEUROD2
GSDMB
MSL1
CDC6
TNS4
KRT24
FBXO47
STAC2
CDK12
ERBB2
ORMDL3
CASC3
GJD3
CCR7
KRT25
LRRC37A11P
STARD3
LRRC3C
MIR6866
TOP2A
SMARCE1
LOC100131347
TCAP
GSDMA
MIR6867
KRT26
PLXDC1
PNMT
PSMD3
RARA
KRT27
ARL5C
PGAP3
CSF3
RARA−AS1
KRT28
CACNB1
MIR4728
MED24
KRT10
MIEN1
MIR6884
TMEM99
GRB7
SNORD124
KRT12
4 genes omitted
Position on chr17 (Mb)

Figure S9

Forest plot for the 21 replicated variants. The meta-analysis summary statistics and the single cohort's summary statistics are reported in the forest plot in terms of odds ratio and 95% confidence interval. GUU = GASP, UBIOPRED, UK BIOBANK. OR = odds ratio.

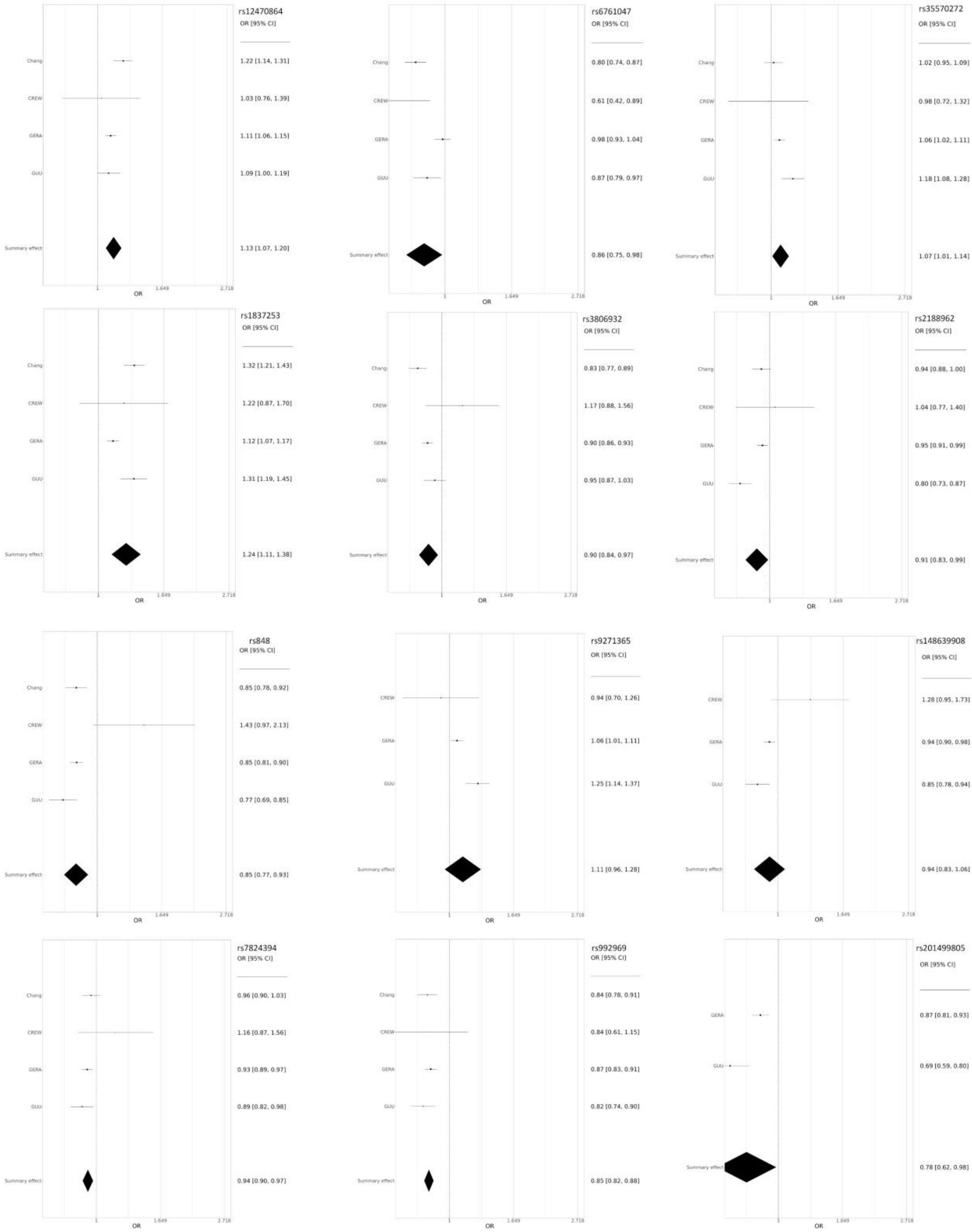

Figure S9 continues on the next page.

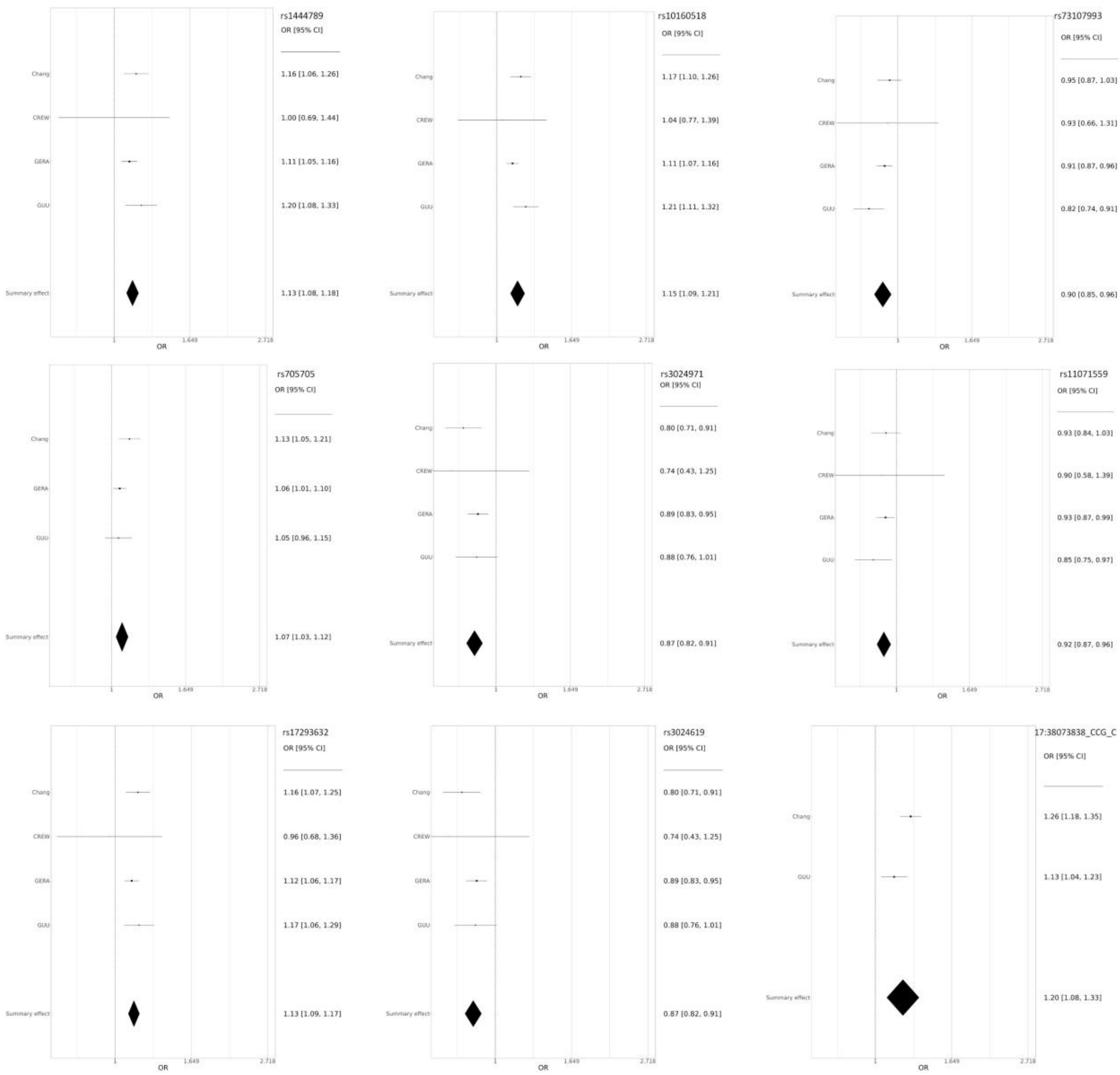

Figure S9 continued.

Figure S10

Genetic effect size comparison between discovery GWAS and European GBMI for the 21 replicated variants. Variants shows a concordance of direction of effect (A), and the module of the genetic effect size is higher in the discovery GWAS compared to European GBMI meta-analysis on asthma (A-B). The Y axis represents the log OR.

A

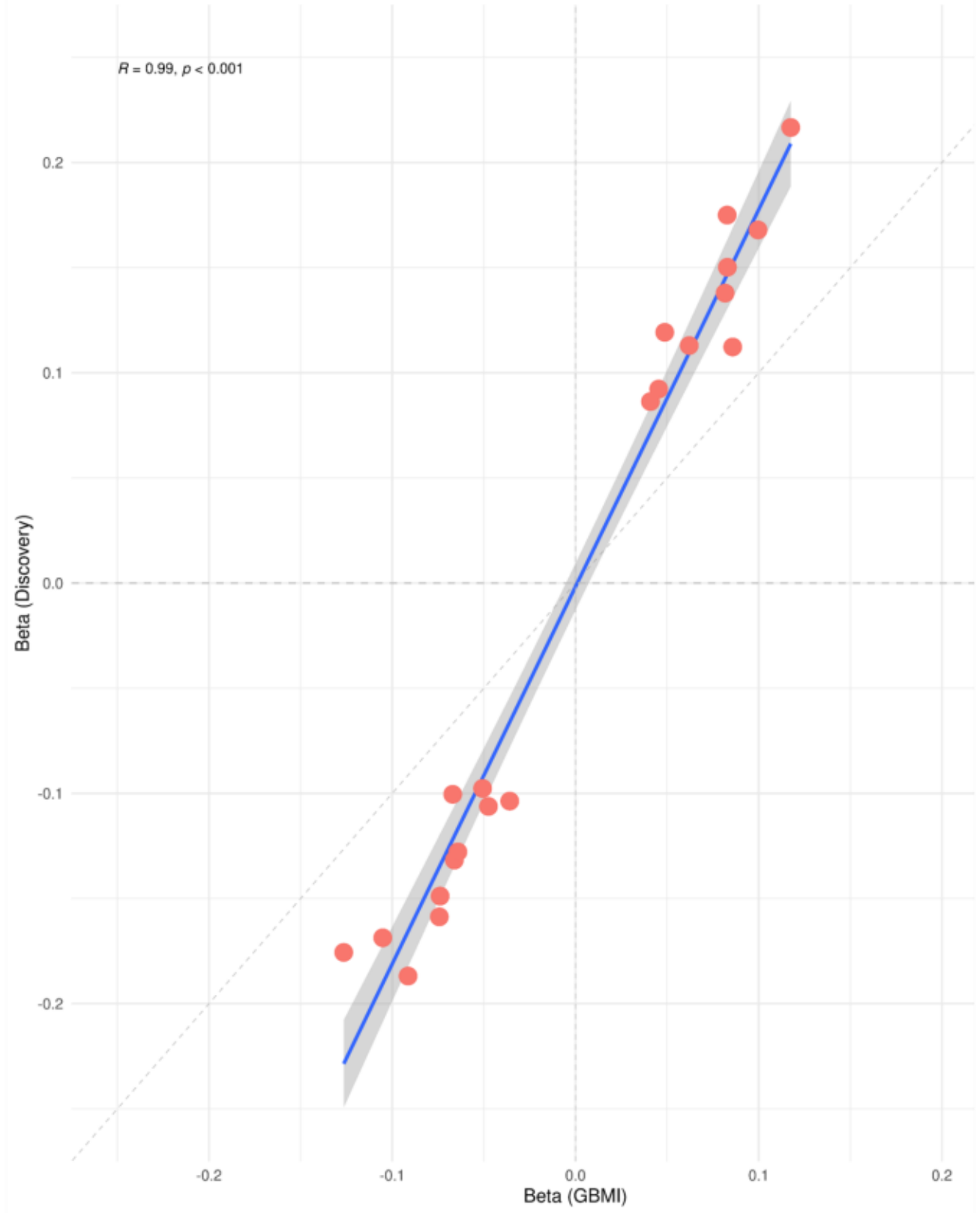

B

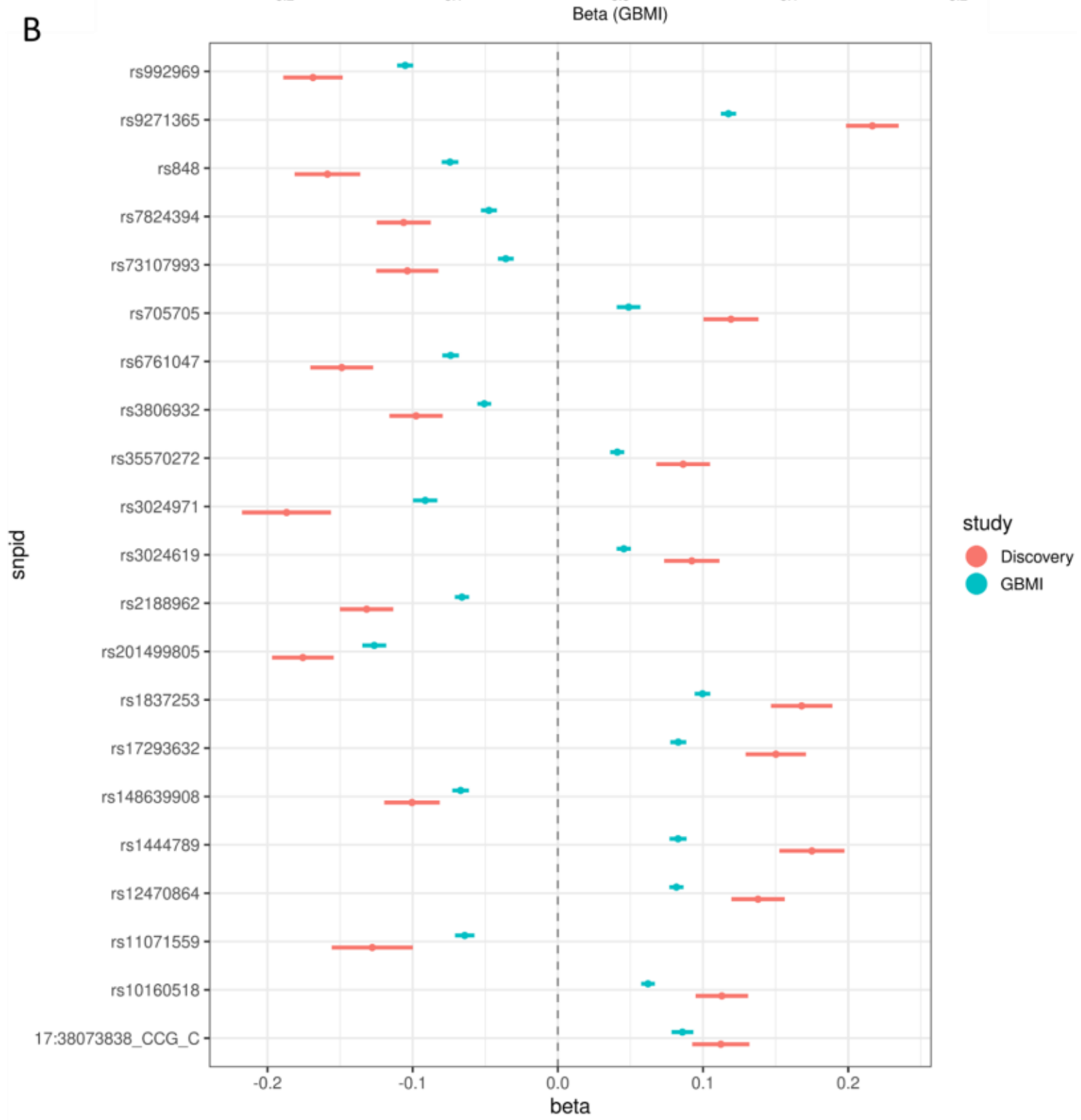

Figure S11

Comparison of effect sizes from our discovery severe asthma GWAS and the GBMI all asthma study for European cohorts. A) Correlation plot for the effect sizes of 128 index variants from European GBMI asthma against our case phenotype (Methods). Two SNPs showed significant phenotype-dependent effects (Welch's t test, two-sided, P < 3.91x10-4) and are colour-coded in vermillion: chr6:32604425:T:C, chr10:9010779:A:G. Four SNPs were replicated in the targeted meta-analysis and were colour-coded in green (chr3:33006170:T:G, chr5:111066174:C:T, chr12:57099944:G:T, chr15:67150258:T:C) (Supplementary Table S12). The Y axis represents the log OR. The error band for the regression line represents the 95% confidence interval. B) Effect size with standard error for each SNP.

A

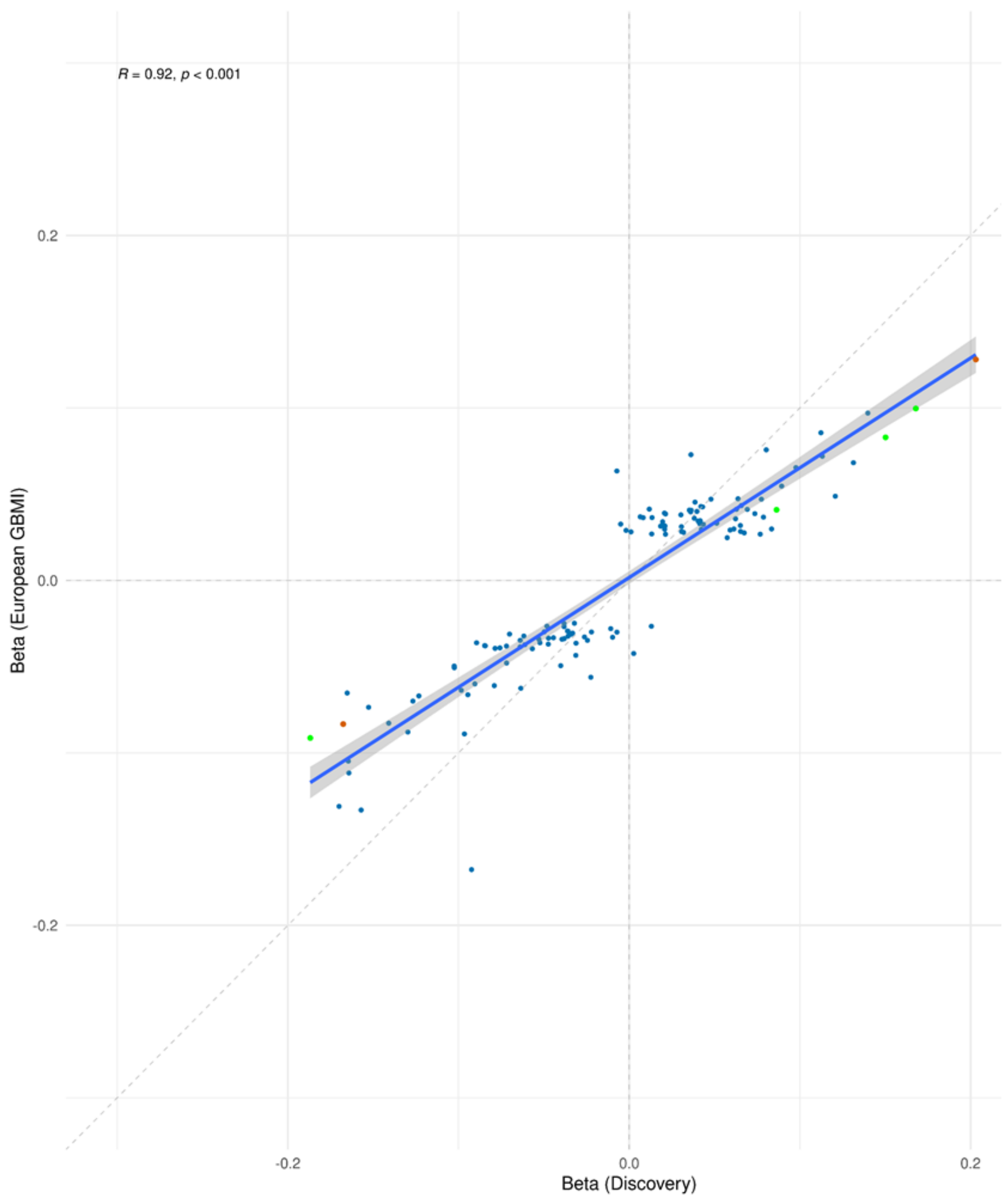

B

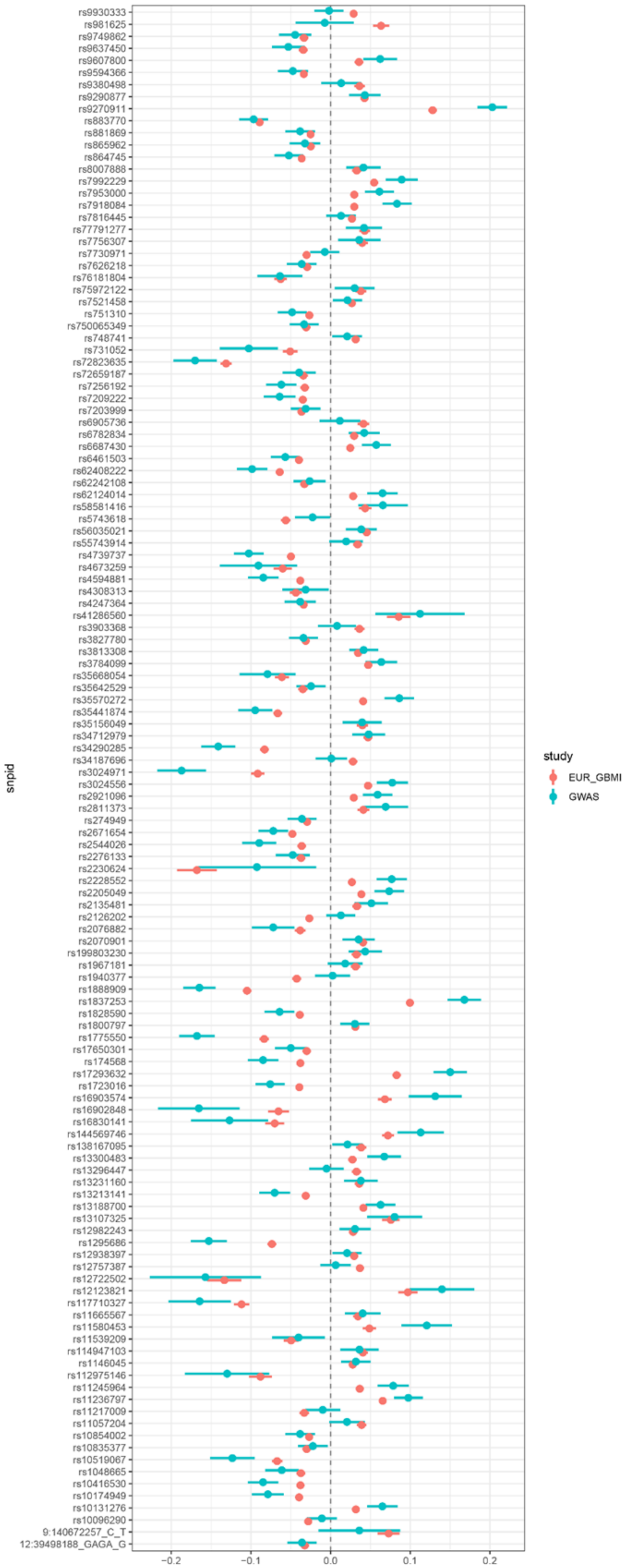

rs9930333
rs981625
rs9749862
rs9637450
rs9607800
rs9594366
rs9380498
rs9290877
rs9270911
rs883770
rs881869
rs865962
rs864745
rs8007888
rs7992229
rs7953000
rs7918084
rs7816445
rs77791277
rs7756307
rs7730971
rs7626218
rs76181804
rs75972122
rs7521458
rs751310
rs750065349
rs748741
rs731052
rs72823635
rs72659187
rs7256192
rs7209222
rs7203999
rs6905736
rs6782834
rs6687430
rs6461503
rs62408222
rs62242108
rs62124014
rs58581416
rs5743618
rs56035021
rs55743914
rs4739737
rs4673259
rs4594881
rs4308313
rs4247364
rs41286560
rs3903368
rs3827780
rs3813308
rs3784099
rs35668054
rs35642529
rs35570272
rs35441874
rs35156049
rs34712979
rs34290285
rs34187696
rs3024971
rs3024556
rs2921096
rs2811373
rs274949
rs2671654
rs2544026
rs2276133
rs2230624
rs2228552
rs2205049
rs2135481
rs2126202
rs2076882
rs2070901
rs199803230
rs1967181
rs1940377
rs1888909
rs1837253
rs1828590
rs1800797
rs1775550
rs17650301
rs174568
rs17293632
rs1723016
rs16903574
rs16902848
rs16830141
rs144569746
rs138167095
rs13300483
rs13296447
rs13231160
rs13213141
rs13188700
rs13107325
rs12982243
rs1295686
rs12938397
rs12757387
rs12722502
rs12123821
rs117710327
rs11665567
rs11580453
rs11539209
rs114947103
rs1146045
rs112975146
rs11245964
rs11236797
rs11217009
rs11057204
rs10854002
rs10835377
rs10519067
rs1048665
rs10416530
rs10174949
rs10131276
rs10096290
9:140672257_C_T
12:39498188_GAGA_G
snpid
−0.2
−0.1
0.0
0.1
0.2
beta
study
EUR_GBMI
GWAS

Figure S12

Genomic region plots of 15 credible sets from fine-mapping analysis. The represented genomic region includes a window of +/- 1Mb from the SNP with the highest posterior inclusion (PIP) probability (purple). Variants in the 95% causal credible set are filled, other variants are empty. The in-sample LD is with respect to the variant with the highest PIP.

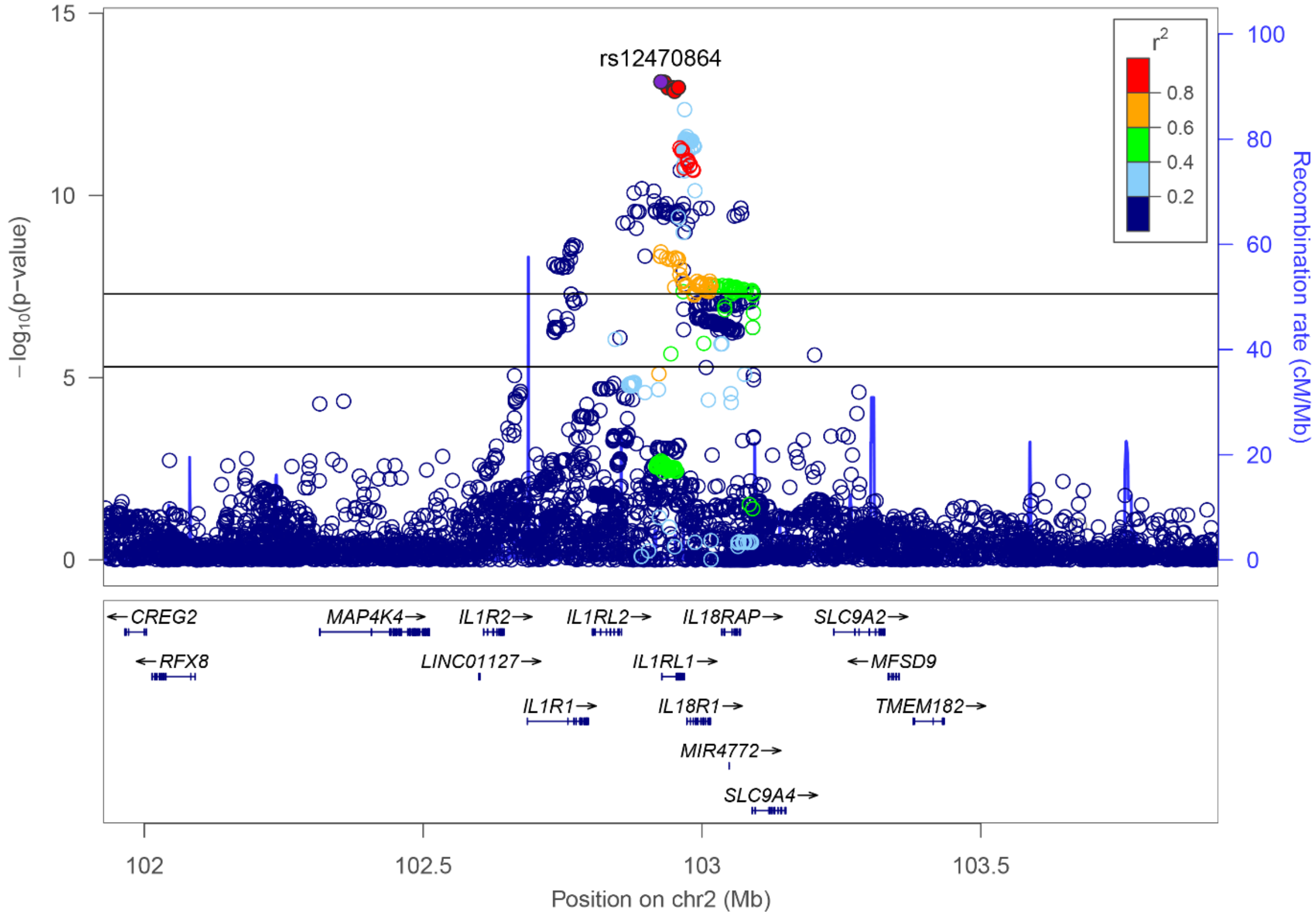

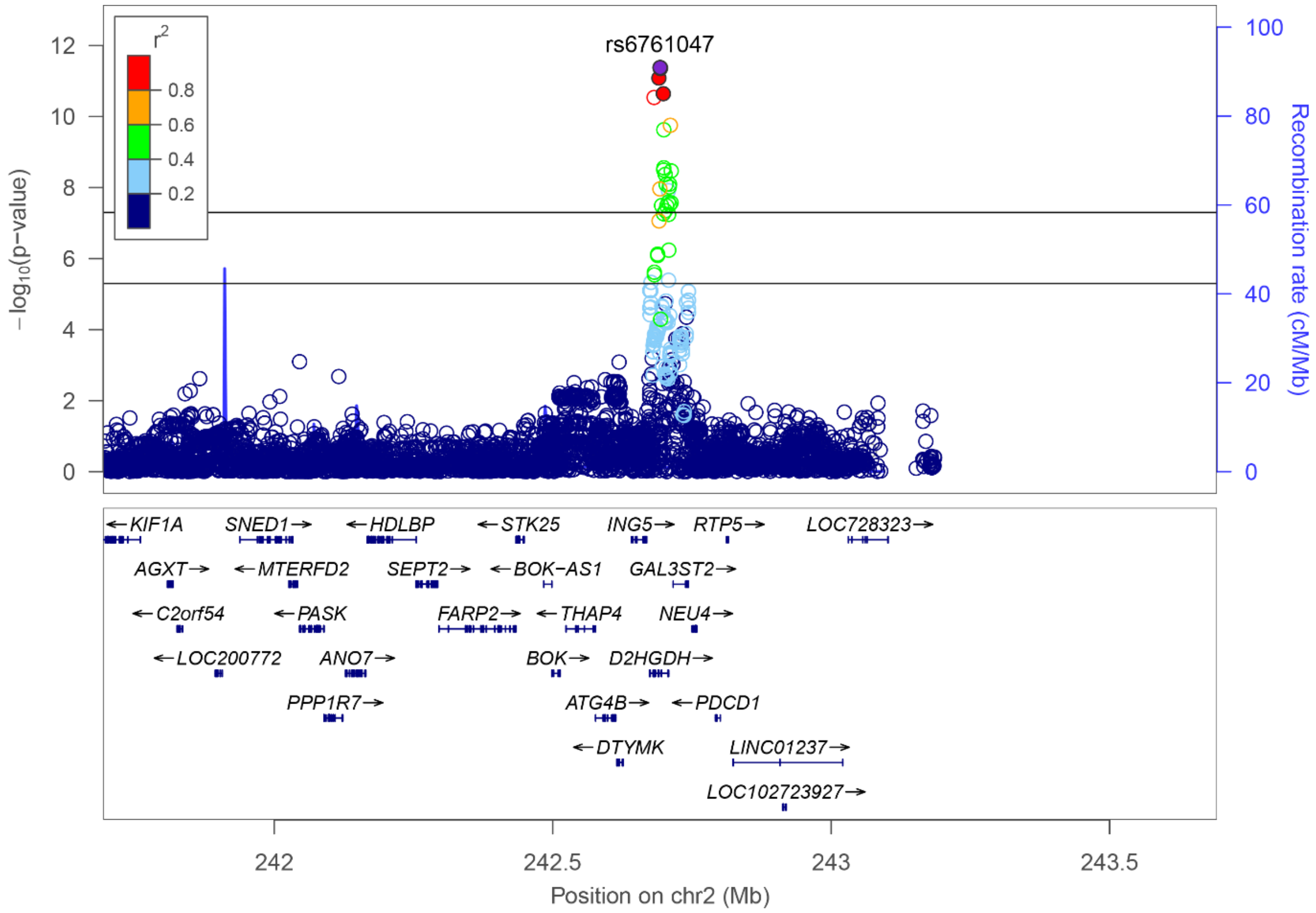
rs6761047
r2
0.8
0.6
0.4
0.2
−log10(p−value)
Recombination rate (cM/Mb)
KIF1A
SNED1
HDLBP
STK25
ING5
RTP5
LOC728323
AGXT
MTERFD2
SEPT2
BOK−AS1
GAL3ST2
C2orf54
PASK
FARP2
THAP4
NEU4
LOC200772
ANO7
BOK
D2HGDH
PPP1R7
ATG4B
PDCD1
DTYMK
LINC01237
LOC102723927
242
242.5
243
243.5
Position on chr2 (Mb)

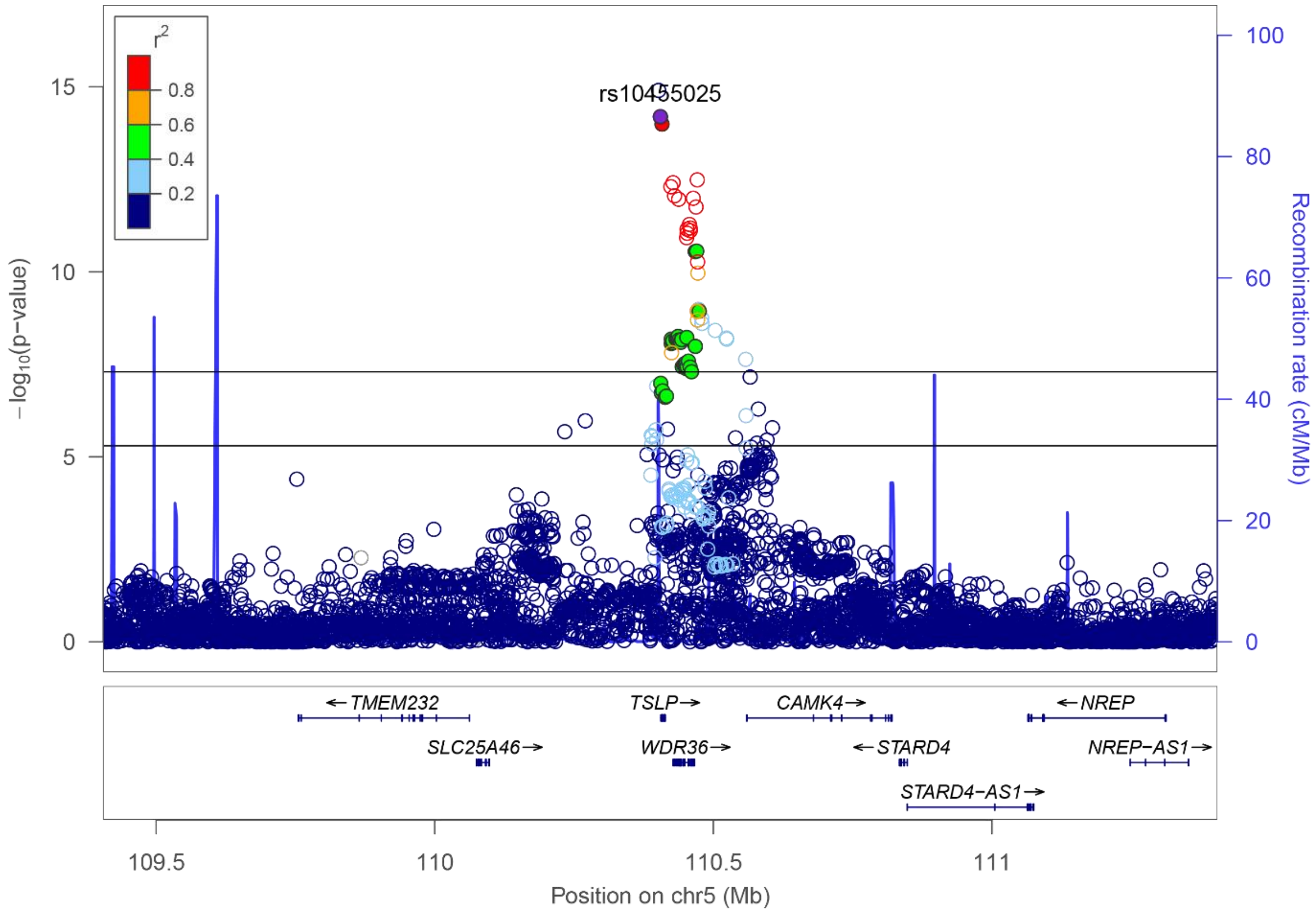
rs10455025
r2
0.8
0.6
0.4
0.2
−log10(p−value)
Recombination rate (cM/Mb)
TMEM232
TSLP
CAMK4
NREP
SLC25A46
WDR36
STARD4
NREP−AS1
STARD4−AS1
109.5
110
110.5
111
Position on chr5 (Mb)

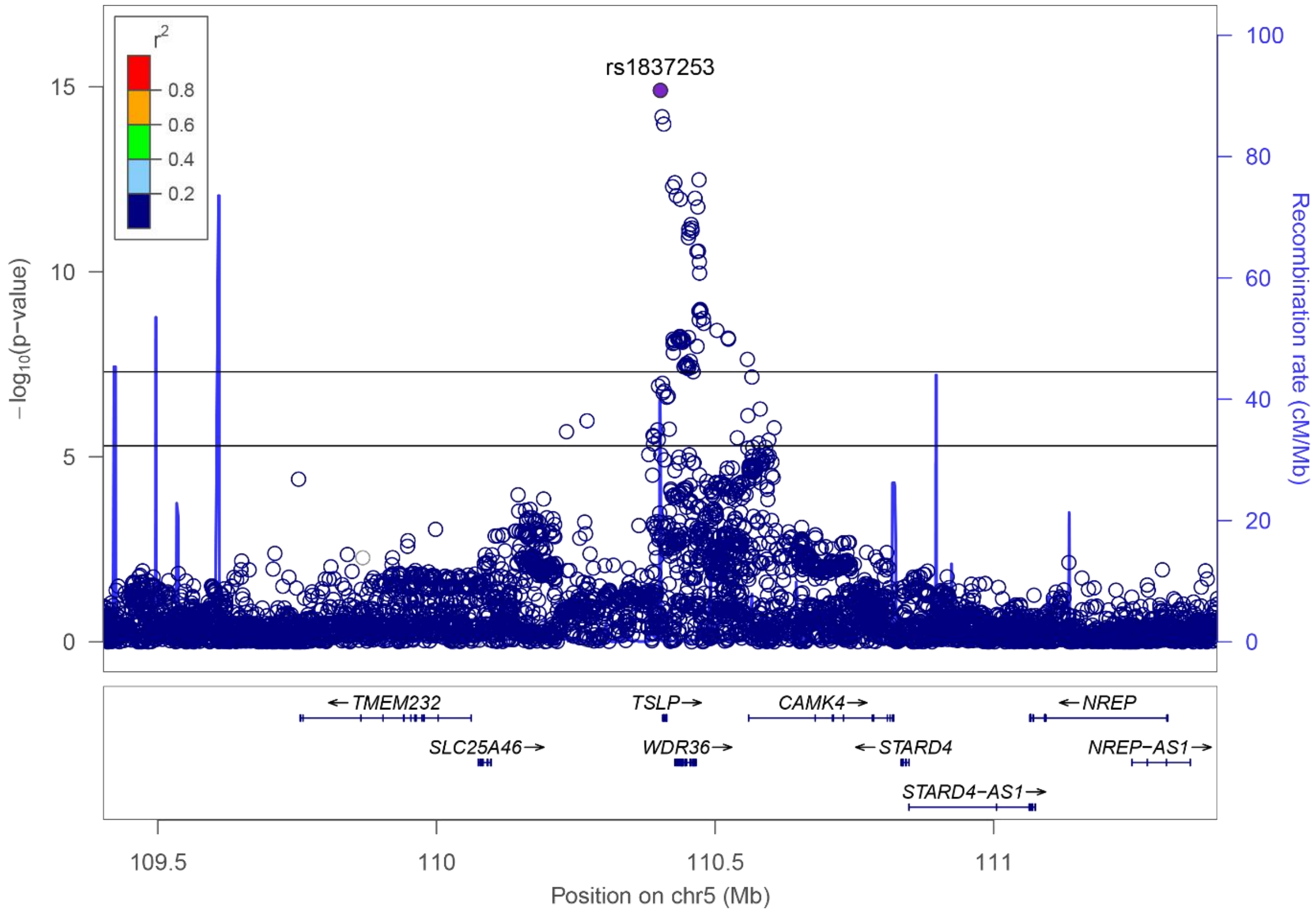
rs1837253
r2
0.8
0.6
0.4
0.2
−log10(p−value)
Recombination rate (cM/Mb)
TMEM232
TSLP
CAMK4
NREP
SLC25A46
WDR36
STARD4
NREP−AS1
STARD4−AS1
109.5
110
110.5
111
Position on chr5 (Mb)

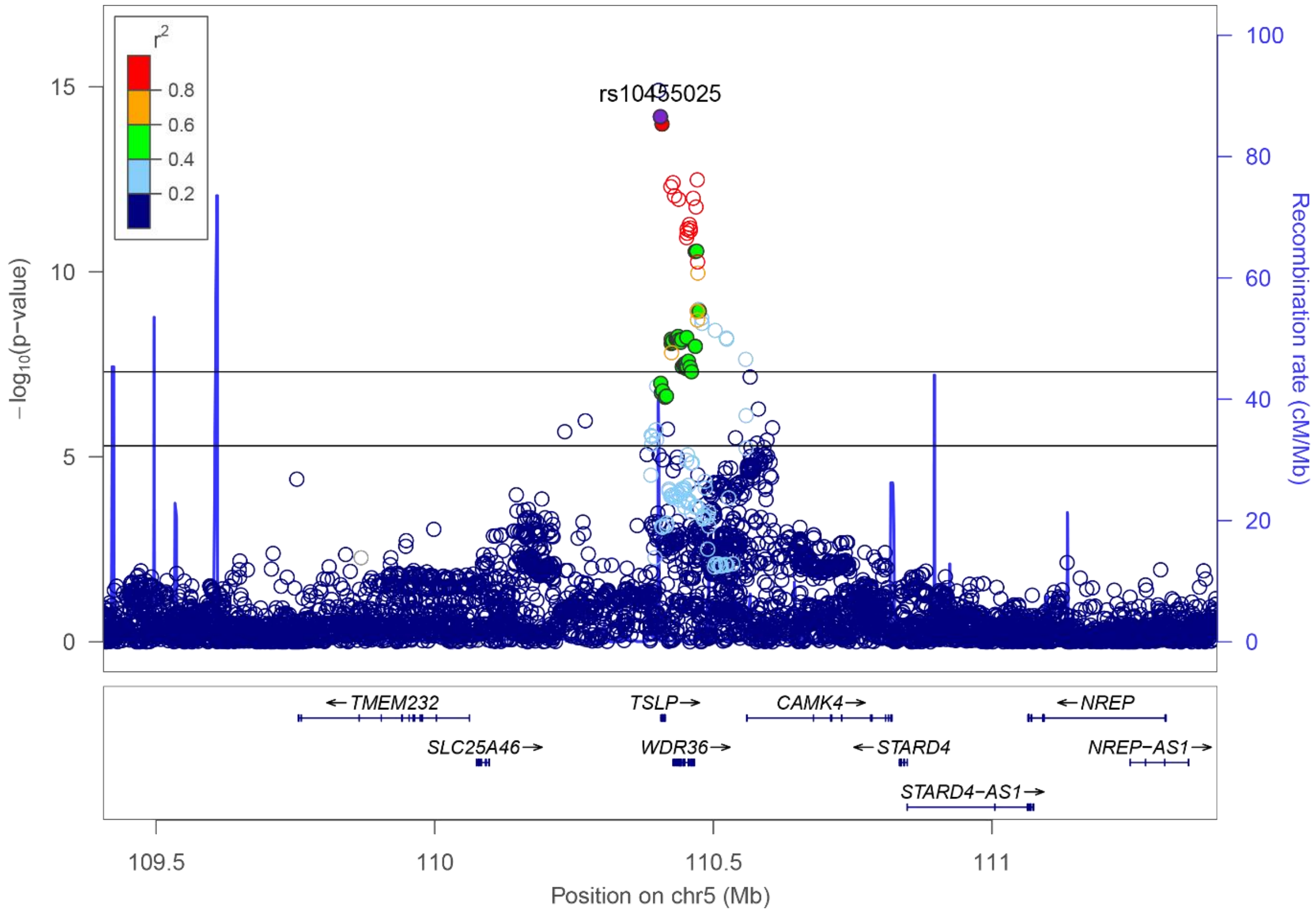
rs10455025
r2
0.8
0.6
0.4
0.2
−log10(p−value)
Recombination rate (cM/Mb)
TMEM232
TSLP
CAMK4
NREP
SLC25A46
WDR36
STARD4
NREP−AS1
STARD4−AS1
109.5
110
110.5
111
Position on chr5 (Mb)

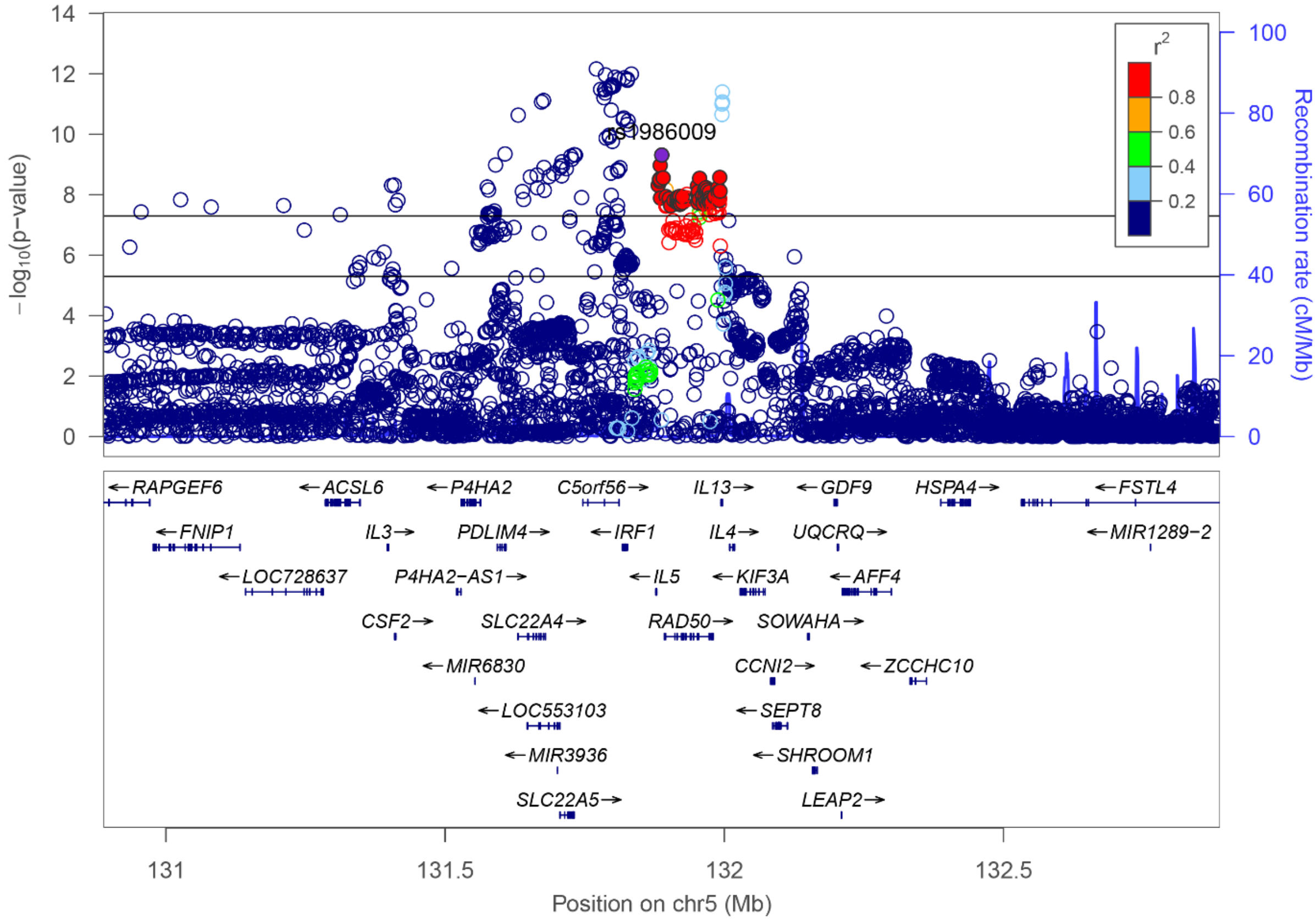
rs1986009
−log10(p-value)
Recombination rate (cM/Mb)
r2
0.8
0.6
0.4
0.2
RAPGEF6
ACSL6
P4HA2
C5orf56
IL13
GDF9
HSPA4
FSTL4
FNIP1
IL3
PDLIM4
IRF1
IL4
UQCRQ
MIR1289−2
LOC728637
P4HA2−AS1
IL5
KIF3A
AFF4
CSF2
SLC22A4
RAD50
SOWAHA
MIR6830
CCNI2
ZCCHC10
LOC553103
SEPT8
MIR3936
SHROOM1
SLC22A5
LEAP2
Position on chr5 (Mb)

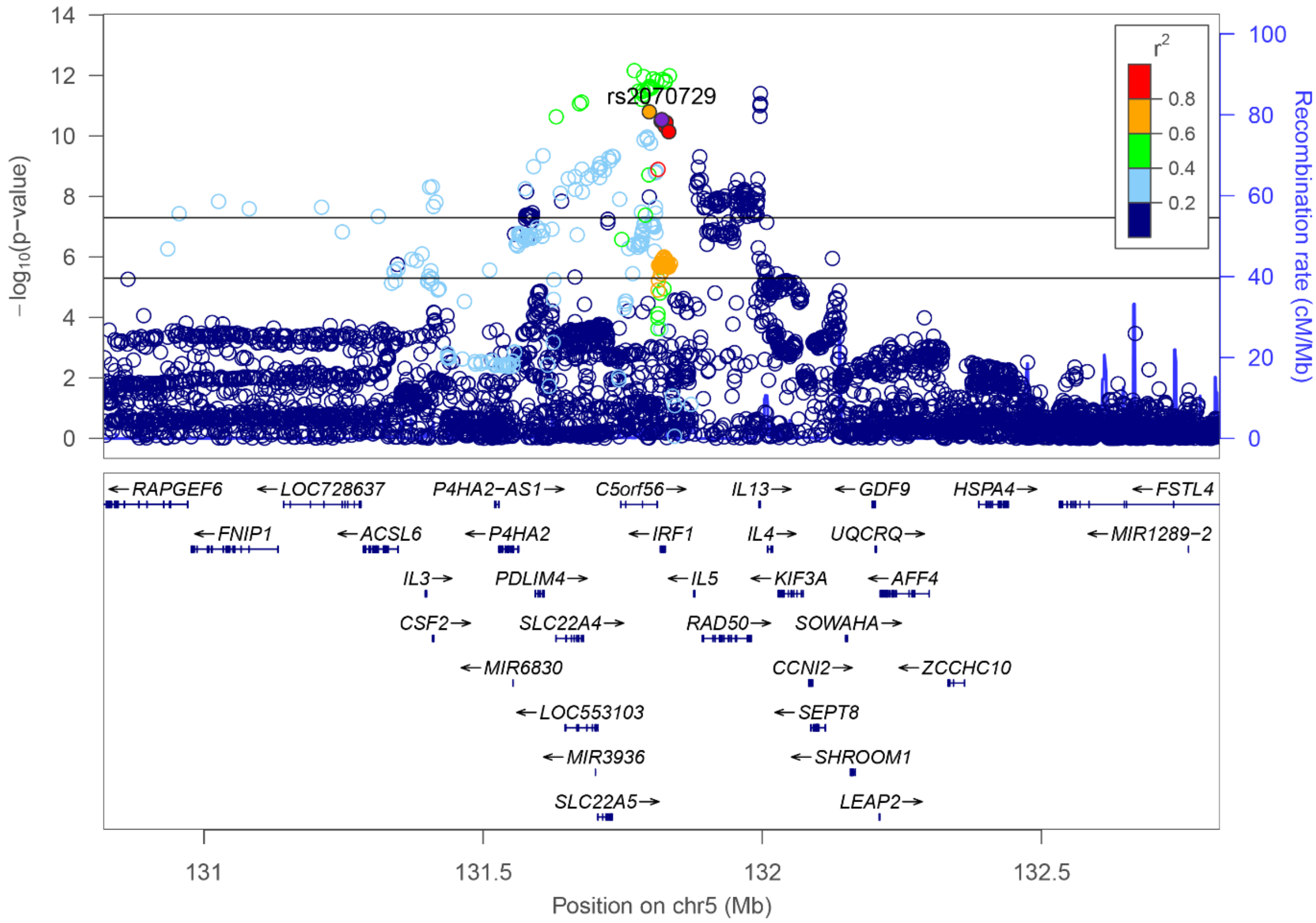
rs2070729
−log10(p-value)
Recombination rate (cM/Mb)
r2
0.8
0.6
0.4
0.2
RAPGEF6
LOC728637
P4HA2−AS1
C5orf56
IL13
GDF9
HSPA4
FSTL4
FNIP1
ACSL6
P4HA2
IRF1
IL4
UQCRQ
MIR1289−2
IL3
PDLIM4
IL5
KIF3A
AFF4
CSF2
SLC22A4
RAD50
SOWAHA
MIR6830
CCNI2
ZCCHC10
LOC553103
SEPT8
MIR3936
SHROOM1
SLC22A5
LEAP2
Position on chr5 (Mb)

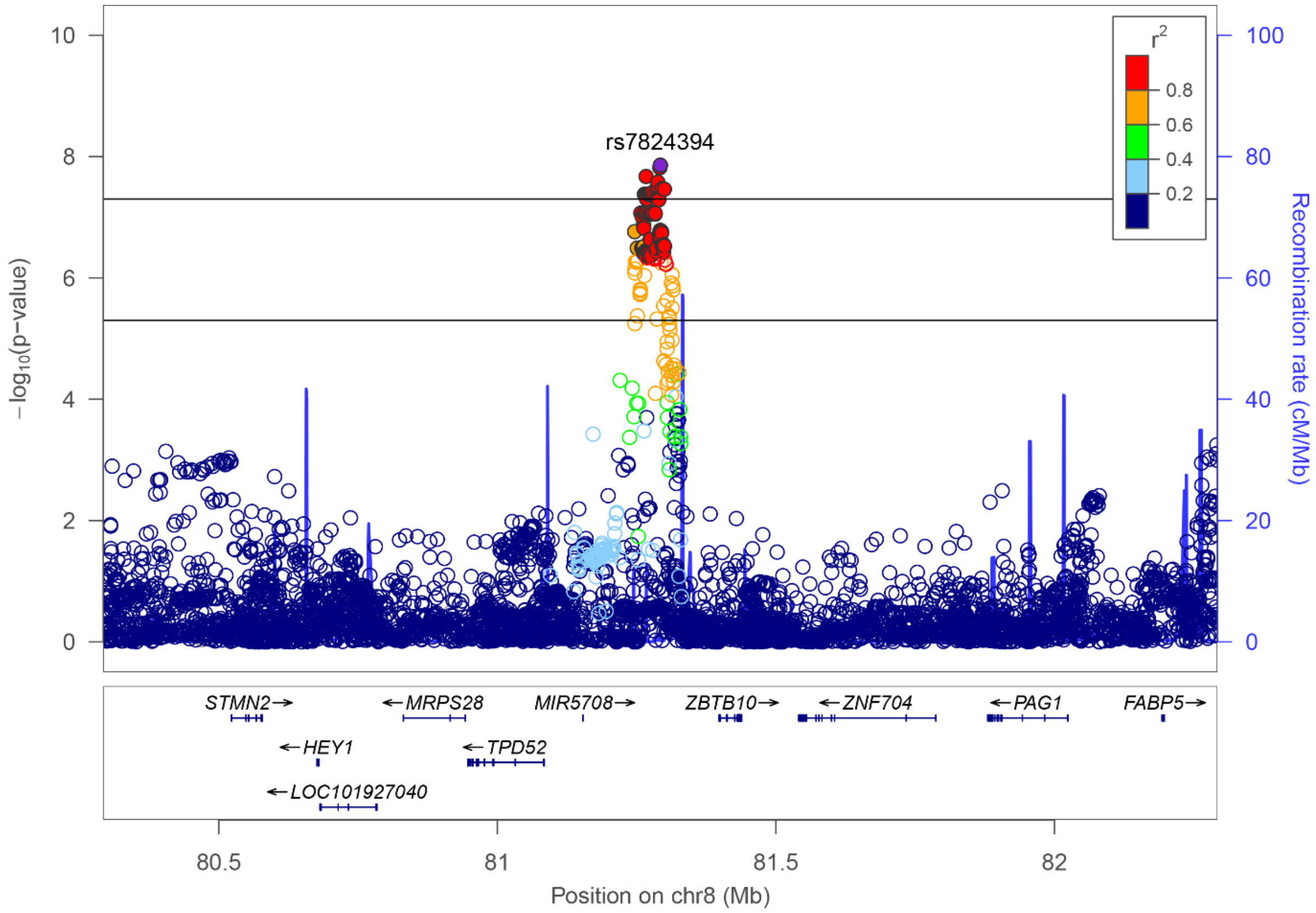

rs7824394
r2
0.8
0.6
0.4
0.2
−log10(p−value)
Recombination rate (cM/Mb)
STMN2→
←MRPS28
MIR5708→
ZBTB10→
←ZNF704
←PAG1
FABP5→
←HEY1
←TPD52
←LOC101927040
Position on chr8 (Mb)

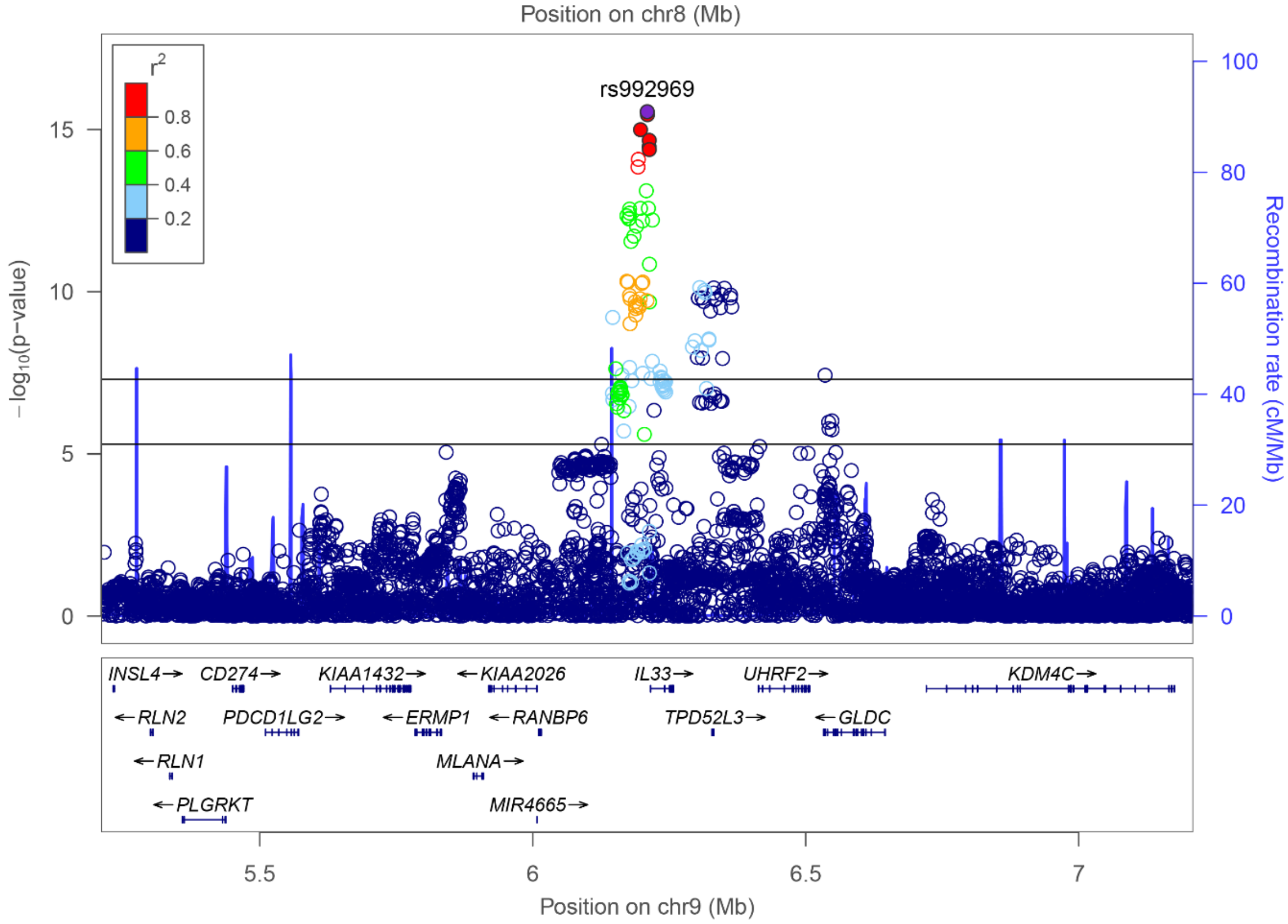

rs992969
r2
0.8
0.6
0.4
0.2
−log10(p−value)
Recombination rate (cM/Mb)
INSL4→
CD274→
KIAA1432→
←KIAA2026
IL33→
UHRF2→
KDM4C→
←RLN2
PDCD1LG2→
←ERMP1
←RANBP6
TPD52L3→
←GLDC
←RLN1
MLANA→
←PLGRKT
MIR4665→
Position on chr9 (Mb)

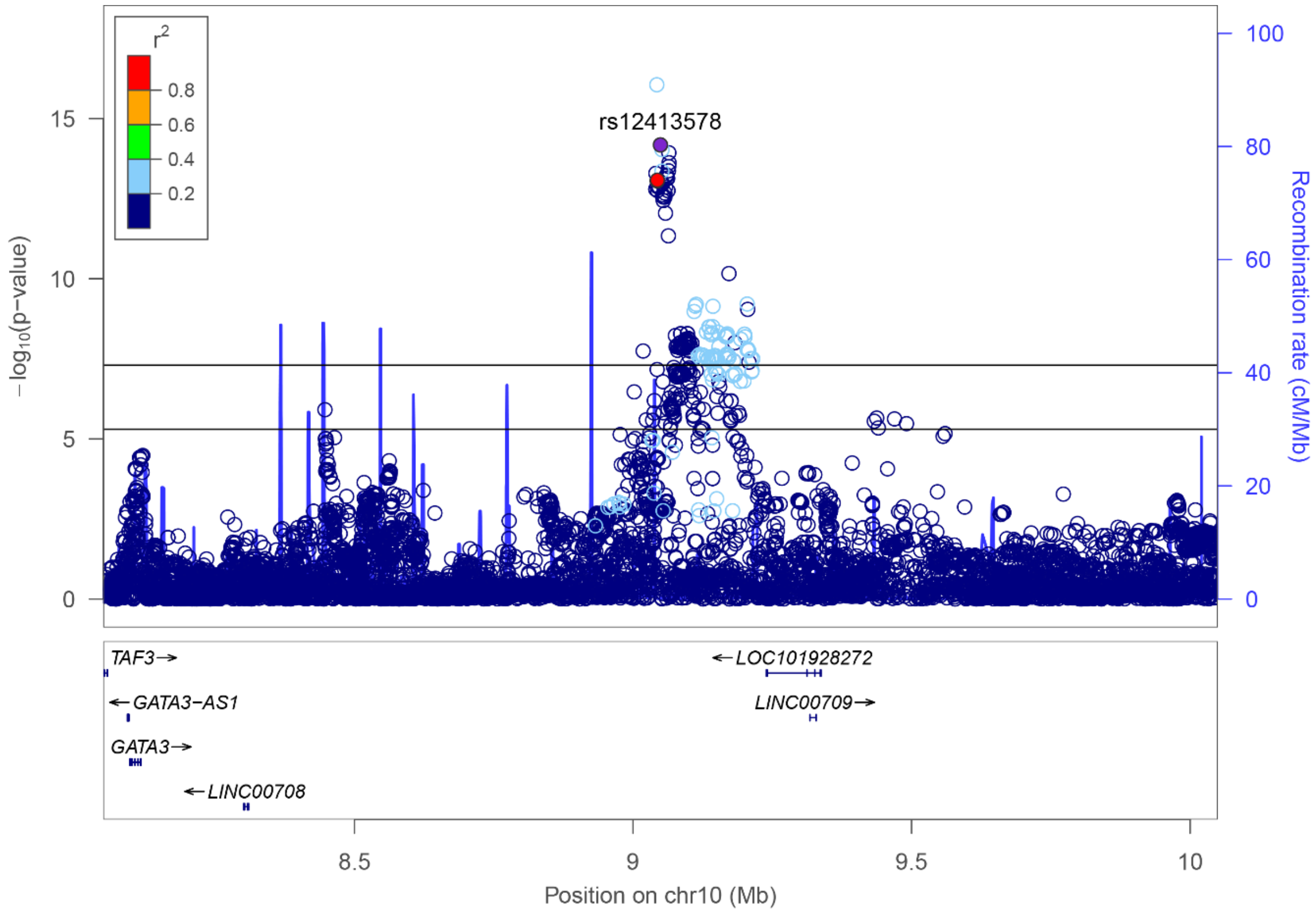

r2
0.8
0.6
0.4
0.2
rs12413578
−log10(p−value)
Recombination rate (cM/Mb)
TAF3
GATA3−AS1
GATA3
LINC00708
LOC101928272
LINC00709
Position on chr10 (Mb)

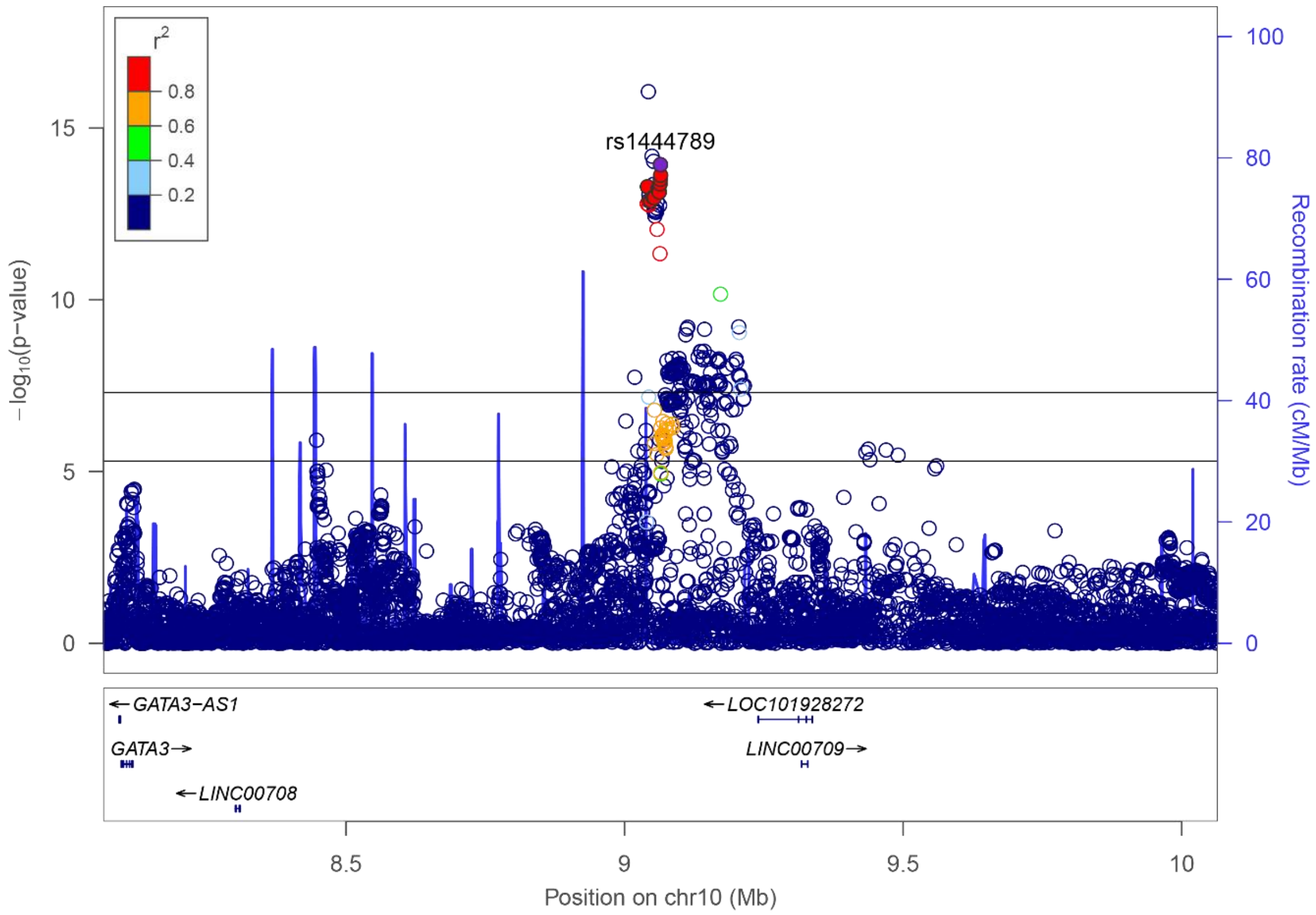

r2
0.8
0.6
0.4
0.2
rs1444789
−log10(p−value)
Recombination rate (cM/Mb)
GATA3−AS1
GATA3
LINC00708
LOC101928272
LINC00709
Position on chr10 (Mb)

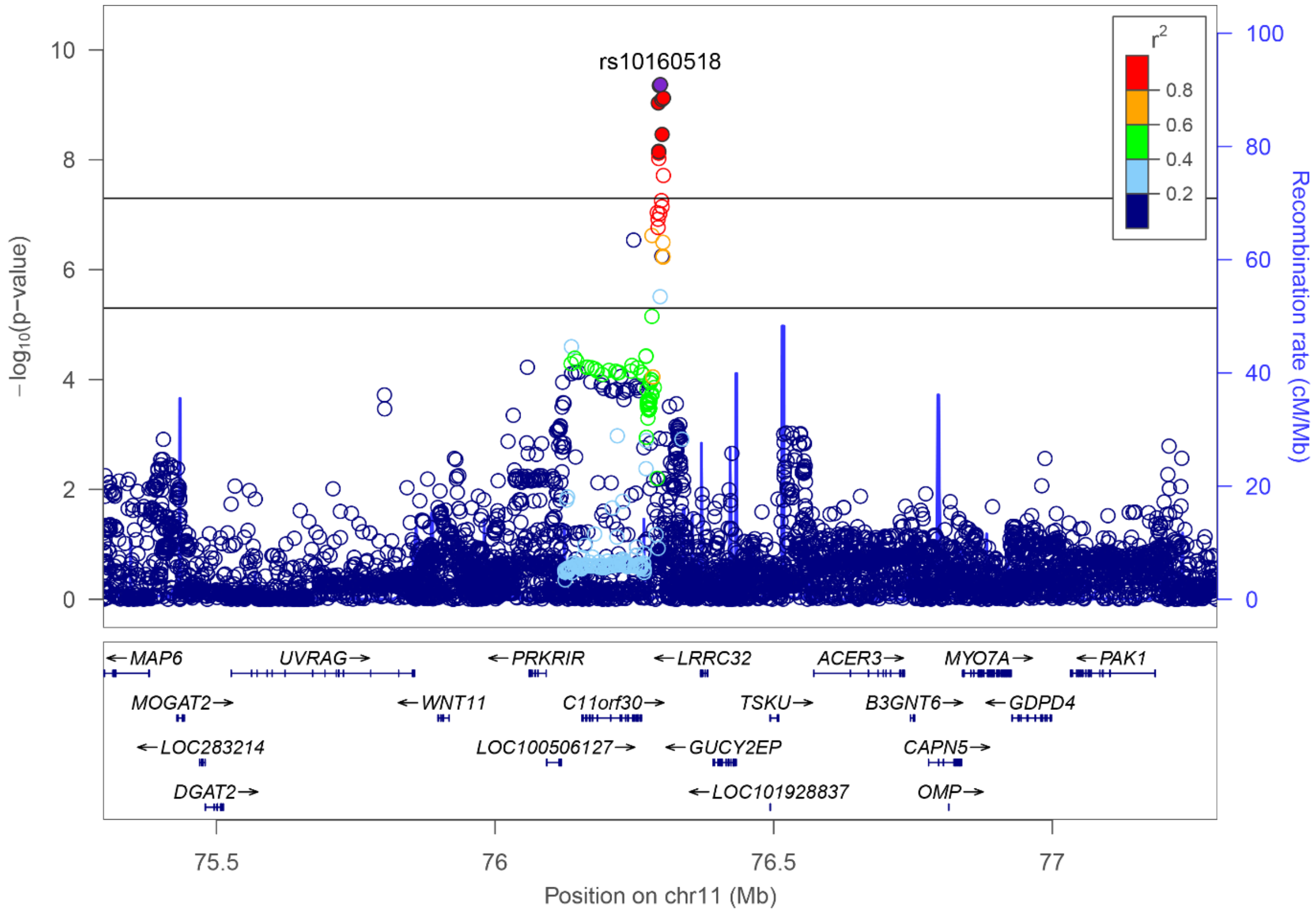
rs10160518
−log10(p-value)
Recombination rate (cM/Mb)
r2
0.8
0.6
0.4
0.2
10
8
6
4
2
0
100
80
60
40
20
MAP6
UVRAG
PRKRIR
LRRC32
ACER3
MYO7A
PAK1
MOGAT2
WNT11
C11orf30
TSKU
B3GNT6
GDPD4
LOC283214
LOC100506127
GUCY2EP
CAPN5
DGAT2
LOC101928837
OMP
75.5
76
76.5
77
Position on chr11 (Mb)

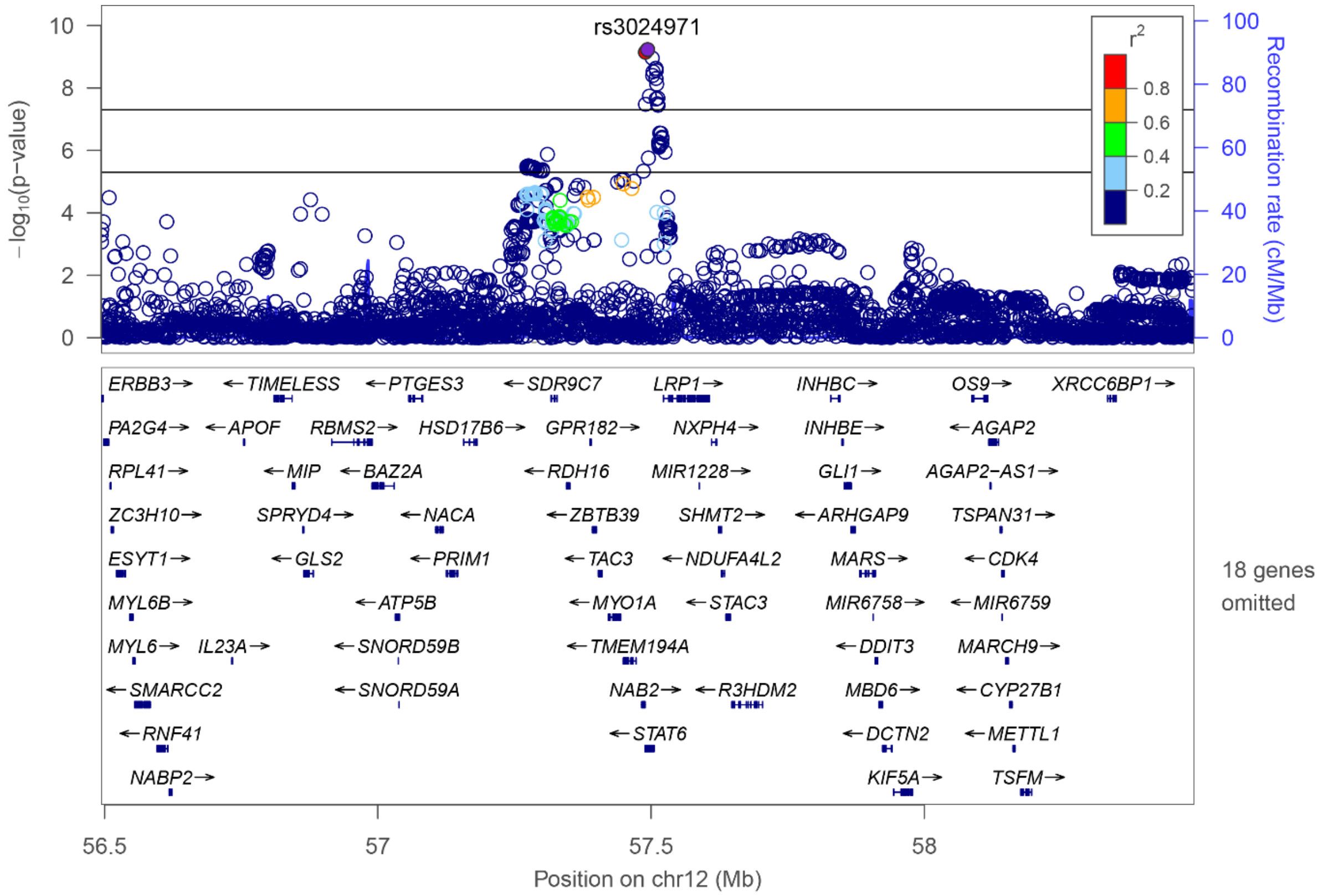
rs3024971
−log10(p-value)
Recombination rate (cM/Mb)
r2
0.8
0.6
0.4
0.2
10
8
6
4
2
0
100
80
60
40
20
ERBB3
TIMELESS
PTGES3
SDR9C7
LRP1
INHBC
OS9
XRCC6BP1
PA2G4
APOF
RBMS2
HSD17B6
GPR182
NXPH4
INHBE
AGAP2
RPL41
MIP
BAZ2A
RDH16
MIR1228
GLI1
AGAP2−AS1
ZC3H10
SPRYD4
NACA
ZBTB39
SHMT2
ARHGAP9
TSPAN31
ESYT1
GLS2
PRIM1
TAC3
NDUFA4L2
MARS
CDK4
MYL6B
ATP5B
MYO1A
STAC3
MIR6758
MIR6759
MYL6
IL23A
SNORD59B
TMEM194A
DDIT3
MARCH9
SMARCC2
SNORD59A
NAB2
R3HDM2
MBD6
CYP27B1
RNF41
STAT6
DCTN2
METTL1
NABP2
KIF5A
TSFM
18 genes omitted
56.5
57
57.5
58
Position on chr12 (Mb)

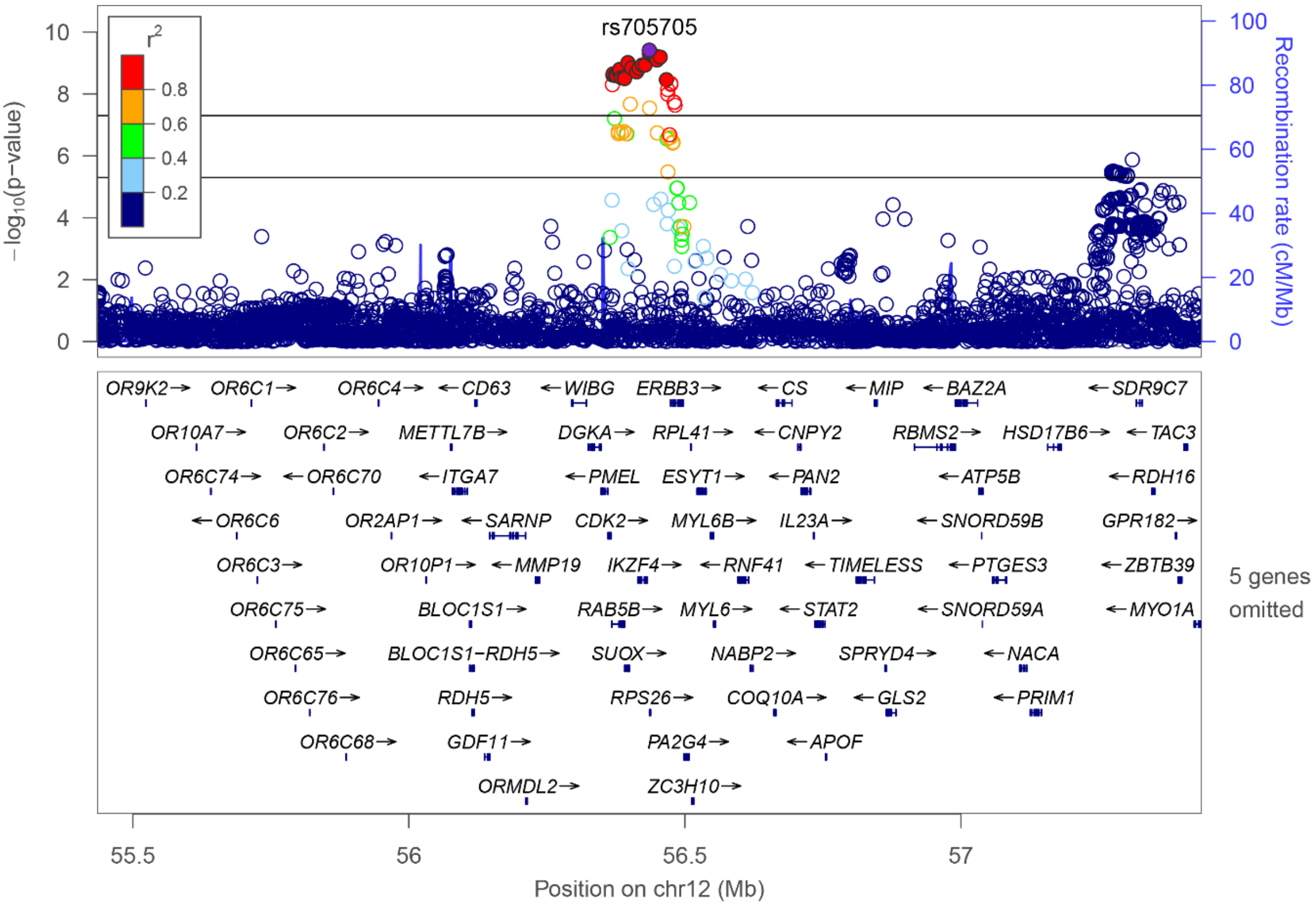
rs705705
r2
0.8
0.6
0.4
0.2
−log10(p-value)
Recombination rate (cM/Mb)
OR9K2 OR6C1 OR6C4 CD63 WIBG ERBB3 CS MIP BAZ2A SDR9C7
OR10A7 OR6C2 METTL7B DGKA RPL41 CNPY2 RBMS2 HSD17B6 TAC3
OR6C74 OR6C70 ITGA7 PMEL ESYT1 PAN2 ATP5B RDH16
OR6C6 OR2AP1 SARNP CDK2 MYL6B IL23A SNORD59B GPR182
OR6C3 OR10P1 MMP19 IKZF4 RNF41 TIMELESS PTGES3 ZBTB39
OR6C75 BLOC1S1 RAB5B MYL6 STAT2 SNORD59A MYO1A
OR6C65 BLOC1S1−RDH5 SUOX NABP2 SPRYD4 NACA
OR6C76 RDH5 RPS26 COQ10A GLS2 PRIM1
OR6C68 GDF11 PA2G4 APOF
ORMDL2 ZC3H10
5 genes omitted
55.5
56
56.5
57
Position on chr12 (Mb)

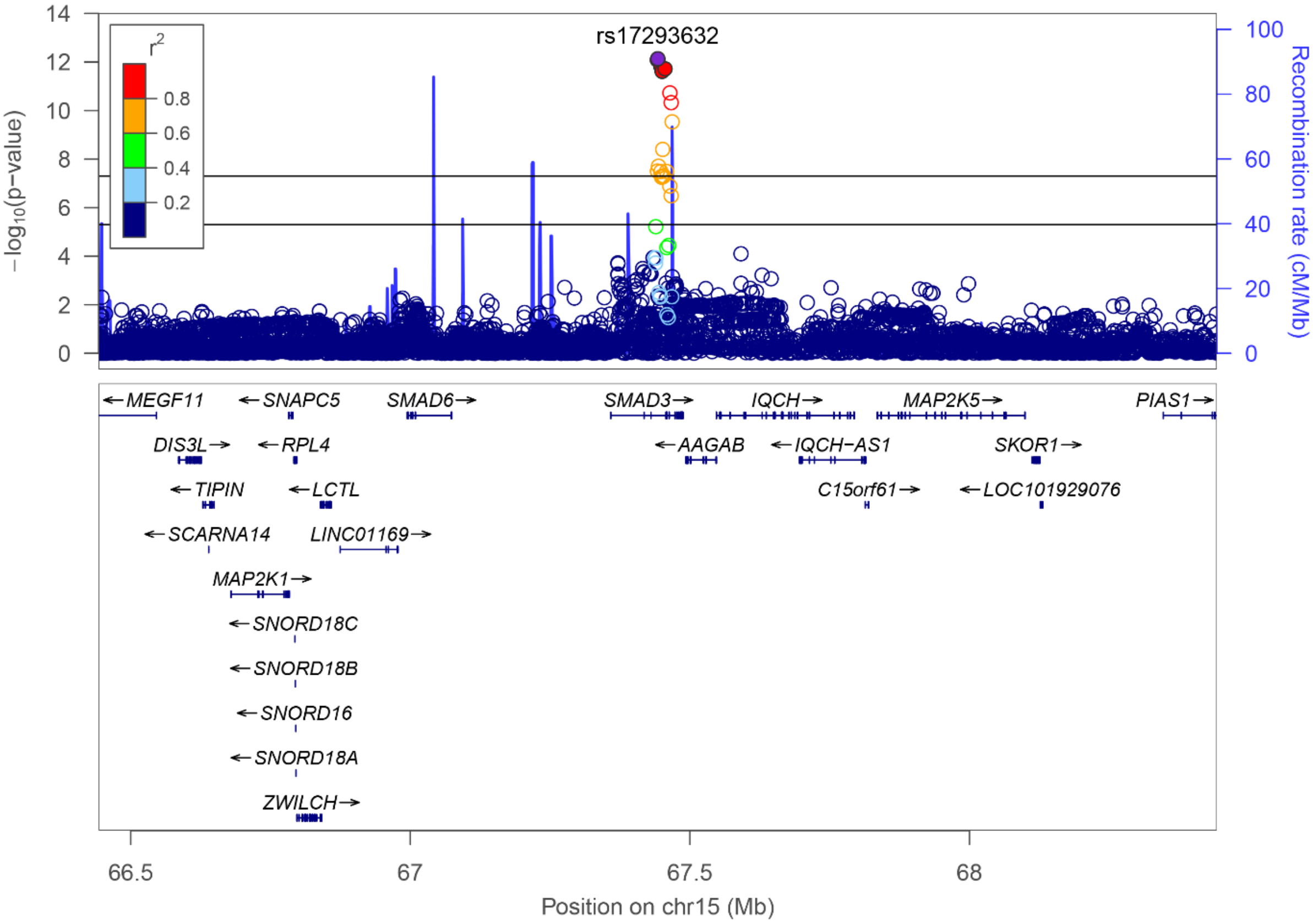
rs17293632
r2
0.8
0.6
0.4
0.2
−log10(p-value)
Recombination rate (cM/Mb)
MEGF11 SNAPC5 SMAD6 SMAD3 IQCH MAP2K5 PIAS1
DIS3L RPL4 AAGAB IQCH−AS1 SKOR1
TIPIN LCTL C15orf61 LOC101929076
SCARNA14 LINC01169
MAP2K1
SNORD18C
SNORD18B
SNORD16
SNORD18A
ZWILCH
66.5
67
67.5
68
Position on chr15 (Mb)

Figure S13

STRING protein-protein interaction network for 85 prioritised identified genes. Each sphere is a protein corresponding to the investigated genes. Each node between two proteins represents a predicted association. Two proteins can be connected by multiple lines where the colour identifies the type of interaction evidence. Evidence of interaction included seven sources: textmining (light green), experiments (purple), curated databases (light blue), gene co-expression (black), neighbourhood, gene fusion (red), and gene co-occurrence (blue). Confidence of the interaction score was setting at 0.4. ENSP00000491596 is protein mapped to AC116366.3.

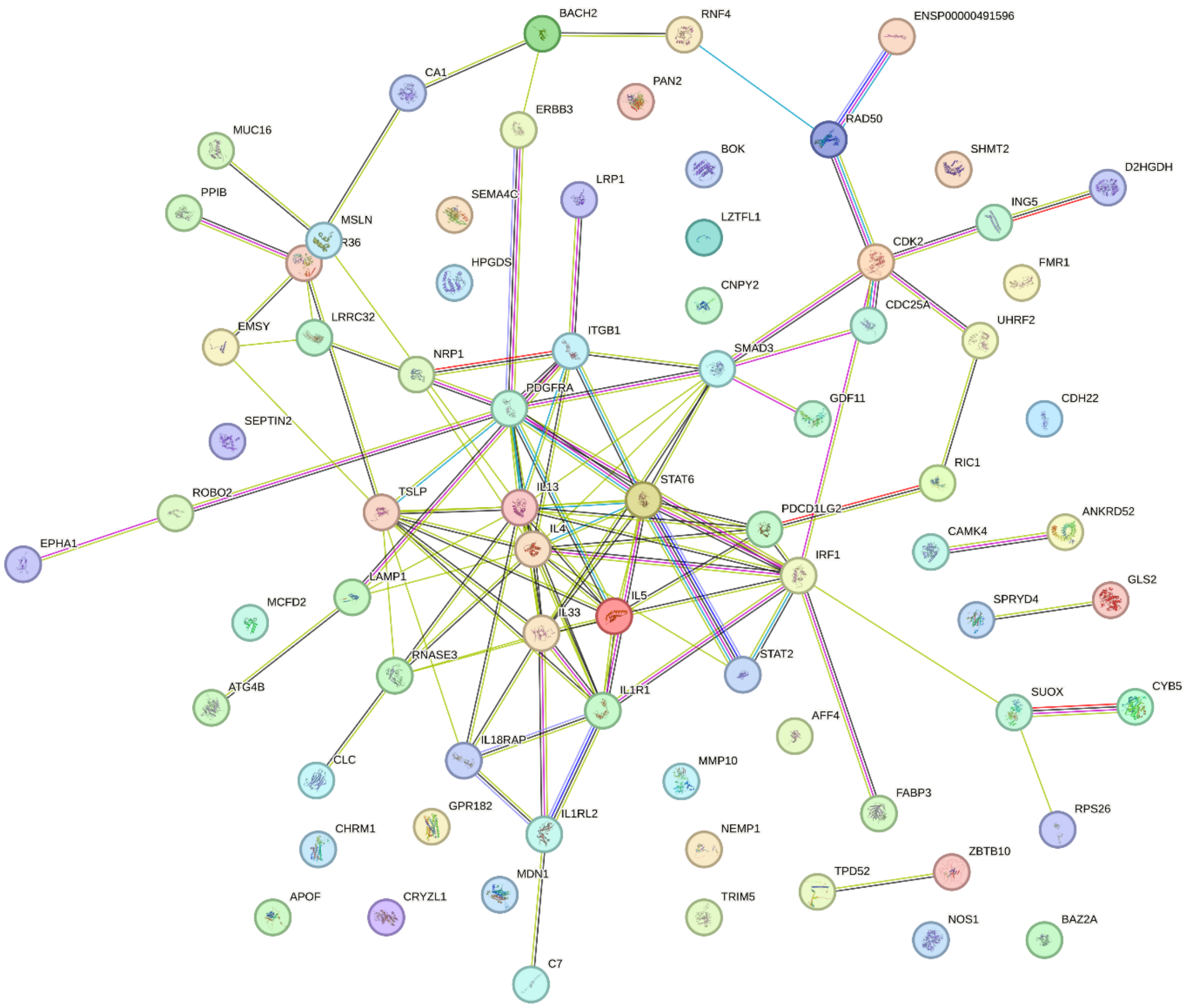

Figure S14

Q-Q plot for the GWAS of high treatment burden and/or worse outcomes vs other asthma.

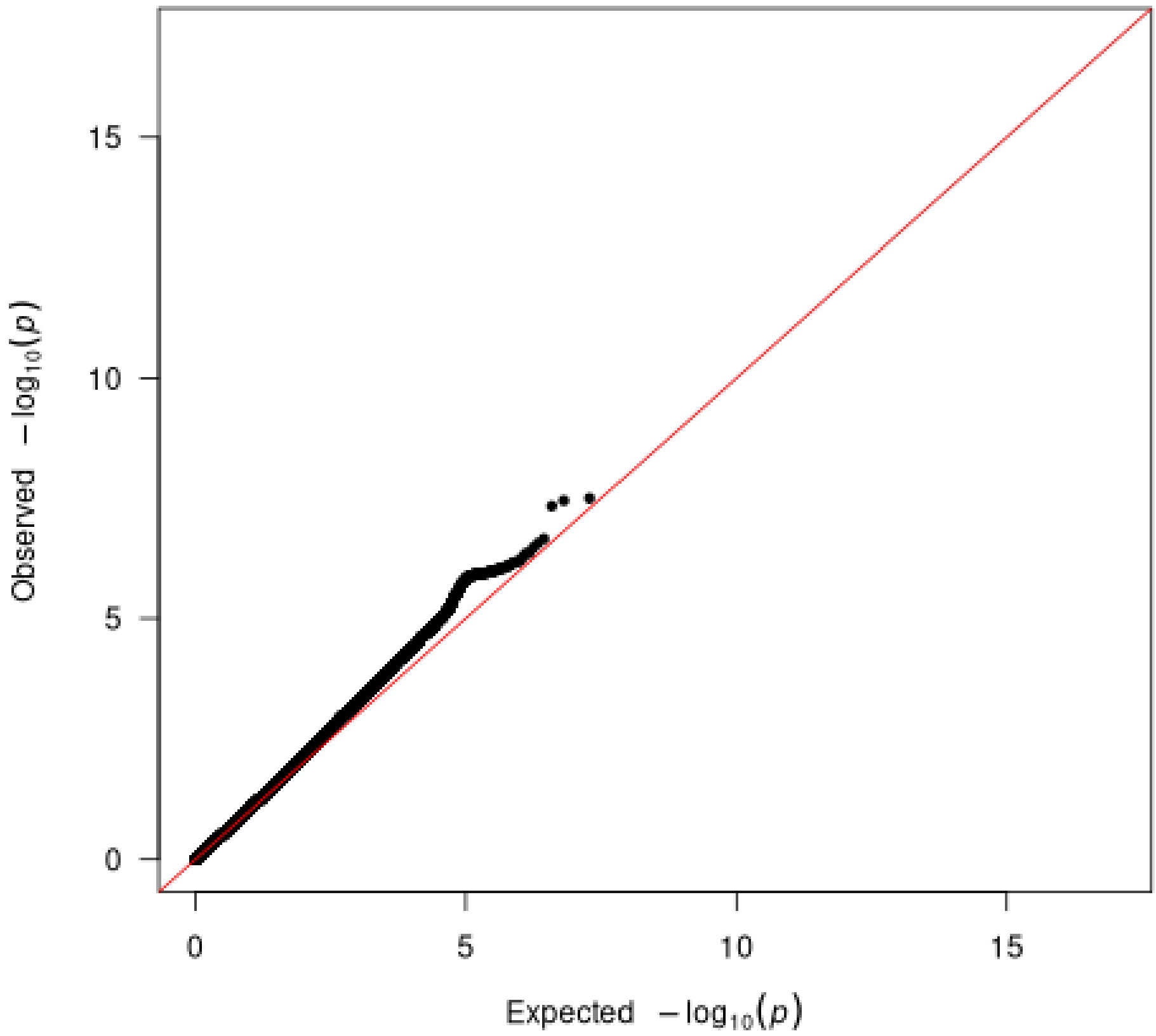

Figure S15

Manhattan plot for the GWAS of high treatment burden and/or worse outcomes vs other asthma.

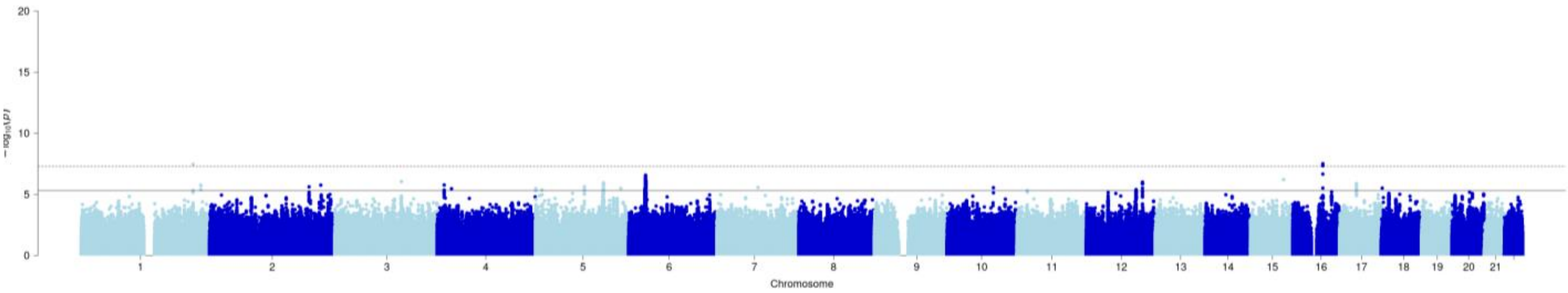

Figure S16

Scatter plot to compare genetic effect size and direction of effect for the sentinel variants between discovery GWAS and sensitivity analysis using cases and controls free of chronic bronchitits or emphysema. After removing these conditions, the genetic effect size showed a similar value and same direction of effect. The Y axis represents the log OR.

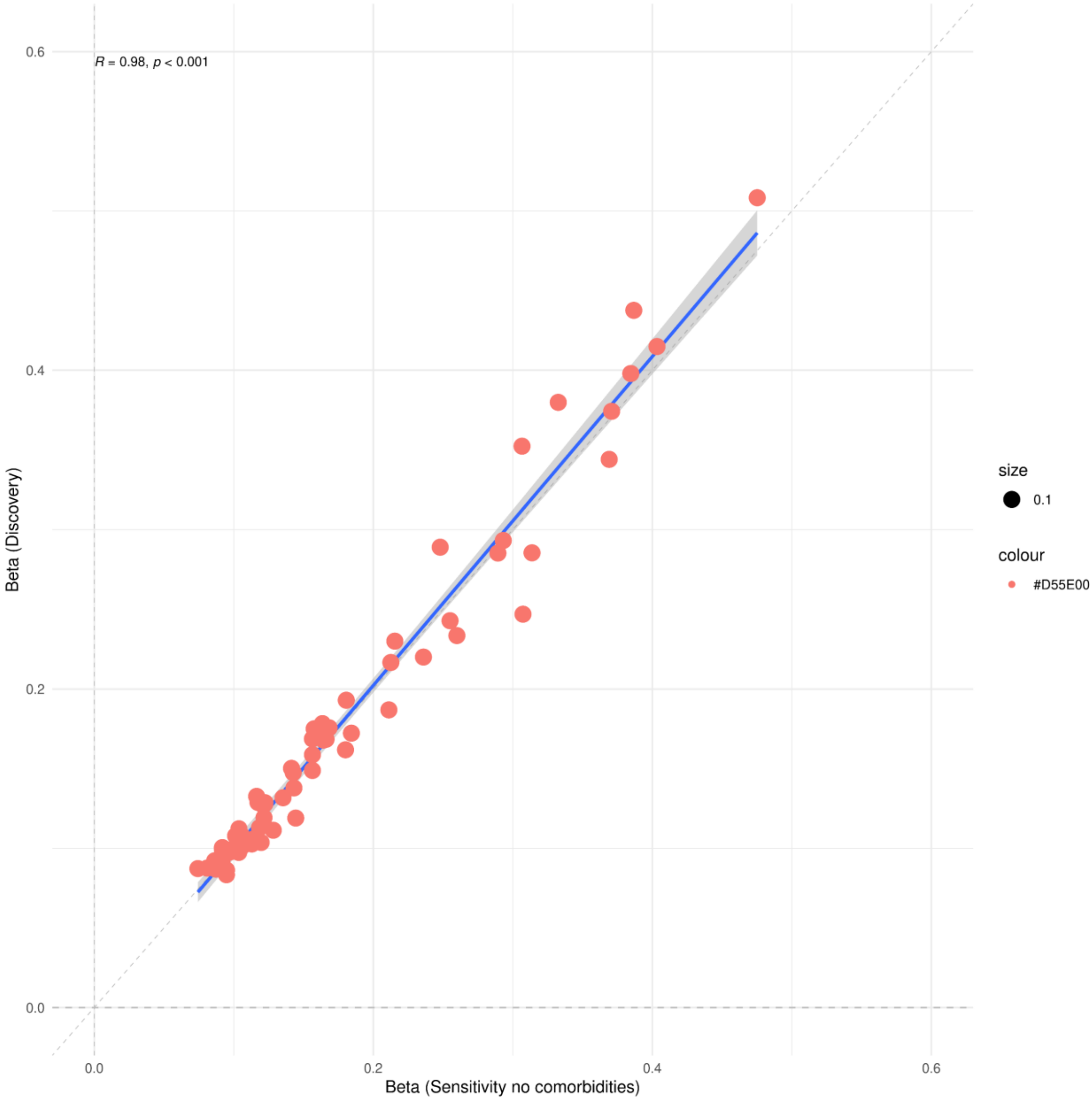

References

1. Bycroft C, Freeman C, Petkova D, Band G, Elliott LT, Sharp K, Motyer A, Vukcevic D, Delaneau O, O'Connell J, Cortes A, Welsh S, Young A, Effingham M, McVean G, Leslie S, Allen N, Donnelly P, Marchini J. The UK Biobank resource with deep phenotyping and genomic data. *Nature* 2018: 562(7726): 203-209.
2. Scottish Intercollegiate Guidelines N, British Thoracic S, Healthcare Improvement S. British guideline on the management of asthma : a national clinical guideline. Revised edition. ed. Healthcare Improvement Scotland, Edinburgh, 2019.
3. NHSBusinessServicesAuthority. Respiratory Dashboard Appendix 2.
4. Packer RJ, Williams AT, Hennah W, Eisenberg MT, Shrine N, Fawcett KA, Pearson W, Guyatt AL, Edris A, Hollox EJ, Marttila M, Rao BS, Bratty JR, Wain LV, Dudbridge F, Tobin MD. DeepPheWAS: an R package for phenotype generation and association analysis for phenome-wide association studies. *Bioinformatics* 2023: 39(4).
5. Shrine N, Guyatt AL, Erzurumluoglu AM, Jackson VE, Hobbs BD, Melbourne CA, Batini C, Fawcett KA, Song K, Sakornsakolpat P, Li X, Boxall R, Reeve NF, Obeidat M, Zhao JH, Wielscher M, Weiss S, Kentistou KA, Cook JP, Sun BB, Zhou J, Hui J, Karrasch S, Imboden M, Harris SE, Marten J, Enroth S, Kerr SM, Surakka I, Vitart V, Lehtimaki T, Allen RJ, Bakke PS, Beaty TH, Bleecker ER, Bosse Y, Brandsma CA, Chen Z, Crapo JD, Danesh J, DeMeo DL, Dudbridge F, Ewert R, Gieger C, Gulsvik A, Hansell AL, Hao K, Hoffman JD, Hokanson JE, Homuth G, Joshi PK, Joubert P, Langenberg C, Li X, Li L, Lin K, Lind L, Locantore N, Luan J, Mahajan A, Maranville JC, Murray A, Nickle DC, Packer R, Parker MM, Paynton ML, Porteous DJ, Prokopenko D, Qiao D, Rawal R, Runz H, Sayers I, Sin DD, Smith BH, Soler Artigas M, Sparrow D, Tal-Singer R, Timmers P, Van den Berge M, Whittaker JC, Woodruff PG, Yerges-Armstrong LM, Troyanskaya OG, Raitakari OT, Kahonen M, Polasek O, Gyllensten U, Rudan I, Deary IJ, Probst-Hensch NM, Schulz H, James AL, Wilson JF, Stubbe B, Zeggini E, Jarvelin MR, Wareham N, Silverman EK, Hayward C, Morris AP, Butterworth AS, Scott RA, Walters RG, Meyers DA, Cho MH, Strachan DP, Hall IP, Tobin MD, Wain LV, Understanding Society Scientific G. New genetic signals for lung function highlight pathways and chronic obstructive pulmonary disease associations across multiple ancestries. *Nat Genet* 2019: 51(3): 481-493.
6. Piga NN, Boua PR, Soremekun C, Shrine N, Coley K, Brandenburg JT, Tobin MD, Ramsay M, Fatumo S, Choudhury A, Batini C. Genetic insights into smoking behaviours in 10,558 men of African ancestry from continental Africa and the UK. *Sci Rep* 2022: 12(1): 18828.
7. Mbatchou J, Barnard L, Backman J, Marcketta A, Kosmicki JA, Ziyatdinov A, Benner C, O'Dushlaine C, Barber M, Boutkov B, Habegger L, Ferreira M, Baras A, Reid J, Abecasis G, Maxwell E, Marchini J. Computationally efficient whole-genome regression for quantitative and binary traits. *Nat Genet* 2021: 53(7): 1097-1103.
8. Jennifer Li J, Gonçalo RA. GAS Power Calculator: web-based power calculator for genetic association studies. *bioRxiv* 2017: 164343.
9. Sophia C-C, Alex M, Quanli W, Henric O, Bastian A, Glenda L, Julia L, Michael H, Yoichiro O, Monica G, Jingya W, Gundula P, Sri VVD, Graham B, Inken D, Daniel M, Suzanne C, Simon Y, Adam P, Slavé P. A broad exome study of the genetic architecture of asthma reveals novel patient subgroups. *bioRxiv* 2020: 2020.2012.2010.419663.
10. Chang D, Hunkapiller J, Bhangale T, Reeder J, Mukhyala K, Tom J, Cowgill A, Vogel J, Forrest WF, Khan Z, Stockwell A, McCarthy MI, Staton TL, Olsson J, Holweg CTJ, Cheung DS, Chen H, Brauer MJ, Graham RR, Behrens T, Wilson MS, Arron JR, Choy DF, Yaspan BL. A whole genome sequencing study of moderate to severe asthma identifies a lung function locus associated with asthma risk. *Sci Rep* 2022: 12(1): 5574.
11. Gern JE, Jackson DJ, Lemanske RF, Jr., Seroogy CM, Tachinardi U, Craven M, Hwang SY, Hamilton CM, Huggins W, O'Connor GT, Gold DR, Miller R, Kattan M, Johnson CC, Ownby D, Zoratti EM, Wood RA, Visness CM, Martinez F, Wright A, Lynch S, Ober C, Khurana Hershey GK, Ryan P, Hartert T, Bacharier LB. The Children's Respiratory and Environmental Workgroup (CREW) birth cohort consortium: design, methods, and study population. *Respir Res* 2019: 20(1): 115.

12. Fuhlbrigge A, Peden D, Apter AJ, Boushey HA, Camargo CA, Jr., Gern J, Heymann PW, Martinez FD, Mauger D, Teague WG, Blaisdell C. Asthma outcomes: exacerbations. *J Allergy Clin Immunol* 2012: 129(3 Suppl): S34-48.
13. Tsuo K, Zhou W, Wang Y, Kanai M, Namba S, Gupta R, Majara L, Nkambule LL, Morisaki T, Okada Y, Neale BM, Global Biobank Meta-analysis I, Daly MJ, Martin AR. Multi-ancestry meta-analysis of asthma identifies novel associations and highlights the value of increased power and diversity. *Cell Genom* 2022: 2(12): 100212.
14. Zhou H, Arapoglou T, Li X, Li Z, Zheng X, Moore J, Asok A, Kumar S, Blue EE, Buyske S, Cox N, Felsenfeld A, Gerstein M, Kenny E, Li B, Matise T, Philippakis A, Rehm HL, Sofia HJ, Snyder G, Group NGSPVFAW, Weng Z, Neale B, Sunyaev SR, Lin X. FAVOR: functional annotation of variants online resource and annotator for variation across the human genome. *Nucleic Acids Res* 2023: 51(D1): D1300-D1311.
15. Giambartolomei C, Vukcevic D, Schadt EE, Franke L, Hingorani AD, Wallace C, Plagnol V. Bayesian test for colocalisation between pairs of genetic association studies using summary statistics. *PLoS Genet* 2014: 10(5): e1004383.
16. Wallace C. A more accurate method for colocalisation analysis allowing for multiple causal variants. *PLoS Genet* 2021: 17(9): e1009440.
17. Consortium GT. The GTEx Consortium atlas of genetic regulatory effects across human tissues. *Science* 2020: 369(6509): 1318-1330.
18. Vosa U, Claringbould A, Westra HJ, Bonder MJ, Deelen P, Zeng B, Kirsten H, Saha A, Kreuzhuber R, Yazar S, Brugge H, Oelen R, de Vries DH, van der Wijst MGP, Kasela S, Pervjakova N, Alves I, Fave MJ, Agbessi M, Christiansen MW, Jansen R, Seppala I, Tong L, Teumer A, Schramm K, Hemani G, Verlouw J, Yaghootkar H, Sonmez Flitman R, Brown A, Kukushkina V, Kalnapenkis A, Rueger S, Porcu E, Kronberg J, Kettunen J, Lee B, Zhang F, Qi T, Hernandez JA, Arindrarto W, Beutner F, Consortium B, i QTLC, Dmitrieva J, Elansary M, Fairfax BP, Georges M, Heijmans BT, Hewitt AW, Kahonen M, Kim Y, Knight JC, Kovacs P, Krohn K, Li S, Loeffler M, Marigorta UM, Mei H, Momozawa Y, Muller-Nurasyid M, Nauck M, Nivard MG, Penninx B, Pritchard JK, Raitakari OT, Rotzschke O, Slagboom EP, Stehouwer CDA, Stumvoll M, Sullivan P, t Hoen PAC, Thiery J, Tonjes A, van Dongen J, van Iterson M, Veldink JH, Volker U, Warmerdam R, Wijmenga C, Swertz M, Andiappan A, Montgomery GW, Ripatti S, Perola M, Kutalik Z, Dermitzakis E, Bergmann S, Frayling T, van Meurs J, Prokisch H, Ahsan H, Pierce BL, Lehtimaki T, Boomsma DI, Psaty BM, Gharib SA, Awadalla P, Milani L, Ouwehand WH, Downes K, Stegle O, Battle A, Visscher PM, Yang J, Scholz M, Powell J, Gibson G, Esko T, Franke L. Large-scale cis- and trans-eQTL analyses identify thousands of genetic loci and polygenic scores that regulate blood gene expression. *Nat Genet* 2021: 53(9): 1300-1310.
19. Hao K, Bosse Y, Nickle DC, Pare PD, Postma DS, Laviolette M, Sandford A, Hackett TL, Daley D, Hogg JC, Elliott WM, Couture C, Lamontagne M, Brandsma CA, van den Berge M, Koppelman G, Reicin AS, Nicholson DW, Malkov V, Derry JM, Suver C, Tsou JA, Kulkarni A, Zhang C, Vessey R, Opiteck GJ, Curtis SP, Timens W, Sin DD. Lung eQTLs to help reveal the molecular underpinnings of asthma. *PLoS Genet* 2012: 8(11): e1003029.
20. Shrine N, Izquierdo AG, Chen J, Packer R, Hall RJ, Guyatt AL, Batini C, Thompson RJ, Pavuluri C, Malik V, Hobbs BD, Moll M, Kim W, Tal-Singer R, Bakke P, Fawcett KA, John C, Coley K, Piga NN, Pozarickij A, Lin K, Millwood IY, Chen Z, Li L, China Kadoorie Biobank Collaborative G, Wijnant SRA, Lahousse L, Brusselle G, Uitterlinden AG, Manichaikul A, Oelsner EC, Rich SS, Barr RG, Kerr SM, Vitart V, Brown MR, Wielscher M, Imboden M, Jeong A, Bartz TM, Gharib SA, Flexeder C, Karrasch S, Gieger C, Peters A, Stubbe B, Hu X, Ortega VE, Meyers DA, Bleecker ER, Gabriel SB, Gupta N, Smith AV, Luan J, Zhao JH, Hansen AF, Langhammer A, Willer C, Bhatta L, Porteous D, Smith BH, Campbell A, Sofer T, Lee J, Daviglus ML, Yu B, Lim E, Xu H, O'Connor GT, Thareja G, Albagha OME, Qatar Genome Program Research C, Suhre K, Granell R, Faquih TO, Hiemstra PS, Slats AM, Mullin BH, Hui J, James A, Beilby J, Patasova K, Hysi P, Koskela JT, Wyss AB, Jin J, Sikdar S, Lee M, May-Wilson S, Pirastu N, Kentistou KA, Joshi PK, Timmers P, Williams AT, Free RC, Wang X, Morrison JL, Gilliland FD, Chen Z, Wang CA, Foong RE, Harris SE, Taylor A, Redmond P, Cook JP, Mahajan A, Lind L, Palviainen T, Lehtimaki T,

Raitakari OT, Kaprio J, Rantanen T, Pietilainen KH, Cox SR, Pennell CE, Hall GL, Gauderman WJ, Brightling C, Wilson JF, Vasankari T, Laitinen T, Salomaa V, Mook-Kanamori DO, Timpson NJ, Zeggini E, Dupuis J, Hayward C, Brumpton B, Langenberg C, Weiss S, Homuth G, Schmidt CO, Probst-Hensch N, Jarvelin MR, Morrison AC, Polasek O, Rudan I, Lee JH, Sayers I, Rawlins EL, Dudbridge F, Silverman EK, Strachan DP, Walters RG, Morris AP, London SJ, Cho MH, Wain LV, Hall IP, Tobin MD. Multi-ancestry genome-wide association analyses improve resolution of genes and pathways influencing lung function and chronic obstructive pulmonary disease risk. *Nat Genet* 2023: 55(3): 410-422.

21. Ferkingstad E, Sulem P, Atlason BA, Sveinbjornsson G, Magnusson MI, Styrmisdottir EL, Gunnarsdottir K, Helgason A, Oddsson A, Halldorsson BV, Jensson BO, Zink F, Halldorsson GH, Masson G, Arnadottir GA, Katrinardottir H, Juliusson K, Magnusson MK, Magnusson OT, Fridriksdottir R, Saevarsdottir S, Gudjonsson SA, Stacey SN, Rognvaldsson S, Eiriksdottir T, Olafsdottir TA, Steinthorsdottir V, Tragante V, Ulfarsson MO, Stefansson H, Jonsdottir I, Holm H, Rafnar T, Melsted P, Saemundsdottir J, Norddahl GL, Lund SH, Gudbjartsson DF, Thorsteinsdottir U, Stefansson K. Large-scale integration of the plasma proteome with genetics and disease. *Nat Genet* 2021: 53(12): 1712-1721.

22. Folkersen L, Gustafsson S, Wang Q, Hansen DH, Hedman AK, Schork A, Page K, Zhernakova DV, Wu Y, Peters J, Eriksson N, Bergen SE, Boutin TS, Bretherick AD, Enroth S, Kalnapenkis A, Gadin JR, Suur BE, Chen Y, Matic L, Gale JD, Lee J, Zhang W, Quazi A, Ala-Korpela M, Choi SH, Claringbould A, Danesh J, Davey Smith G, de Masi F, Elmstahl S, Engstrom G, Fauman E, Fernandez C, Franke L, Franks PW, Giedraitis V, Haley C, Hamsten A, Ingason A, Johansson A, Joshi PK, Lind L, Lindgren CM, Lubitz S, Palmer T, Macdonald-Dunlop E, Magnusson M, Melander O, Michaelsson K, Morris AP, Magi R, Nagle MW, Nilsson PM, Nilsson J, Orho-Melander M, Polasek O, Prins B, Palsson E, Qi T, Sjogren M, Sundstrom J, Surendran P, Vosa U, Werge T, Wernersson R, Westra HJ, Yang J, Zhernakova A, Arnlov J, Fu J, Smith JG, Esko T, Hayward C, Gyllensten U, Landen M, Siegbahn A, Wilson JF, Wallentin L, Butterworth AS, Holmes MV, Ingelsson E, Malarstig A. Genomic and drug target evaluation of 90 cardiovascular proteins in 30,931 individuals. *Nat Metab* 2020: 2(10): 1135-1148.

23. Sun BB, Chiou J, Traylor M, Benner C, Hsu YH, Richardson TG, Surendran P, Mahajan A, Robins C, Vasquez-Grinnell SG, Hou L, Kvikstad EM, Burren OS, Davitte J, Ferber KL, Gillies CE, Hedman AK, Hu S, Lin T, Mikkilineni R, Pendergrass RK, Pickering C, Prins B, Baird D, Chen CY, Ward LD, Deaton AM, Welsh S, Willis CM, Lehner N, Arnold M, Worheide MA, Suhre K, Kastenmuller G, Sethi A, Cule M, Raj A, Alnylam Human G, AstraZeneca Genomics I, Biogen Biobank T, Bristol Myers S, Genentech Human G, GlaxoSmithKline Genomic S, Pfizer Integrative B, Population Analytics of Janssen Data S, Regeneron Genetics C, Burkitt-Gray L, Melamud E, Black MH, Fauman EB, Howson JMM, Kang HM, McCarthy MI, Nioi P, Petrovski S, Scott RA, Smith EN, Szalma S, Waterworth DM, Mitnaul LJ, Szustakowski JD, Gibson BW, Miller MR, Whelan CD. Plasma proteomic associations with genetics and health in the UK Biobank. *Nature* 2023: 622(7982): 329-338.

24. Weeks EM, Ulirsch JC, Cheng NY, Trippe BL, Fine RS, Miao J, Patwardhan TA, Kanai M, Nasser J, Fulco CP, Tashman KC, Aguet F, Li T, Ordovas-Montanes J, Smillie CS, Biton M, Shalek AK, Ananthakrishnan AN, Xavier RJ, Regev A, Gupta RM, Lage K, Ardlie KG, Hirschhorn JN, Lander ES, Engreitz JM, Finucane HK. Leveraging polygenic enrichments of gene features to predict genes underlying complex traits and diseases. *Nat Genet* 2023: 55(8): 1267-1276.

25. Backman JD, Li AH, Marcketta A, Sun D, Mbatchou J, Kessler MD, Benner C, Liu D, Locke AE, Balasubramanian S, Yadav A, Banerjee N, Gillies CE, Damask A, Liu S, Bai X, Hawes A, Maxwell E, Gurski L, Watanabe K, Kosmicki JA, Rajagopal V, Mighty J, Regeneron Genetics C, DiscovEhr, Jones M, Mitnaul L, Stahl E, Coppola G, Jorgenson E, Habegger L, Salerno WJ, Shuldiner AR, Lotta LA, Overton JD, Cantor MN, Reid JG, Yancopoulos G, Kang HM, Marchini J, Baras A, Abecasis GR, Ferreira MAR. Exome sequencing and analysis of 454,787 UK Biobank participants. *Nature* 2021: 599(7886): 628-634.

26. Kumar P, Henikoff S, Ng PC. Predicting the effects of coding non-synonymous variants on protein function using the SIFT algorithm. *Nat Protoc* 2009: 4(7): 1073-1081.

27. Adzhubei IA, Schmidt S, Peshkin L, Ramensky VE, Gerasimova A, Bork P, Kondrashov AS, Sunyaev SR. A method and server for predicting damaging missense mutations. *Nat Methods* 2010: 7(4): 248-249.
28. Adzhubei I, Jordan DM, Sunyaev SR. Predicting functional effect of human missense mutations using PolyPhen-2. *Curr Protoc Hum Genet* 2013: Chapter 7: Unit7 20.
29. Schwarz JM, Rodelsperger C, Schuelke M, Seelow D. MutationTaster evaluates disease-causing potential of sequence alterations. *Nat Methods* 2010: 7(8): 575-576.
30. Chun S, Fay JC. Identification of deleterious mutations within three human genomes. *Genome Res* 2009: 19(9): 1553-1561.
